# Supplementary material for: Convenient iron-catalyzed reductive aminations without hydrogen for selective synthesis of N-methylamines
Source: Nat Commun. 2017 Nov 7;8:1344. doi: 10.1038/s41467-017-01428-0 (PMC5677081; doi:10.1038/s41467-017-01428-0)
Supplement: Supplementary file 1 — Supplementary Information [file 41467_2017_1428_MOESM1_ESM.pdf]

Supplementary Tables

Supplementary Table 1. Comparison of catalytic activity for the reductive N-methylation of 4-methoxy nitrobenzene

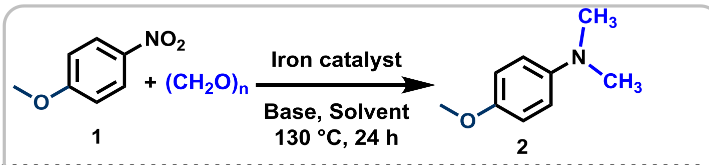

(1) $Fe(OAc)_2$=1%
(2) $Fe(OAc)_2$-L1=1%
(3) $Fe(OAc)_2$@C=1%
(4) Bulk Fe_2O_3=5%
(5) $Fe(OAc)_2$@C-800=4%
(6) $Fe(OAc)_2Al_2O_3$-800=3%
(7) $Fe(OAc)_2$@TiO_2-800=3%
(8) $Fe(OAc)_2$-L1@C=1%
(9) $Fe(OAc)_2$ -L1@C-200=5%
(10) $Fe(OAc)_2$ -L1@C-400=14%
(11) $Fe(OAc)_2$ -L1@C-600=50%
(12) $Fe(OAc)_2$ -L1@C-800=86%
(13) $Fe(OAc)_2$ -L1@C-1000=36%
(14) $Fe(OAc)_2$ -L1@Al_2O_3-800=56%
(15) $Fe(OAc)_2$ -L1@TiO_2-800=43%
(16) $Fe(OAc)_2$ -L2@C-800=23%
(17) $Fe(OAc)_2$- L3@C-800=45%
(18) $Fe(OAc)_2$ -L4@C-800=55%

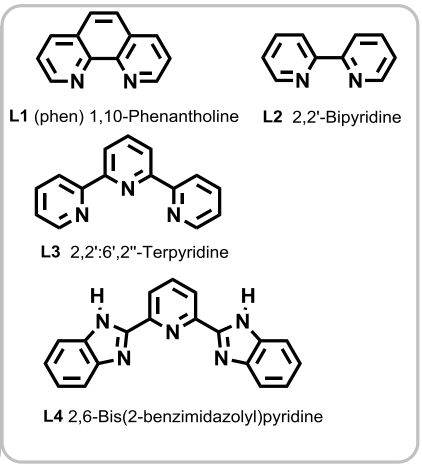

L-ligand, Fe:L=1:3, Fe=3 wt%. Materials are pyrolyzed at different temperatures (200-1000 °C) for 2 h under argon atmosphere. Reaction conditions [a]: 0.5 mmol 4-nitroanisole, weight of catalyst corresponds to 5 mol% Fe (50 mg catalyst), 10 mmol paraformaldehyde (300 mg), 1 mmol Na_2CO_3 (106 mg), 2 mL DMSO-water (1:1), 130 °C, 30 h. Yields are determined by GC using n-hexadecane as internal standard.

Supplementary Table 2. Base and solvent screening

Entry	Base	Solvent	Yield [%][b]
**Effect of base**			
1	Na_2CO_3	DMSO/H_2O (1:1)	84
2	K_2CO_3	DMSO/H_2O (1:1)	28
3	Kot*t*Bu	DMSO/H_2O (1:1)	5
4	CS_2CO_3	DMSO/H_2O (1:1)	trace
5	TEA	DMSO/H_2O (1:1)	36
6	TMEDA	DMSO/H_2O (1:1)	24
7	DBU	DMSO/H_2O (1:1)	17
8	DABCO	DMSO/H_2O (1:1)	12
9	K_3PO_4. H_2O	DMSO/H_2O (1:1)	trace
10	DIPEA	DMSO/H_2O (1:1)	16
11	DBN	DMSO/H_2O (1:1)	10
**Effect of solvent**			
13	Na_2CO_3	Acetonitrile	trace
14	Na_2CO_3	1,4 dioxane	trace
15	Na_2CO_3	DMF	trace
16	Na_2CO_3	THF	trace
17	Na_2CO_3	DMSO	31
18	Na_2CO_3	Toluene	trace
19	Na_2CO_3	Xylene	trace
20	Na_2CO_3	H_2O	trace
21	Na_2CO_3	MeOH	trace
22	Na_2CO_3	Acetone	trace
23	Na_2CO_3	Acetonitrile/ H_2O (1:1)	22
24	Na_2CO_3	1,4 dioxane/ H_2O (1:1)	14
25	Na_2CO_3	DMF/H_2O (1:1)	trace
26	Na_2CO_3	Xylene/H_2O (1:1)	trace
27	Na_2CO_3	Toluene/H_2O (1:1)	trace

Reaction conditions: TEA = triethylamine, TMDA = tetramethylethylenediamine, DBU = 1,8-diazabicyclo[5.4.0]undec-7-ene, DBN = 1,5-diazabicyclo[4.3.0]non-5-ene, DABCO = 1,4-diazabicyclo[2.2.2]octane DIPEA = N,N-diisopropylethylamine. Reaction conditions: 0.5 mmol 5-nitrobenzimadazole, 10 mmol paraformaldehyde (300 mg); 50 mg Fe_2O_3/NGr@C (5 mol % Fe), 1 mmol base, 2 mL solvent, 130 °C, 24 h.

Supplementary Methods

***N*,*N*,4-trimethylaniline**

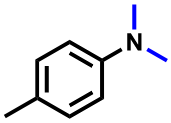

^{1}H NMR (300 MHz, Chloroform-*d*): δ 7.53–7.38 (m, 2H), 7.07 (ddd, J = 8.8, 2.8, 1.7 Hz, 2H), 3.26 (s, 6H), 2.68 (s, 3H).

^{13}C NMR (~~100~~ MHz, $CDCl_3$): δ 149.19, 129.97, 126.23, 113.53, 41.28, 20.67.

HRMS (ESI): $[M]^+$ calcd. for $C_9H_{13}N$, 136.11208; found, 136.11207.

***N*,*N*-Dimethyl-[1,1'-biphenyl]-2-amine**

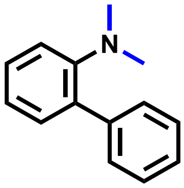

^{1}H NMR (400 MHz, Chloroform-*d*): δ 7.52–7.46 (m, 2H), 7.33–7.26 (m, 2H), 7.24–7.08 (m, 3H), 6.97– 6.88 (m, 2H), 2.46 (s, 6H).

^{13}C NMR (100 MHz, $CDCl_3$): δ 151.28, 142.04, 134.17, 131.75, 128.70, 128.33, 128.09, 126.50, 121.48, 117.59, 43.39.

HRMS (ESI): $[M]^+$ calcd. for $C_{14}H_{15}N$, 198.10341; found, 198.10339.

4-Chloro-*N*,*N*-dimethylaniline

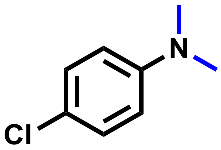

^{1}H NMR (300 MHz, Chloroform-*d*): δ 7.19 (t, J = 8.4 Hz, 1H), 6.78–6.67 (m, 2H), 6.63 (ddd, J = 8.5, 2.5, 0.9 Hz, 1H), 2.98 (s, 6H).

^{13}C NMR (~~100~~ MHz, $CDCl_3$):151.53, 135.00, 129.99, 116.18, 112.21, 110.52, 40.38.

HRMS (ESI): $[M+H]^+$ calcd. for $C_8H_{10}Cl_1N$, 156.05745; found, 156.05757.

4-Bromo-*N*,*N*-dimethylaniline

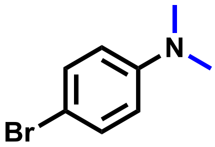

^{1}H NMR (300 MHz, Chloroform-*d*): δ 7.39–7.27 (m, 2H), 6.68–6.56 (m, 2H), 2.95 (s, 6H).

^{13}C NMR (~~100~~ MHz, $CDCl_3$): δ 149.51, 131.69, 114.12, 108.51, 40.58.

HRMS (ESI): $[M]^+$ calcd. for $C_8H_{10}BrN$, 200.00694; found, 200.0071.

***N,N,N′,N′*-Tetramethyl-p-phenylenediamine**

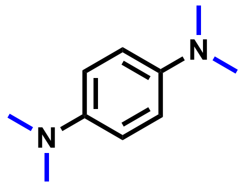

^{1}H NMR (400 MHz, Chloroform-*d*): δ 6.72 (s, 4H), 2.77 (s, 12H).

^{13}C NMR (100 MHz, $CDCl_3$): δ 115.42, 42.10.

HRMS (ESI): $[M]^+$ calcd. for $C_{10}H_{16}N_2$, 165.13862; found, 165.13877.

***N,N*-Dimethyl-4-phenoxyaniline**

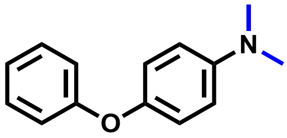

^{1}H NMR (300 MHz, Chloroform-*d*): δ 7.25 – 7.13 (m, 2H), 6.97 – 6.81 (m, 5H), 6.72 – 6.60 (m, 2H), 2.85 (s, 6H).

^{13}C NMR (100 MHz, $CDCl_3$): δ 159.10, 147.66, 147.29, 129.60, 129.45, 121.94, 120.96, 117.13, 113.97, 41.29.

HRMS (ESI): $[M]^+$ calcd. for $C_{14}H_{15}NO$, 214.12264; found, 214.12282.

3,3'-(perfluoropropane-2,2-diyl)bis(*N,N*-dimethylaniline)

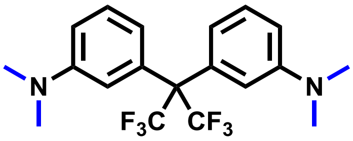

^{1}H NMR (300 MHz, Chloroform-*d*): δ 7.17 – 7.04 (m, 2H), 6.74 – 6.61 (m, 6H), 2.81 (s, 12H).

^{13}C NMR (100 MHz, $CDCl_3$): 149.40, 134.42, 128.59, 122.51, 115.34, 65.34, 65.01, 43.52, 41.02.

^{19}F NMR (282 MHz, Chloroform-*d*): δ -62.71 .

HRMS (ESI): $[M]^+$ calcd. for $C_{19}H_{20}F_6N_2$, 391.16034; found, 391.15939.

4-Ethynyl-N,N-dimethylaniline

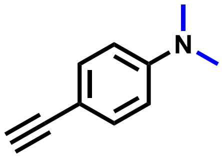

^{1}H NMR (300 MHz, Chloroform-*d*) δ 7.47 – 7.38 (m, 2H), 6.72 – 6.58 (m, 2H), 3.04 (s, 1H), 3.03 (s, 6H).

^{13}C NMR (100 MHz, CDCl3): 150.40, 133.23, 124.98, 108.75, 84.90, 74.83, 40.21.

HRMS (ESI): $[M]^+$ calcd. for $C_{10}H_{11}N$, 146.09643; found,146.09672.

_N,N_-Dimethyl-4-(methylthio)aniline

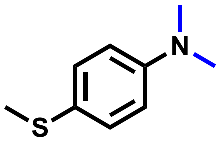

^{1}H NMR (300 MHz, Chloroform-_d_): δ 7.25 – 7.12 (m, 2H), 6.66 – 6.54 (m, 2H), 2.86 (s, 6H), 2.34 (s, 3H).

^{13}C NMR (~~100~~ MHz, $CDCl_3$): δ 149.45, 131.26, 123.38, 113.18, 40.65, 19.25.

HRMS (ESI): $[M]^+$ calcd. for $C_9H_{13}NS$, 168.08415; found, 168.08413.

3-Chloro-4-((3-fluorobenzyl)oxy)-_N,N_-dimethylaniline

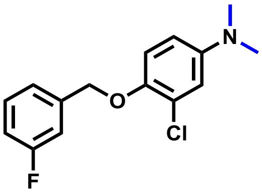

^{1}H NMR (300 MHz, Chloroform-_d_): δ 7.25 (td, J = 8.0, 5.8 Hz, 1H), 7.19 – 7.06 (m, 1H), 6.91 (ddddd, J = 8.8, 8.3, 2.6, 1.1, 0.6 Hz, 2H), 6.78 (d, J = 8.9 Hz, 1H), 6.71 (d, J = 3.0 Hz, 1H), 6.47 (dd, J = 9.0, 3.0 Hz, 1H), 4.98 – 4.93 (m, 2H), 2.79 (s, 6H).

^{13}C NMR (~~100~~ MHz, $CDCl_3$): δ164.62, 164.62, 161.36, 161.36, 146.69, 145.06, 139.94, 124.62, 122.75, 122.67, 117.37, 114.58, 114.36, 114.07, 112.02, 111.55, 71.59, 41.15, 31.19.

HRMS (ESI): $[M]^-$ calcd. for $C_{15}H_{15}ClFNO$, 278.07534; found, 278.07531.

1-(3-(Dimethylamino)phenyl)ethan-1-one

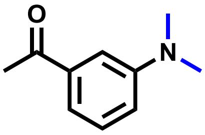

^{1}H NMR (300 MHz, Chloroform-_d_): δ 7.37–7.26 (m, 3H), 6.95 (ddd, J = 7.5, 3.0, 2.0 Hz, 1H), 3.02 (s, 6H), 2.61 (s, 3H).

^{13}C NMR (~~100~~ MHz, $CDCl_3$): δ 198.95, 150.60, 137.92, 129.14, 117.07, 116.96, 111.25, 40.55, 26.81.

HRMS (ESI): $[M]^+$ calcd. for $C_{10}H_{13}NO$, 164.14323; found, 164.14321.

(_E_)-_N,N_-Dimethyl-4-styrylaniline

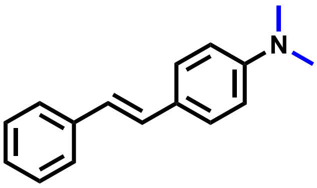

^{1}H NMR (300 MHz, Chloroform-_d_): δ 7.45–7.29 (m, 4H), 7.29–7.20 (m, 2H), 7.14–7.09 (m, 1H), 6.98 (d, J = 16.3 Hz, 1H), 6.84 (d, J = 16.3 Hz, 1H), 6.65 (d, J = 8.3 Hz, 2H), 2.91 (s, 6H).

^{13}C NMR (100 MHz, CDCl$_3$): δ 138.15, 128.76, 128.58, 127.58, 126.70, 126.02, 124.43, 112.51, 40.54.

HRMS (ESI): [M]$^+$ calcd. for $C_{16}H_{17}N$, 224.14338; found, 224.14344.

(4-(Dimethylamino)phenyl)(phenyl)methanone

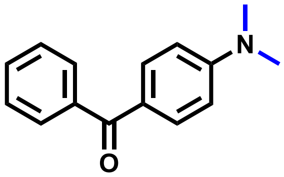

^{1}H NMR (300 MHz, Chloroform-*d*): δ 7.77 – 7.67 (m, 2H), 7.67 – 7.59 (m, 2H), 7.49 – 7.28 (m, 3H), 6.66 – 6.51 (m, 2H), 2.98 (s, 6H).

^{13}C NMR (100 MHz, CDCl$_3$): δ 195.14, 153.27, 139.30, 132.73, 131.11, 129.44, 128.01, 124.77, 110.57, 40.07.

HRMS (ESI): [M]$^+$ calcd. for $C_{15}H_{15}NO$, 225.07814; found, 225. 07811.

***N,N*-Dimethylpyridin-2-amine**

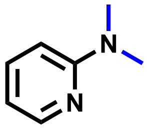

^{1}H NMR (400 MHz, Chloroform-*d*): δ 8.26 – 7.98 (m, 1H), 7.37 (ddd, J = 8.9, 7.1, 2.0 Hz, 1H), 6.58 – 6.32 (m, 2H), 3.0 (s, 6H).

^{13}C NMR (100 MHz, CDCl$_3$): δ 159.28, 147.78, 136.99, 111.36, 105.75, 37.98.

HRMS (ESI): [M]$^+$ calcd. for $C_7H_{10}N_2$, 123.09168; found, 123.09190.

***N,N*-Dimethylpyridin-4-amine**

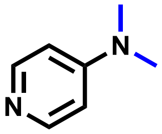

^{1}H NMR (300 MHz, Chloroform-*d*): δ 8.21 (ddq, J = 6.2, 2.9, 1.3 Hz, 2H), 6.47 (ddt, J = 5.3, 4.0, 1.6 Hz, 2H), 2.90 (s, 6H).

^{13}C NMR (100 MHz, CDCl$_3$): δ 154.17, 149.82, 106.55, 38.99.

HRMS (ESI): [M]$^+$ calcd. for $C_7H_{10}N_2$, 123.0869; found, 123.0875.

***N*,*N*,2,6-Tetramethylpyridin-3-amine**

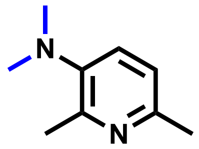

^{1}H NMR (300 MHz, Chloroform-*d*): δ 8.23 (d, *J* = 8.4 Hz, 1H), 7.21–7.08 (m, 1H), 3.29 (s, 6H), 2.87 (s, 3H), 2.66 (s, 3H).

^{13}C NMR (100 MHz, $CDCl_3$): 163.04, 153.36, 143.67, 132.91, 121.44, 37.98, 24.75, 24.13.

HRMS (ESI): $[M]^+$ calcd. for $C_9H_{14}N_2$, 151.2794; found, 151.2791.

***N*,*N*-Dimethylquinolin-8-amine**

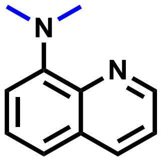

^{1}H NMR (300 MHz, Chloroform-*d*): δ 8.82 (dd, *J* = 4.2, 1.7 Hz, 1H), 8.11 (dd, *J* = 8.3, 1.7 Hz, 1H), 7.45–7.35 (m, 2H), 7.21 (dd, *J* = 8.2, 1.3 Hz, 1H), 6.98 (dd, *J* = 7.5, 1.3 Hz, 1H), 2. 99 (s, 6H).

^{13}C NMR (100 MHz, $CDCl_3$): 147.47, 144.00, 138.45, 136.03, 128.88, 127.42, 121.38, 116.06, 110.07, 41.15.

HRMS (ESI): $[M]^+$ calcd. for $C_{11}H_{12}N_2$, 173.0774; found, 173.0773.

***N*,*N*,1-Trimethyl-1*H*-pyrazol-4-amine**

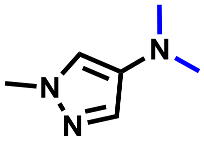

^{1}H NMR (300 MHz, Chloroform-*d*): δ 7.32 (s, 1H), δ 7.8532 (s, 1H), 3.54 (s, 3H), 2.55 (s, 6H).

^{13}C NMR (100 MHz, $CDCl_3$): δ 130.45, 130.33, 122.68, 46.61, 40.98.

HRMS (ESI): $[M]^+$ calcd. for $C_6H_{11}N_3$, 125.17551; found, 125.17549.

***N*,*N*-dimethyl-1*H*-benzo[*d*]imidazol-5-amine**

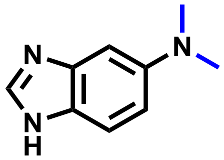

^{1}H NMR (300 MHz, DMSO-d_6): δ 12.02 (s, 1H), 7.96 (s, 1H), 7.45 – 7.32 (m, 1H), 6.82–6.71 (m, 2H), 2.87 (s, 6H).

^{13}C NMR (100 MHz, DMSO-d_6: δ 146.90, 139.80, 109.98, 40.95, 39.52.

HRMS (ESI): $[M]^+$ calcd. for $C_9H_{11}N_3$, 162.2245; found, 162.2243.

6-(Dimethylamino)-1*H*-isochromen-1-one

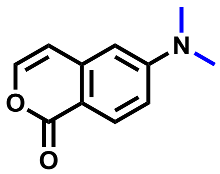

^{1}H NMR (300 MHz, Chloroform-d): δ 7.64 (d, J = 9.5 Hz, 1H), 7.22 (d, J = 9.1 Hz, 1H), 6.96 (dd, J = 9.1, 3.0 Hz, 1H), 6.67 (s,1H), 6.39 (d, J = 9.4 Hz, 1H), 2.97 (s, 6H).
^{13}C NMR (100 MHz, $CDCl_3$): δ 160.42, 146.64, 145.15, 142.76, 118.10, 116.11, 115.50, 107.94, 39.96.
HRMS (ESI): $[M]^+$ calcd. for $C_{11}H_{11}NO_2$, 190.08626; found, 190.08639.

2,5-diethoxy-*N*,*N*-dimethyl-4-morpholinoaniline

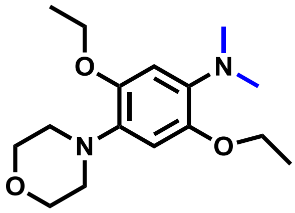

^{1}H NMR (300 MHz, Chloroform-*d*) δ 6.42 (s, 1H), 6.50 (s, 1H), 3.94 (m, J = 7.0, 2.3 Hz, 4H), 3.85 – 3.60 (m, 4H), 3.03 – 2.86 (m, 4H), 2.71 (s, 6H), 1.37 (dt, J = 11.4, 7.0 Hz, 6H).

^{13}C NMR (100 MHz, $CDCl_3$): δ 145.82, 145.54, 137.65, 135.72, 106.10, 105.10, 67.34, 64.70, 64.23, 51.32, 43.40, 15.16.

HRMS (ESI): $[M+H]^+$ calcd. for $C_{16}H_{26}N_2O_3$, 295.20162; found, 295.20181.

***N*,*N*-dimethyl-4-morpholinoaniline**

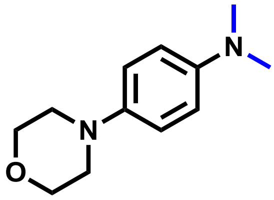

^{1}H NMR (300 MHz, Chloroform-*d*) δ 8.11 – 7.89 (m, 2H), 6.81 – 6.59 (m, 2H), 3.40 – 3.20 (m, 4H), 3.02 – 2.84 (m, 4H), 2.63 (s, 6H).

δ_C (75 MHz, $CDCl_3$) 155.18, 138.22, 125.91, 112.51, 48.07, 45.72, 43.40.

HRMS (ESI): $[M+H]^+$ calcd. for $C_{13}H_{19}N_3$, 206.1624; found, 206.1620.

Ethyl 1-(4-(dimethylamino)phenyl)-5-(trifluoromethyl)-1*H*-pyrazole-4-carboxylate

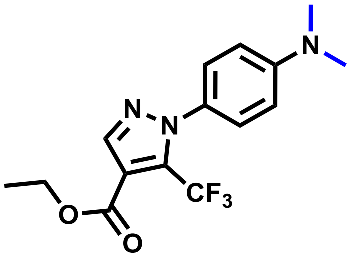

^{1}H NMR (300 MHz, Chloroform-*d*): δ 8.00 (s 1H), 7.22 – 7.12 (m, 2H), 6.70 – .60 (m, 2H), 4.29 (q, J = 7.1 Hz, 2H), 2.95 (s, 6H), 1.30 (t, J = 7.1 Hz, 3H).

^{13}C NMR (~~100~~ MHz, CDCl$_3$): δ 161.30, 151.02, 141.96, 132.94, 132.15, 128.13, 126.51, 121.05, 117.45, 116.02, 111.55, 61.15, 40.39, 14.16.

HRMS (ESI): $[M+H]^+$ calcd. for $C_{15}H_{16}F_3N_3O_2$, 328.12674; found, 328.12711.

3-Isopropyl 5-(2-methoxyethyl) 4-(3-(dimethylamino)phenyl)-2,6-dimethyl-1,4-dihydropyridine-3,5-dicarboxylate

Nimodipine-NMe$_2$

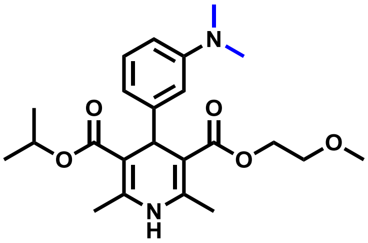

^{1}H NMR (300 MHz, Chloroform-*d*): δ 7.07 (t, J = 7.9 Hz, 1H), 6.74 (dd, J = 2.7, 1.6 Hz, 1H), 6.65 (dt, J = 7.7, 1.2 Hz, 1H), 6.53 (ddd, J = 8.2, 2.7, 0.9 Hz, 1H), 5.77 (s, 1H), 5.02– 4.88 (m, 2H), 4.27 – 4.10 (m, 2H), 3.57 (t, J = 4.9 Hz, 2H), 3.35 (s, 3H), 2.89 (s, 6H), 2.30 (d, J = 2.6 Hz, 6H), 1.24 (d, J = 6.2 Hz, 3H), 1.14 (d, J = 6.2 Hz, 3H).

^{13}C NMR (~~100~~ MHz, CDCl$_3$): δ C (75 MHz, CDCl$_3$) 167.89, 167.45, 150.62, 148.55, 144.57, 143.69, 128.72, 116.94, 113.09, 111.05, 104.89, 104.00, 70.88, 67.14, 63.10, 59.17, 41.03, 39.91, 22.39, 22.18, 19.90, 19.71.

HRMS (ESI): $[M+H]^+$ calcd. for $C_{23}H_{32}N_2O_5$, 416.12264; found, 416.12262.

3-Cinnamyl 5-(2-methoxyethyl) 4-(3-(dimethylamino)phenyl)-2,6-dimethyl-1,4-dihydropyridine-3,5-dicarboxylate

Clinidipine-NMe$_2$

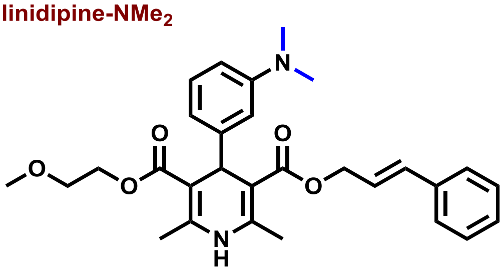

^{1}H NMR (300 MHz, Chloroform-*d*): δ 7.29–7.14 (m, 5H), 7.01 (t, J = 7.8 Hz, 1H), 6.70 (dd, J = 2.7, 1.5 Hz, 1H), 6.60 (dt, J = 7.6, 1.2 Hz, 1H), 6.46 (dtd, J = 5.2, 2.6, 0.9 Hz, 1H), 6.44– 6.38 (m, 1H), 6.16 (dt, J = 15.9, 5.9 Hz, 1H), 5.65 (s, 1H), 4.99 (s, 1H), 4.65 (qdd, J = 13.4, 6.0, 1.5 Hz, 2H),

4.17 – 4.07 (m, 2H), 3.49 (dd, *J* = 5.4, 4.4 Hz, 2H), 3.23 (s, 3H), 2.77 (s, 6H), 2.27 (s, 3H), 2.25 (s, 3H).

^{13}C NMR (100 MHz, CDCl$_3$): δ 167.60, 167.35, 150.35, 148.17, 144.42, 144.13, 136.56, 132.85, 128.71, 128.49, 127.76, 126.57, 124.13, 116.54, 112.86, 110.84, 104.12, 103.95, 70.66, 64.22, 62.91, 58.92, 40.74, 39.65, 31.92, 22.74, 19.69, 14.18.

HRMS (ESI): [M+H]$^+$ calcd. for $C_{29}H_{34}N_2O_5$, 491.25405; found, 491.25365.

3-(2-(Benzyl(methyl)amino)ethyl) 5-methyl 4-(3-(dimethylamino)phenyl)-2,6-dimethyl-1,4-dihydropyridine-3,5-dicarboxylate

Nicardipine-NMe$_2$

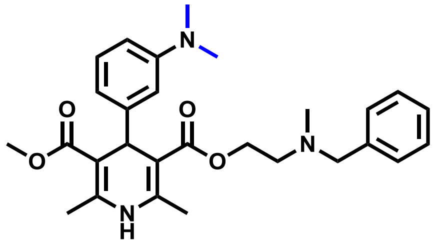

^{1}H NMR (300 MHz, Chloroform-*d*): δ 7.26–7.15 (m, 6H), 6.99 (t, J = 7.9 Hz, 1H), 6.67 – 6.62 (m, 1H), 6.56 (dt, J = 7.7, 1.2 Hz, 1H), 6.45 (ddd, J = 8.2, 2.7, 0.9 Hz, 1H), 5.54 (s, 1H), 4.95 (s, 1H), 4.14 (td, J = 5.9, 2.9 Hz, 2H), 3.57 (s, 3H), 3.45 (s, 2H), 2.80 (s, 6H), 2.67 – 2.55 (m, 2H), 2.26 (d, J = 0.8 Hz, 6H), 2.14 (s, 3H).

^{13}C NMR (100 MHz, CDCl$_3$): 168.09, 167.57, 150.41, 147.90, 144.02, 144.02, 128.95, 128.68, 128.22, 127.01, 116.21, 112.43, 110.80, 103.99, 77.44, 77.02, 76.60, 62.49, 55.66, 50.96, 42.31, 40.71, 39.33, 19.74.

HRMS (ESI): [M+H]$^+$ calcd. for $C_{28}H_{35}N_3O_4$, 478.27003; found, 478.27031.

***N*-(4-(Dimethylamino)-2-phenoxyphenyl)methanesulfonamide**

Nimisulide-NMe$_2$

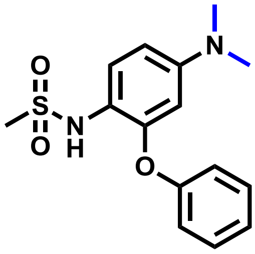

^{1}H NMR (300 MHz, Chloroform-*d*): δ 7.36 (d, J = 8.9 Hz, 1H), 7.31–7.23 (m, 2H), 7.06 (ddt, J = 7.8, 6.9, 1.1 Hz, 1H), 6.94–6.86 (m, 2H), 6.41 (dd, J = 9.0, 2.8 Hz, 1H), 6.17 (d, J = 2.8 Hz, 2H), 2.82 (s, 3H), 2.80 (s, 6H).

^{13}C NMR (100 MHz, CDCl$_3$): δ 156.45, 150.26, 149.92, 130.07, 127.26, 123.74, 117.85, 116.26, 108.43, 102.85, 102.80, 40.49, 38.94.

HRMS (ESI): [M]$^+$ calcd. for $C_{15}H_{18}N_2O_3S$, 307.11109; found; 307.11144 .

3',6'-bis(Diethylamino)-2-(4-(dimethylamino)phenyl)spiro[isoindoline-1,9'-xanthen]-3-one

Rhodamine derivative

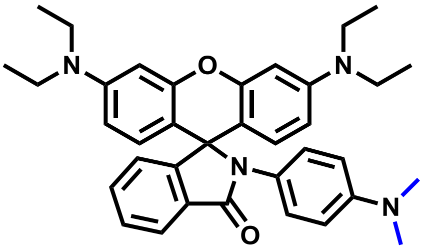

^{1}H NMR (300 MHz, Chloroform-*d*) δ 7.97–7.88 (m, 1H), 7.42–7.35 (m, 2H), 7.11–7.03 (m, 1H), 6.57 (d, J = 8.8 Hz, 2H), 6.52–6.44 (m, 2H), 6.37 (d, J = 9.1 Hz, 2H), 6.24 (dd, J = 8.9, 2.6 Hz, 2H), 6.17 (d, J = 2.6 Hz, 2H), 3.24 (qd, J = 7.2, 1.9 Hz, 8H), 2.75 (s, 6H), 1.07 (t, J = 7.0 Hz, 12H).

^{13}C NMR (100 MHz, $CDCl_3$): δ 167.76, 153.26, 149.28, 148.63, 132.46, 131.62, 129.06, 128.60, 127.99, 125.21, 124.01, 123.31, 112.47, 107.98, 106.66, 97.81, 67.27, 44.35, 40.54, 12.64.

HRMS (ESI): $[M]^+$ calcd. for $C_{36}H_{40}N_4O_2$, 561.3224; found, 561.3221.

2-(Dimethylamino)-9*H*-fluoren-9-one

Dimethylaminofluorenone

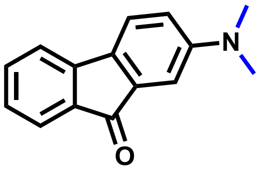

^{1}H NMR (300 MHz, Chloroform-*d*): δ 7.46 (ddd, J = 7.3, 1.2, 0.7 Hz, 1H), 7.33 – 7.16 (m, 3H), 7.02 (td, J = 7.3, 1.2 Hz, 1H), 6.95 (d, J = 2.6 Hz, 1H), 6.61 (dd, J = 8.3, 2.6 Hz, 1H), 2.93 (s, 6H).

^{13}C NMR (100 MHz, $CDCl_3$): δ 195.05, 151.28, 145.99, 135.68, 134.79, 134.19, 131.91, 126.81, 124.11, 121.18, 118.93, 116.46, 108.33, 40.63.

HRMS (ESI): $[M]^+$ calcd. for $C_{15}H_{13}NO$, 224.10699; found, 224.10727.

3-(1,1,1,3,3,3-hexafluoro-2-(3 (methylamino)phenyl)propan-2-yl)-*N,N*-dimethylaniline

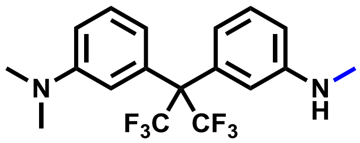

^{1}H NMR (300 MHz, Chloroform-*d*): δ 7.21–7.06 (m, 3H), 6.86 – 6.60 (m, 5H), 2.85 (s, 6H), 2.74 (s, 3H).

^{13}C NMR (100 MHz, $CDCl_3$): 13C NMR (75 MHz, CDCl3) δ 149.99, 134.37, 128.38, 126.44, 122.65, 118.81, 114.95, 112.80, 65.41, 65.08, 64.75, 40.53.

HRMS (ESI): $[M]^+$ calcd. for $C_{18}H_{18}F_6N_2$, 377.14469; found, 377.14534.

2,5-Diethoxy-*N*-methyl-4-(tetrahydro-2*H*-pyran-4-yl)aniline

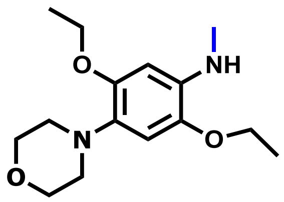

^{1}H NMR (300 MHz, Chloroform-*d*): δ 6.44 (s, 1H), 6.20 (s, 1H), 3.96 (dq, J = 18.2, 7.0 Hz, 5H), 3.87 – 3.72 (m, 4H), 3.02 – 2.85 (m, 4H), 2.77 (s, 3H), 1.33 (q, J = 7.0 Hz, 6H).

^{13}C NMR (100 MHz, $CDCl_3$): δ 146.84, 140.13, 135.79, 130.79, 104.48, 98.44, 67.46, 64.80, 64.57, 51.81, 30.86, 15.21, 15.18.

HRMS (ESI): $[M+H]^+$ calcd. for $C_{15}H_{24}N_2O_3$, 280.17814; found, 280.17832.

3',6'-Bis(diethylamino)-2-(4-(methylamino)phenyl)spiro[isoindoline-1,9'-xanthen]-3-one

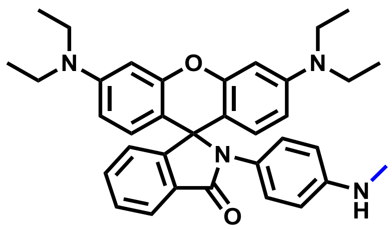

^{1}H NMR (300 MHz, Chloroform-*d*): δ 7.98 – 7.87 (m, 1H), 7.45–7.36 (m, 2H), 7.14 – 7.03 (m, 1H), 6.55 (d, J = 8.8 Hz, 2H), 6.44 – 6.37 (m, 2H), 6.29 – 6.21 (m, 4H), 6.16 (d, J = 2.6 Hz, 2H), 3.24 (qd, J = 7.2, 1.8 Hz, 8H), 2.64 (s, 3H), 1.07 (t, J = 7.0 Hz, 12H).

^{13}C NMR (100 MHz, $CDCl_3$): δ 167.76, 153.26, 149.28, 148.63, 132.46, 131.62, 129.06, 128.60, 127.99, 125.21, 124.01, 123.31, 112.47, 107.98, 106.66, 97.81, 67.27, 44.35, 40.54, 12.64.

HRMS (ESI): $[M]^+$ calcd. for $C_{35}H_{38}N_4O_2$, 547.30675; found, 547.30644.

2-(4-Aminophenyl)-3',6'-bis(diethylamino)spiro[isoindoline-1,9'-xanthen]-3-one

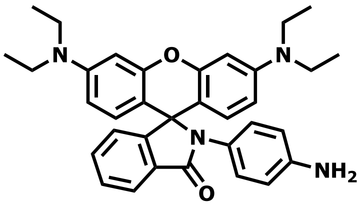

^{1}H NMR (300 MHz, Chloroform-*d*): δ 7.98 – 7.87 (m, 1H), 7.45 – 7.35 (m, 2H), 7.12 – 7.03 (m, 1H), 6.54 (d, J = 8.8 Hz, 2H), 6.41 – 6.29 (m, 4H), 6.23 (dd, J = 8.8, 2.6 Hz, 2H), 6.16 (d, J = 2.6 Hz, 2H), 3.24 (qd, J = 7.2, 1.6 Hz, 8H), 1.07 (t, J = 7.0 Hz, 12H).

^{13}C NMR (100 MHz, $CDCl_3$): 167.66, 153.30, 152.99, 148.66, 145.25, 132.55, 131.66, 129.03, 128.07, 127.06, 124.08, 123.32, 115.25, 107.99, 106.50, 97.78, 44.35, 12.62.

HRMS (ESI): $[M]^+$ calcd. for $C_{34}H_{36}N_4O_2$, 533.2911; found, 533.2912.

(*R*)-1-(2-(Dimethylamino)-1-(4-methoxyphenyl)ethyl)cyclohexan-1-ol

Venlafaxine

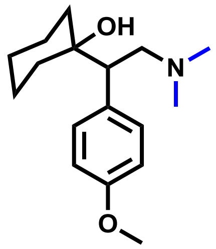

^{1}H NMR (300 MHz, Chloroform-*d*): δ 7.17–7.06 (m, 2H), 6.91-6.77 (m, 2H), 4.05 (dd, J = 12.8, 4.1 Hz, 1H), 3.79 (s, 3H), 3.56 (s, 1H), 3.36 (dd, J = 6.1, 4.1 Hz, 1H), 3.17 (dt, J = 12.6, 6.2 Hz, 1H), 2.81 (d, J = 4.4 Hz, 3H), 2.62 (d, J = 4.1 Hz, 3H), 1.79–1.43 (m, 7H), 1.36–1.06 (m, 2H), 1.00 – 0.80 (m, 1H).

^{13}C NMR (100 MHz, $CDCl_3$): δ 158.89, 131.35, 130.17, 114.07, 99.98, 73.54, 60.39, 55.26, 52.51, 45.10, 42.61, 36.65, 31.44, 25.34, 21.58, 21.18.

HRMS (ESI): $[M]^+$ calcd. for $C_{17}H_{27}NO_2$, 278.3342; found, 278.3340.

(*R*)-*N*-Methyl-*N*-(1-(naphthalen-1-yl)ethyl)-3-(3-(trifluoromethyl)phenyl)propan-1-amine

Cinacalcet-NMe

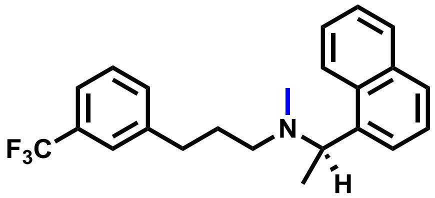

^{1}H NMR (300 MHz, Chloroform-*d*): δ 8.37– 8.23 (m, 1H), 7.76 – 7.69 (m, 1H), 7.62 (dt, J = 8.3, 1.0 Hz, 1H), 7.45 – 7.23 (m, 6H), 7.21 – 7.10 (m, 1H), 7.02 (dddd, J = 7.6, 1.9, 1.3, 0.7 Hz, 1H), 4.18 (q, J = 6.7 Hz, 1H), 2.47 – 2.25 (m, 4H), 2.18 (s, 3H), 1.72 – 1.56 (m, 2H), 1.34 (d, J = 6.7 Hz, 3H).

^{13}C NMR (100 MHz, $CDCl_3$): δ 142.4, 139.7, 133.3, 133.0, 130.8, 130.7, 127.6, 127.5, 126.6, 126.3, 124.4, 124.3, 124.3, 123.4, 121.4, 59.5, 52.5, 37.6, 32.1, 28.0, 15.7.

HRMS (ESI): [M] calcd. for $C_{23}H_{24}F_3N$, 371.18554; found, 371.18477.

3-(10,11-Dihydro-5*H*-dibenzo[*a*,*d*][7]annulen-5-yl)-*N*,*N*-dimethylpropan-1-amine

Imipramine

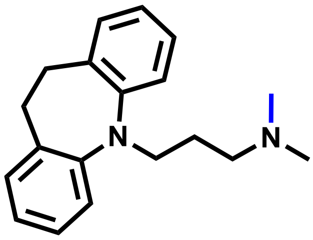

^{1}H NMR (400.1 MHz, $CDCl_3$): δ 6.92-6.97 (m, 8H); 3.78-3.82 (t, 2H), 3.18 (s, 4H), 2.39-2.42 (t, 2H), 2.21 (s, 6H), 1.77-1.80 (t, 2H).

^{13}C NMR (100 MHz, CDCl$_3$): δ 148.21, 134.21, 129.81, 126.39, 122.48, 119.96, 57.50, 48.72, 45.16, 35.22, 25.7,

HRMS (ESI): [M] calcd. for $C_{19}H_{24}N_2$ 280.304; found, 280.213.

3-(10,11-Dihydro-5*H*-dibenzo[*a*,*d*][7]annulen-5-ylidene)-*N*,*N*-dimethylpropan-1-amine

Amitriptyline

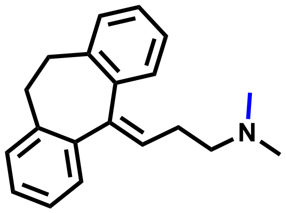

^{1}H NMR (400.1 MHz, CDCl$_3$): δ 7.21-7.19 (m, 1H), 7.12-7.01 (m, 6H), 6.94 (t, J = 8.4 Hz 1H), 5.80 (t, J = 7.1 Hz, 1H), 3.25 (br, 2H), 2.74 (br, 2H), 2.30 (d, J = 10.7 Hz, 2H), 2.28 (t, J = 8.7 Hz, 2H), 2.09 (s, 6H).

^{13}C NMR (100 MHz, CDCl$_3$): δ 143.7, 141.2, 140.0, 139.3, 137.0, 129.8, 128.9, 128.6, 128.1, 128.0, 127.4, 127.0, 126.0, 125.7, 59.3, 45.1, 33.8, 32.0, 27.6.

HRMS (ESI): $[M]^+$ calcd. for $C_{20}H_{23}N$, 278.1913; found, 278.1909.

3-Ethyl 5-methyl (*R*)-4-(2-chlorophenyl)-2-((2-(dimethylamino)ethoxy)methyl)-6-methyl-1,4-dihydropyridine-3,5-dicarboxylate

Amoldipine-NMe2

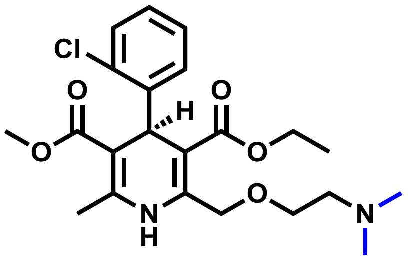

^{1}H NMR (300 MHz, Chloroform-*d*): δ 8.32 (s, 1H), 7.32–7.25 (m, 1H), 7.15 (dd, J = 7.8, 1.5 Hz, 1H), 7.06 (td, J = 7.5, 1.5 Hz, 1H), 7.01–6.93 (m, 1H), 5.32 (s, 1H), 4.76–4.57 (m, 2H), 3.97 (qd, J = 7.1, 1.7 Hz, 2H), 3.64–3.54 (m, 2H), 3.53 (s, 3H), 2.53–2.45 (m, 1H), 2.26 (d, J = 1.0 Hz, 9H), 1.11 (t, J = 7.1 Hz, 3H).

^{13}C NMR (100 MHz, CDCl$_3$): δ 167.97, 167.11, 146.41, 145.72, 144.61, 132.12, 131.35, 129.00, 127.06, 126.56, 103.23, 100.83, 68.83, 68.00, 59.48, 58.66, 50.50, 40.68, 37.21, 18.73, 14.09.

HRMS (ESI): [M] calcd. for $C_{22}H_{29}O_5N_2Cl$, 436.17595; found, 436.17600.

(1*S*,4*S*)-4-(3,4-Dichlorophenyl)-*N*,*N*-dimethyl-1,2,3,4-tetrahydronaphthalen-1-amine

Sertraline-NMe

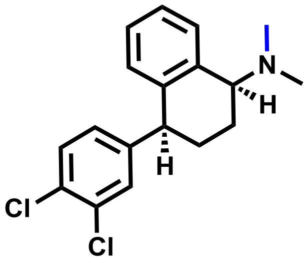

^{1}H NMR (300 MHz, Chloroform-*d*): 7.75 (d, J = 9.0 Hz, 1H), 7.35 (d, J = 8.2 Hz, 1H), 7.28 (m, 1H), 7.19-7.15 (m, 1H), 6,93-6.87 (m, 2H), 4.13 (t, J = 5.2 Hz, 1H), 3.81 (t, J = 14 Hz, 1H), 2.36 (s, 6H), 2.20-2.03 (m, 2H), 1.81-1.69 (m, 2H).

^{13}C NMR (100 MHz, CDCl$_3$):146.7, 138.1, 137.1, 131.1, 129.7, 129.1, 128.9, 128.8 127.9, 127.2, 125.9, 125.7, 61.6, 42.9, 39.9, 28.7, 14.9.

HRMS (ESI): [M] calcd. for $C_{18}H_{19}N_1Cl_2$, 319.08891; found, 319.08822.

Supplementary Figures

Qualitative Compound Report

**Instrument Name**	ESI-TOF/MS	**Date Filename**	D:\MassHunter\Data\1511\15110305.d
**Acq Method**	HRMS Pos oS.m	**Sample Name**	NK4142
**DA Method**	HRMS.m	**Position**	Vial 72
**User Name**	Fischer	**Comment**	MeOH/0,1%HCOOH in H2O 90:10

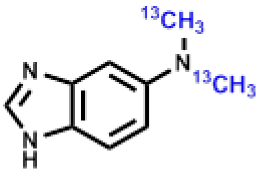

Compound Table

Name	RT	Abund	Formula	Ion Mass	Ionization Mode
1	0.189	171041	C7 [13C]2 H11 N3	163.10201	Positive

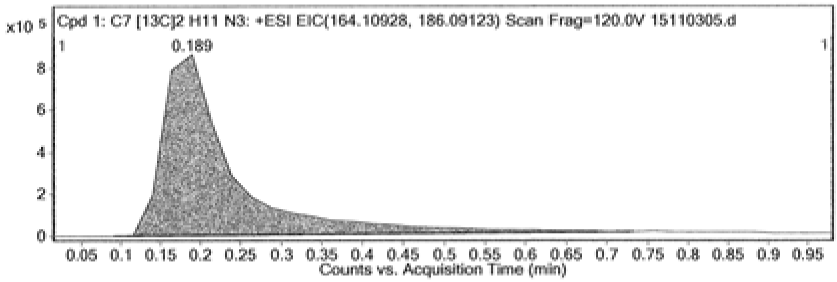

MS Zoomed Spectrum

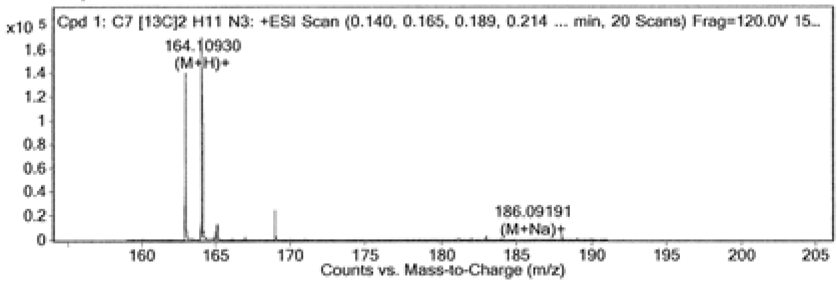

MS Spectrum Peak List

Ion	Abund	Formula	Calculated Mass	Measured Mass	Difference	Diff (ppm)
(M+H)+	171041.25	C7[13C]2H11N3	164.10928	164.1093	-0.02	-0.1

--- End Of Report ---

Supplementary Figure 1. HRMS of ^{13}C labeled *N*, *N*-dimethyl-1*H*-benzo[d]imidazol-5-amine

MassLib/PC

Cur.©Wiley WGen.©Wiley 30.10.2015 17:06

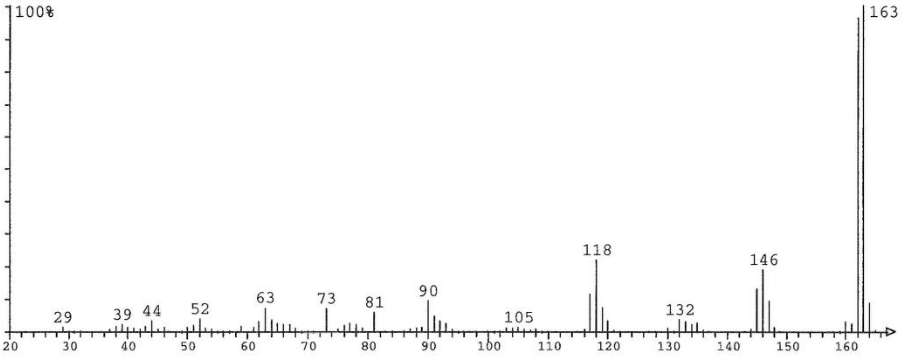

B1760(20:00) 2 25% 15103004 NK 4142

29	0.98	55	0.25	81	5.69	105	1.02	135	2.41
30	0.09	56	0.14	83	0.08	106	0.44	136	0.30
31	0.20	57	0.12	84	0.08	107	0.36	137	0.03
32	0.11	59	1.44	86	0.18	108	0.39	143	0.05
37	0.52	61	1.10	87	0.39	109	0.12	144	0.42
38	1.28	62	2.70	88	0.89	110	0.03	145	13.07
39	1.83	63	7.04	89	1.07	115	0.04	146	18.84
40	1.09	64	3.38	90	9.12	116	0.60	147	9.16
41	0.68	65	2.38	91	4.55	117	11.19	148	0.97
42	0.62	66	1.95	92	3.18	118	21.72	149	0.06
43	1.32	67	2.10	93	2.20	119	7.26	158	0.03
44	3.18	68	0.77	94	0.56	120	3.19	159	0.10
45	0.38	69	0.12	95	0.16	121	0.35	160	2.70
46	1.08	70	0.27	96	0.03	122	0.06	161	2.11
47	0.06	71	0.15	97	0.04	127	0.02	162	96.15
49	0.09	73	6.77	98	0.04	128	0.04	163	100.00
50	1.04	75	0.47	100	0.06	130	0.72	164	8.55
51	1.55	76	1.60	101	0.09	131	0.28	165	0.36
52	3.70	77	2.12	102	0.10	132	3.40	167	0.02
53	0.91	78	1.92	103	0.82	133	2.96		
54	0.51	79	0.91	104	0.70	134	2.07		

B1760(20:00) 2 25% 15103004 NK 4142

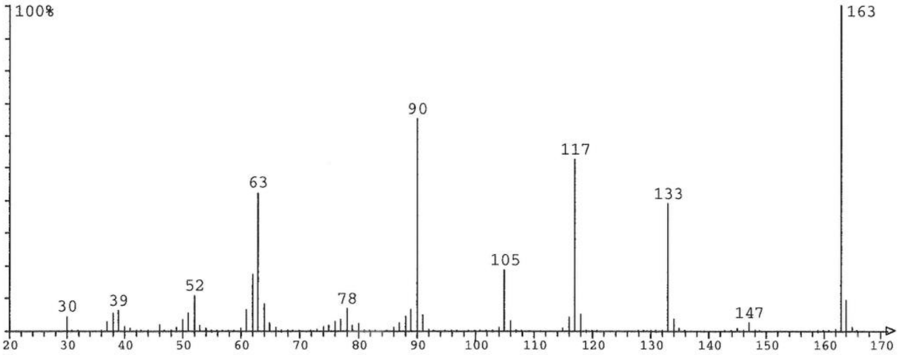

B2021(22:06) 2 21% 15103004 NK 4142

30	4.12	55	0.12	77	3.02	101	0.07	134	3.35
31	0.06	56	0.05	78	6.51	102	0.04	135	0.40
32	0.02	57	0.05	79	1.34	103	0.22	136	0.05
36	0.20	58	0.04	80	1.98	104	0.78	143	0.02
37	2.68	59	0.05	81	0.17	105	18.43	144	0.07
38	5.04	60	0.55	82	0.01	106	2.83	145	0.38
39	5.91	61	6.29	83	0.04	107	0.14	146	0.17
40	1.24	62	16.96	85	0.05	108	0.05	147	2.29
41	0.40	63	42.13	86	0.69	115	0.43	148	0.18
42	0.14	64	8.18	87	2.35	116	4.00	149	0.06
43	0.06	65	2.19	88	4.33	117	52.47	159	0.08
44	0.27	66	0.93	89	6.35	118	4.79	160	0.07
46	1.53	67	0.23	90	64.93	119	0.25	161	0.04
47	0.03	68	0.20	91	4.68	120	0.04	162	0.31
48	0.11	69	0.01	92	0.29	121	0.04	163	100.00
49	0.69	70	0.03	93	0.07	128	0.07	164	9.12
50	3.19	72	0.06	95	0.04	129	0.04	165	0.79
51	5.27	73	0.34	96	0.04	130	0.11	166	0.05
52	10.52	74	1.07	97	0.04	131	0.17		
53	1.27	75	1.41	99	0.04	132	0.18		
54	0.41	76	2.46	100	0.08	133	38.91		

B2021(22:06) 2 21% 15103004 NK 4142

MassLib/PC V9.2 3063

Admin,IfOK, Rostock

Supplementary Figure 2. GC-MS of ^{13}C labeled *N*, *N*-dimethyl-1*H*-benzo[d]imidazol-5-amine

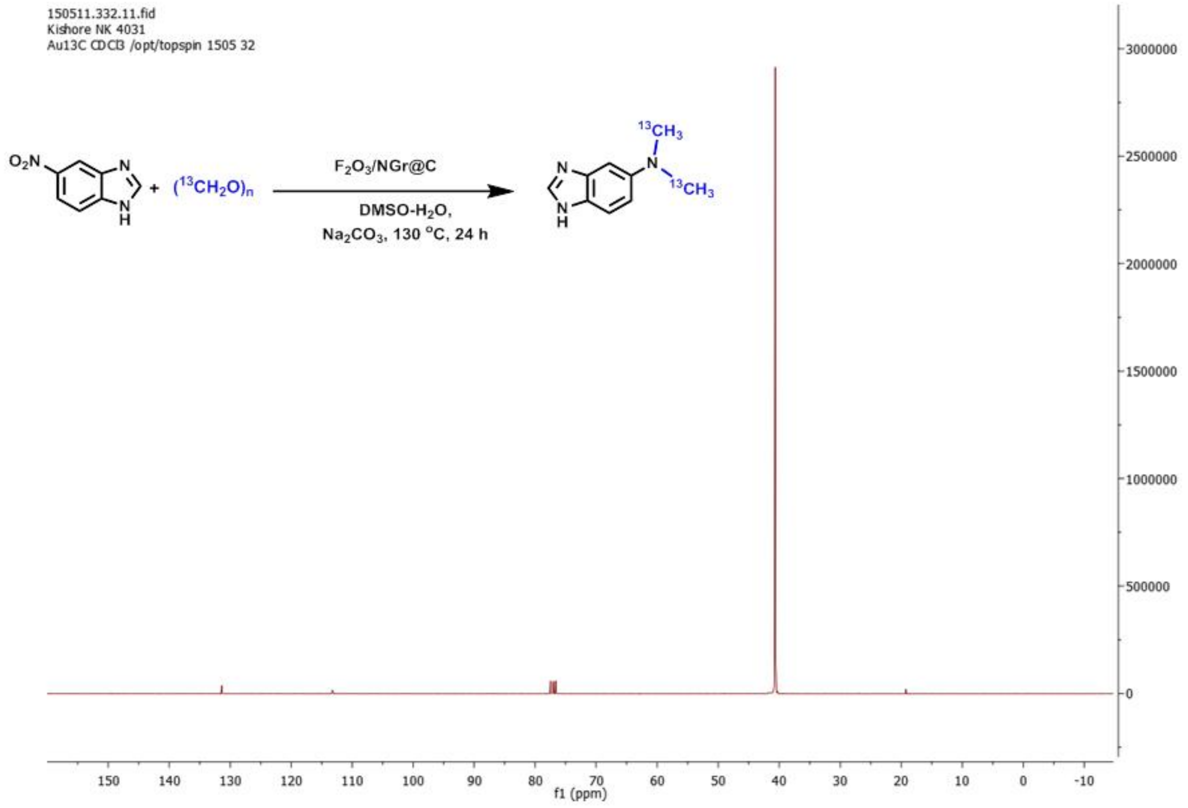

Supplementary Figure 3. ^{13}C NMR of ^{13}C labeled *N*, *N*-dimethyl-1*H*-benzo[d]imidazol-5-amine

Data File C:\HPCHEM\1\DATA\2015\1501\150408\SIG15516.D Sample Name: AR03-FeBr-Ir3

1 µmol FeBr2, IrPS, bpy, 5h, R2

===

Injection Date : 10/04/2015 14:01:51 PM
Sample Name : AR03-FeBr-Ir3 Location : Vial 1
Acq. Operator : AR Inj : 1
Inj Volume : Manually
Acq. Method : C:\HPCHEM\1\METHODS\WASSERST.M
Last changed : 15/08/2013 09:05:09 PM by AK
Analysis Method : C:\HPCHEM\1\METHODS\CAL1404.M
Last changed : 10/04/2015 15:41:53 PM by CC
(modified after loading)

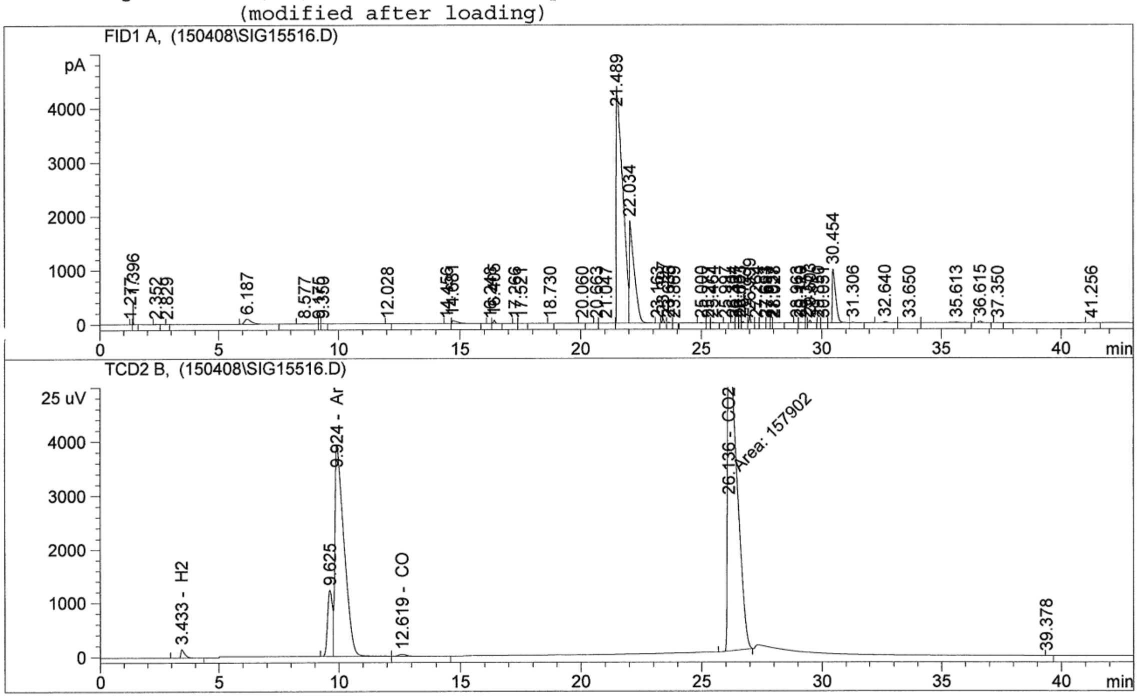

===

External Standard Report

===

Sorted By : Retention Time
Calib. Data Modified : 10/04/2015 15:41:53 PM
Multiplier : 1.0000
Dilution : 1.0000
Sample Amount : 1.00000 [vol%] (not used in calc.)

Signal 1: FID1 A,
Signal 2: TCD2 B,

RetTime [min]	Sig	Type	Area	Amt/Area	Amount [vol%]	Grp	Name
3.433	2	PB	1789.18909	1.35596e-2	24.26065		H2
9.924	2	VV	1.03703e5	2.24577e-4	23.28933		Ar
12.619	2	VB	866.54584	2.23092e-4	1.93319e-1		CO
19.900	2		-	-	-		CH4
26.136	2	MM	1.57902e5	1.97618e-4	31.20429		CO2

Totals : 78.94759

Results obtained with enhanced integrator!
1 Warnings or Errors :

Warning : Calibrated compound(s) not found

===

Gas GC 10/04/2015 15:43:06 PM CC Page 1 of 1

Supplementary Figure 4. GC-spectrum for the detected gases

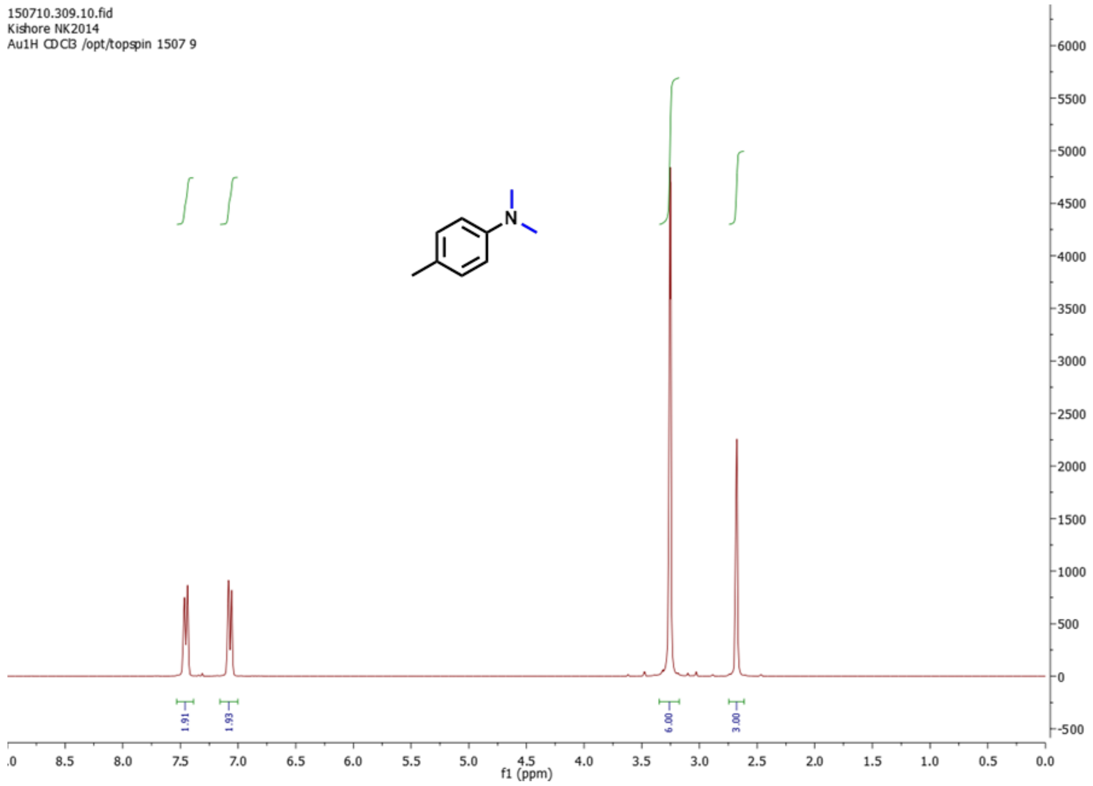

Supplementary Figure 5. ^{1}H NMR of *N*,*N*,4-Trimethylaniline

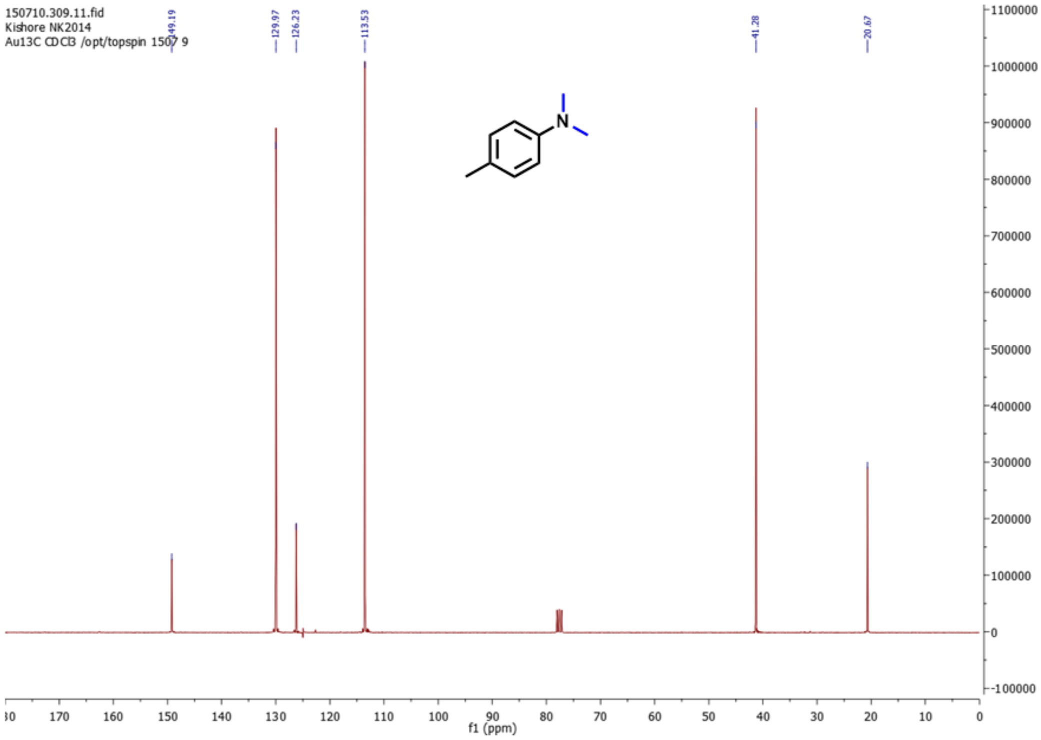

Supplementary Figure 6. ^{13}C NMR of of *N*,*N*,4-Trimethylaniline

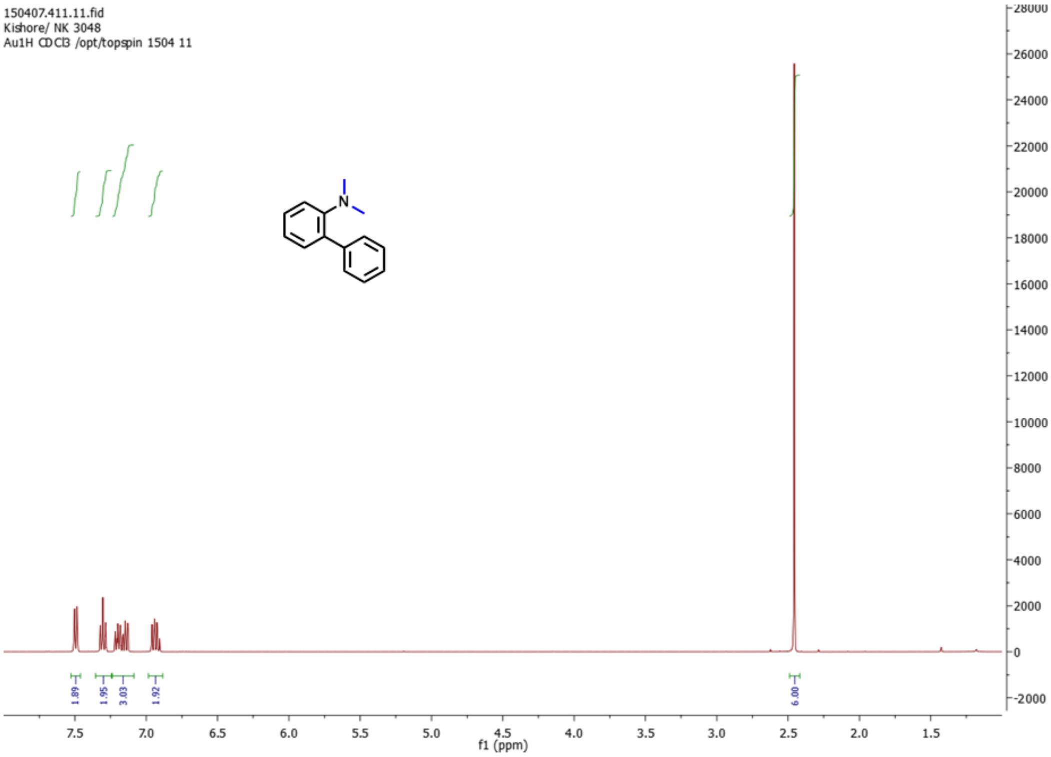

Supplementary Figure 7. ^{1}H NMR of *N*,*N*-Dimethyl-[1,1'-biphenyl]-2-amine

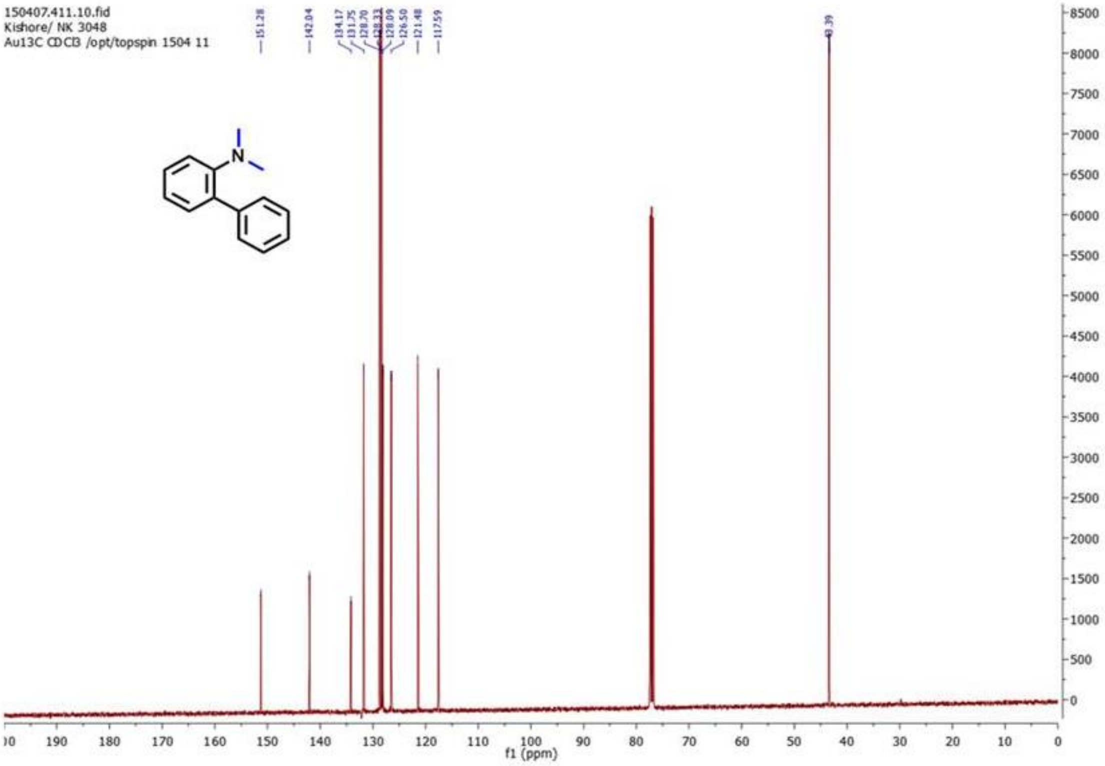

Supplementary Figure 8. ^{13}C NMR of *N*,*N*-Dimethyl-[1,1'-biphenyl]-2-amine

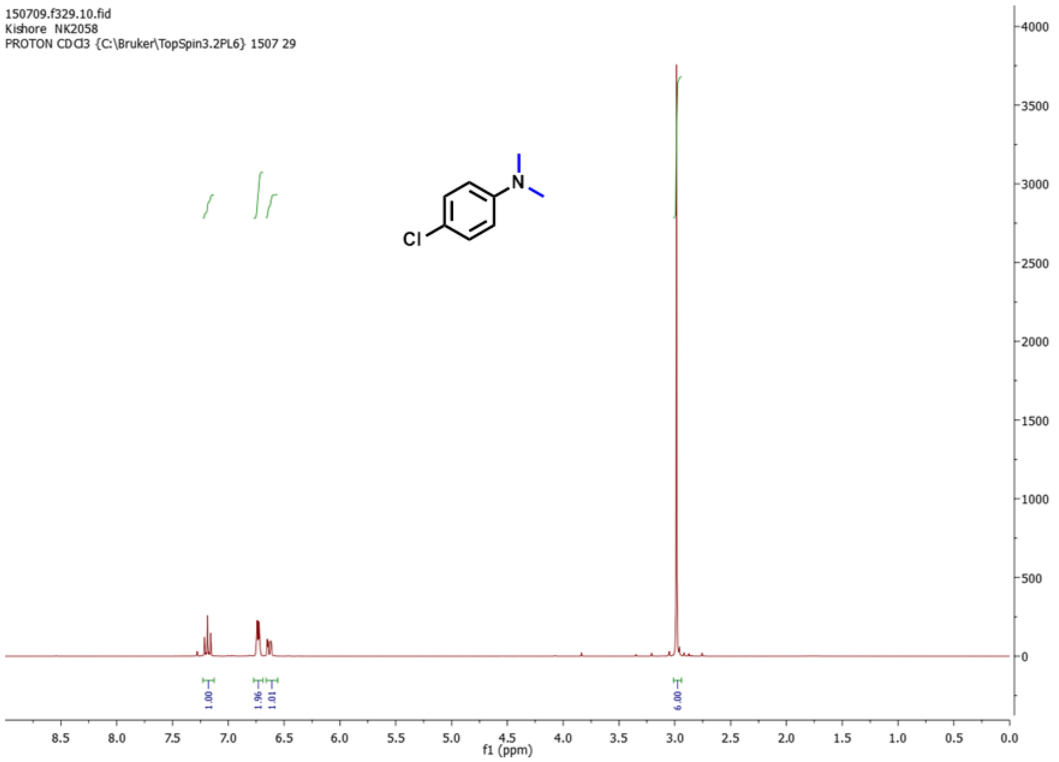

Supplementary Figure 9. ^{1}H NMR of 4-Chloro-*N*,*N*-dimethylaniline

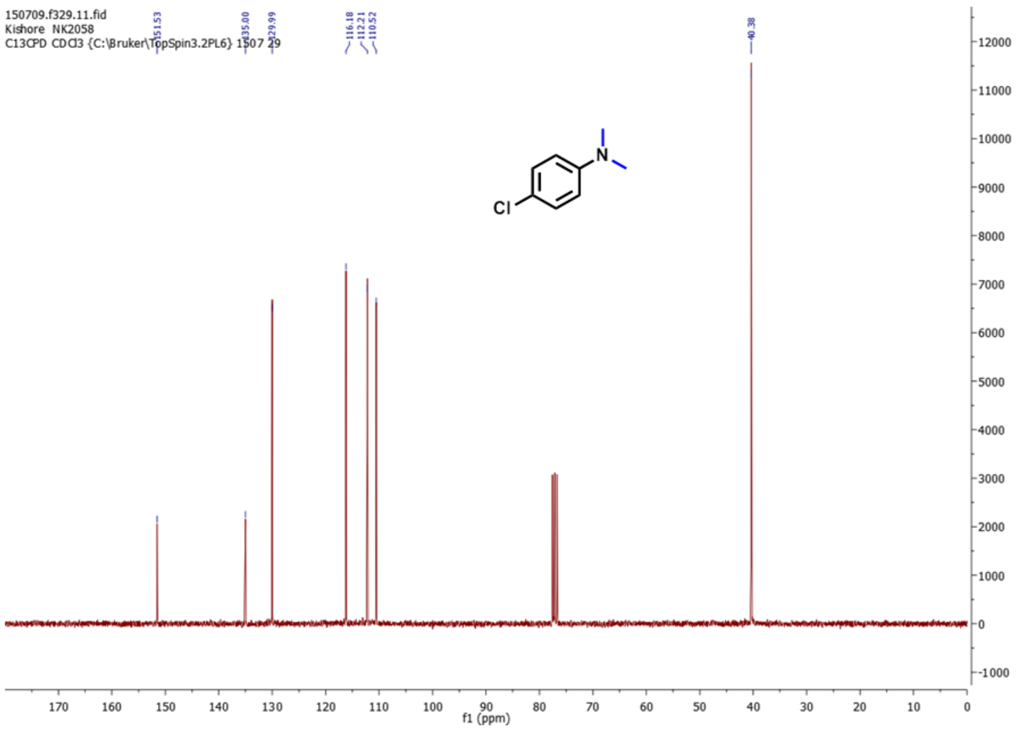

Supplementary Figure 10. ^{13}C NMR of 4-Chloro-*N*,*N*-dimethylaniline

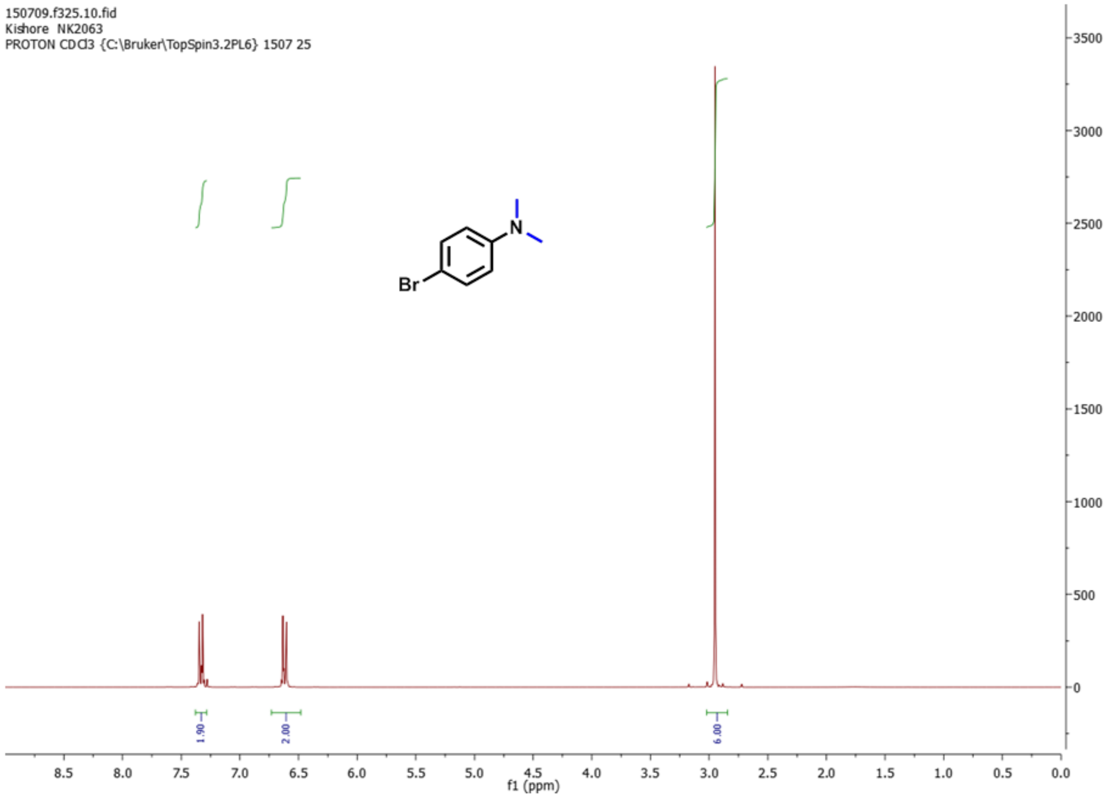

Supplementary Figure 11. ^{1}H NMR of 4-Bromo-*N*,*N*-dimethylaniline

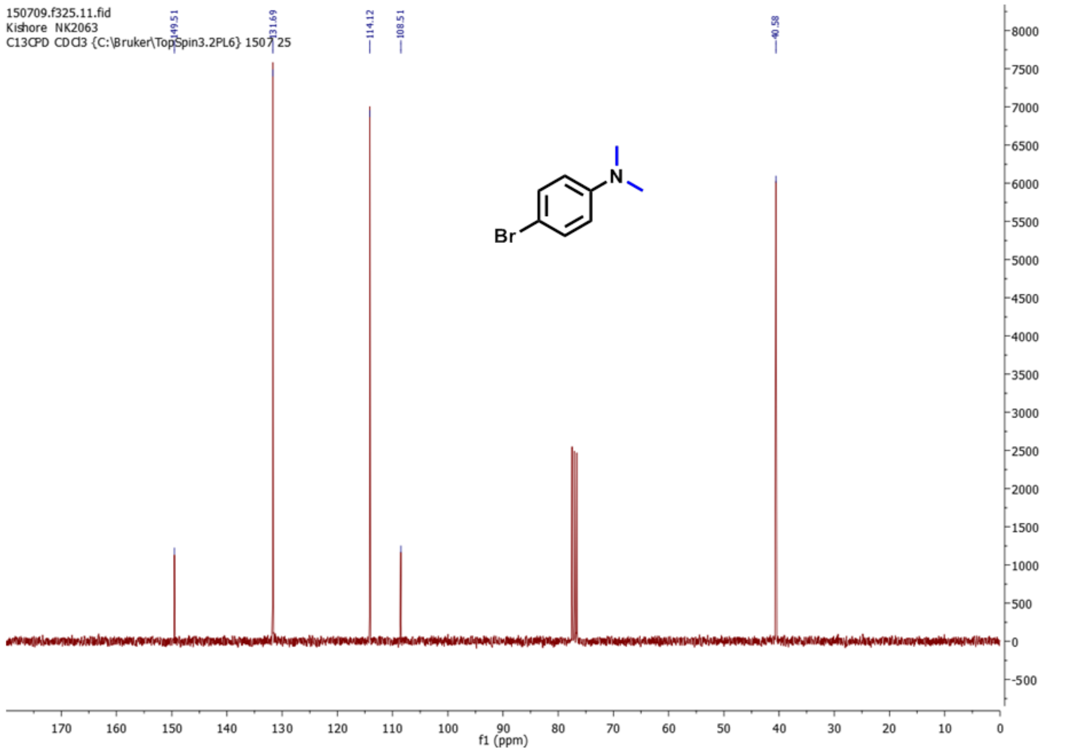

Supplementary Figure 12. ^{13}C NMR of 4-Bromo-*N*,*N*-dimethylaniline

Qualitative Compound Report

**Instrument Name**	ESI-TOF/MS	**Date Filename**	D:\MassHunter\Data\1505\15052014.d
**Acq Method**	HRMS Pos oS.m	**Sample Name**	NK2063
**DA Method**	HRMS.m	**Position**	Vial 75
**User Name**	Fischer	**Comment**	MeOH/0,1%HCOOH in H2O 90:10

Compound Table

Name	RT	Abund	Formula	Ion Mass	Ionization Mode
1	0.168	786923	C8 H10 Br N	198.9997	Positive

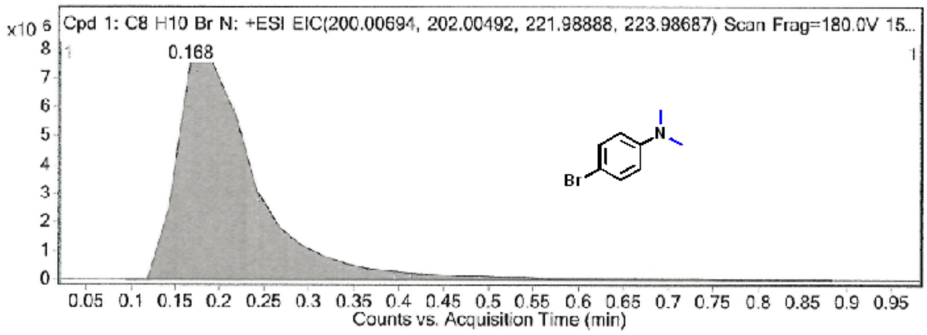

MS Zoomed Spectrum

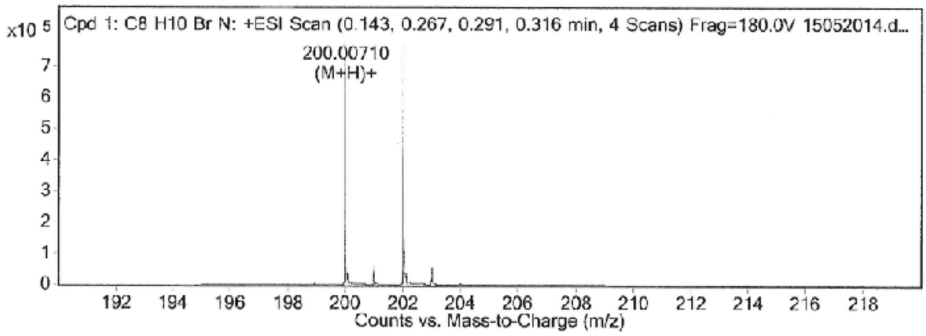

MS Spectrum Peak List

Ion	Abund	Formula	Calculated Mass	Measured Mass	Difference	Diff (ppm)
(M+H)+	786923.44	C8H10BrN	200.00694	200.0071	-0.16	-0.79
(M+H)+	771144.69	C8H10BrN	202.00492	202.00518	-0.26	-1.29

--- End Of Report ---

Supplementary Figure 13. HRMS (High Resolution Mass Spectroscopy) of 4-Bromo-*N*,*N*-dimethylaniline

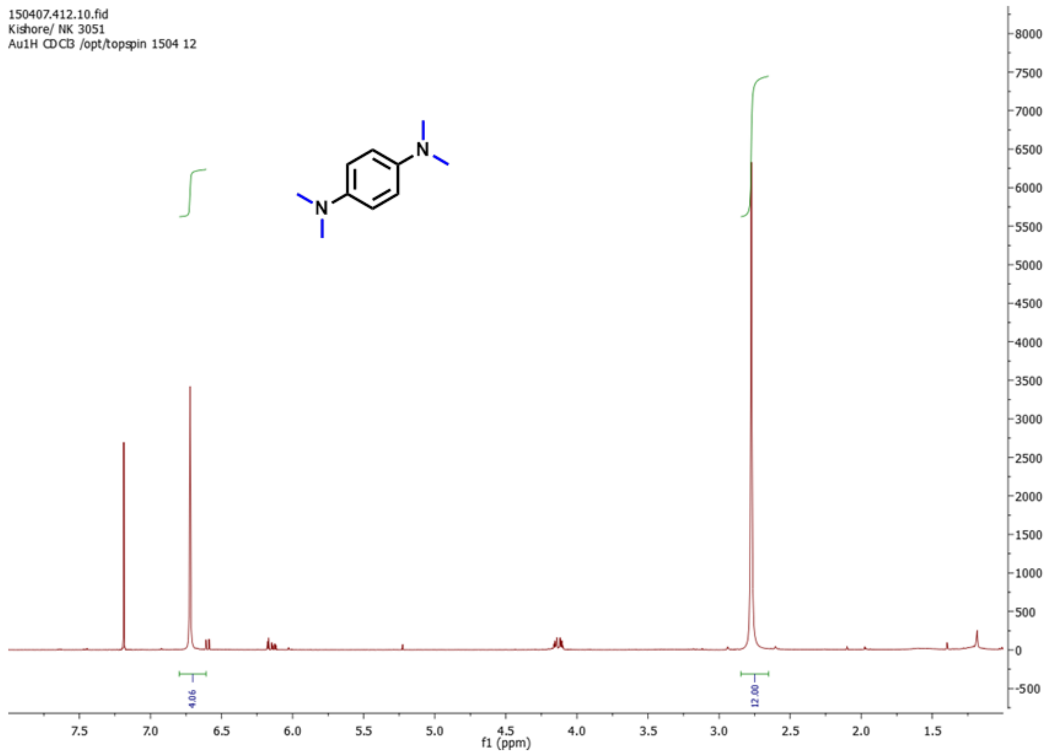

Supplementary Figure 14. ^{1}H NMR of *N,N,N′,N′*-Tetramethyl-*p*-phenylenediamine

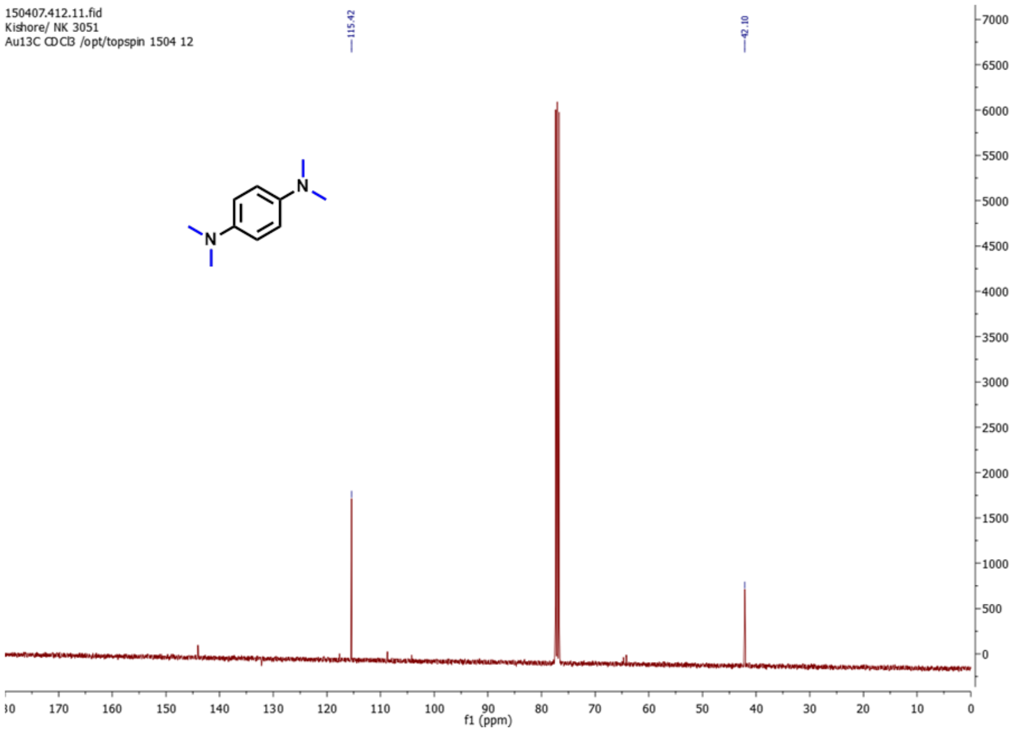

Supplementary Figure 15. ^{13}C NMR of *N,N,N′,N′*-Tetramethyl-*p*-phenylenediamine

Qualitative Compound Report

**Instrument Name**	ESI-TOF/MS	**Date Filename**	D:\MassHunter\Data\1505\15051810.d
**Acq Method**	HRMS Pos oS.m	**Sample Name**	NK 3051
**DA Method**	HRMS.m	**Position**	Vial 58
**User Name**	Fischer	**Comment**	MeOH/0,1% HCOOH in H2O 90:10

Compound Table

Name	RT	Abund	Formula	Ion Mass	Ionization Mode
1	0.178	1915466	C10 H16 N2	164.1313	Positive

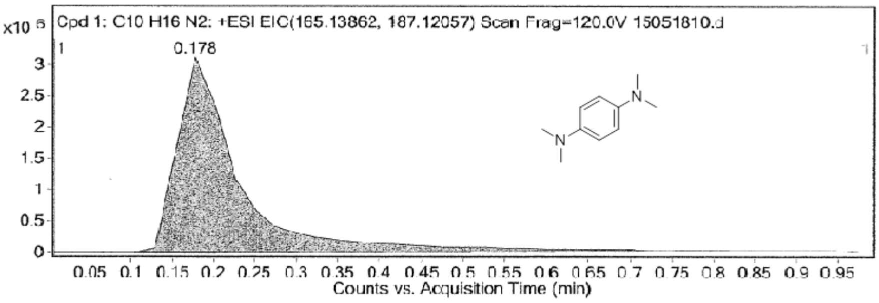

MS Zoomed Spectrum

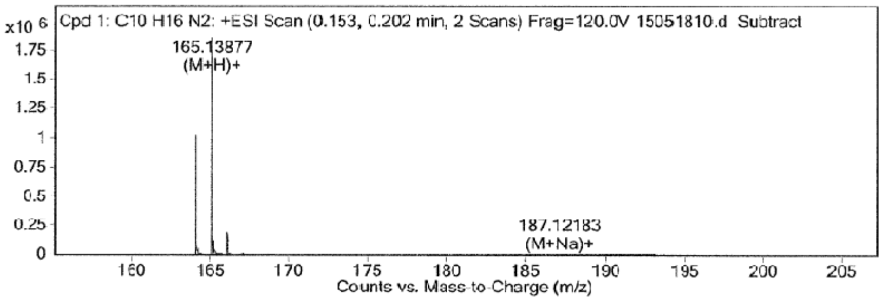

MS Spectrum Peak List

Ion	Abund	Formula	Calculated Mass	Measured Mass	Difference	Diff (ppm)
(M+H)+	1915465.75	C10H16N2	165.13862	165.13877	-0.14	-0.87

--- End Of Report ---

Supplementary Figure 16. HRMS (High Resolution Mass Spectroscopy) of *N,N,N′,N′*-Tetramethyl-*p*-phenylenediamine

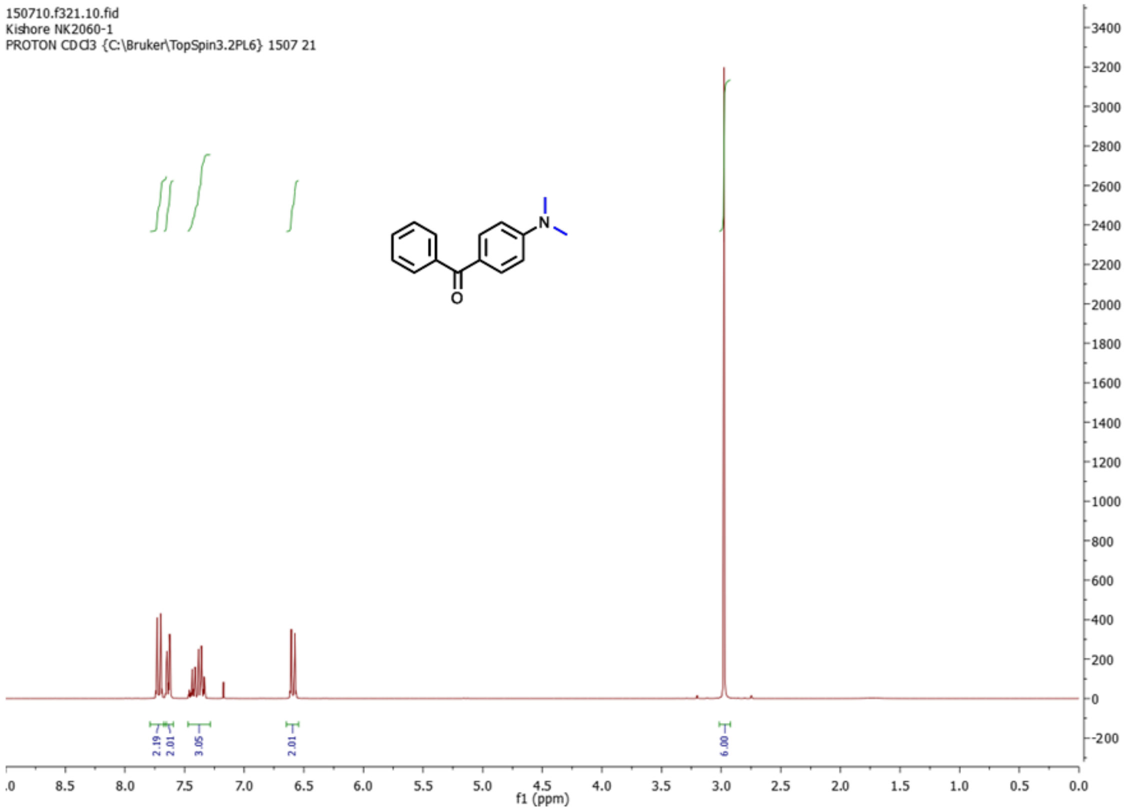

Supplementary Figure 17. ^{1}H NMR of (4-(Dimethylamino)phenyl)(phenyl)methanone

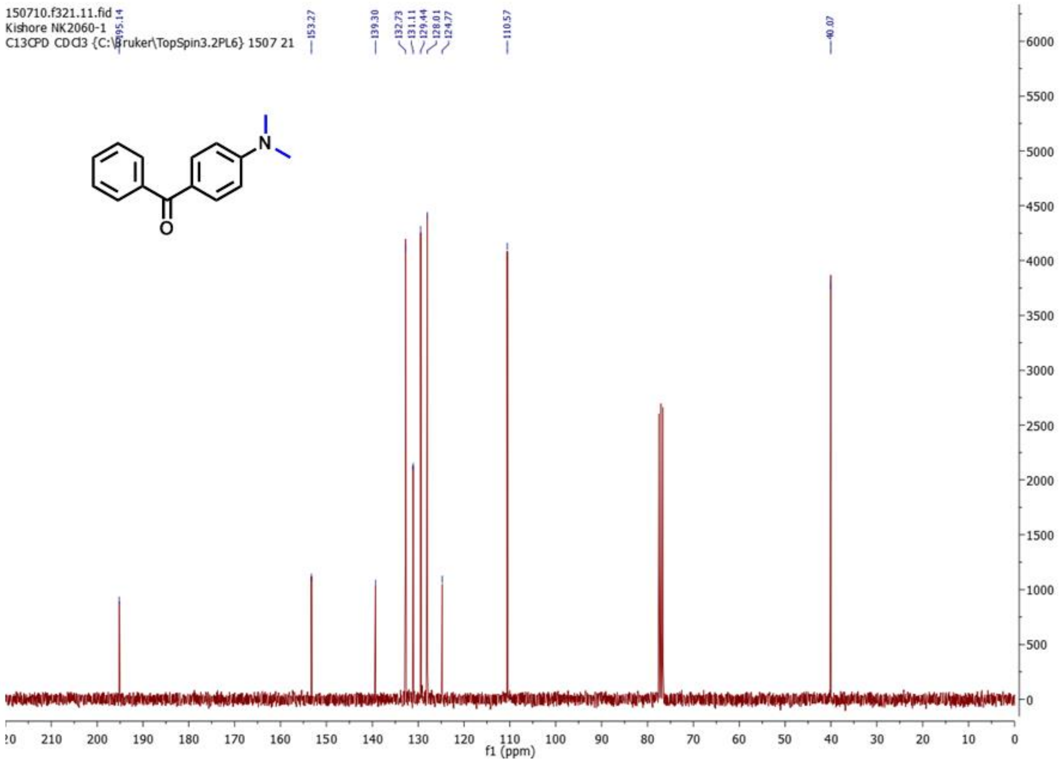

Supplementary Figure 18. ^{13}C NMR of (4-(Dimethylamino)phenyl)(phenyl)methanone

Qualitative Compound Report

**Instrument Name**	ESI TOF/MS	**Date Filename**	D:\MassHunter\Data\1505\15052010.d
**Acq Method**	HRMS Pos oS.m	**Sample Name**	NK2060-1
**DA Method**	HRMS.m	**Position**	Vial 71
**User Name**	Fischer	**Comment**	MeOH/0,1%HCOOH in H2O 90:10

Compound Table

Name	RT	Abund	Formula	Ion Mass	Ionization Mode
1	0.188	269108	C15 H15 N O	225.1154	Positive

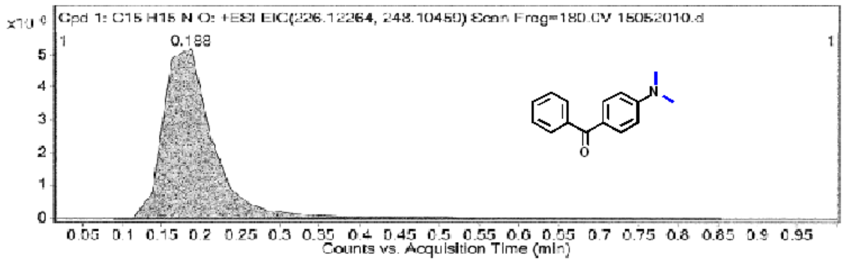

MS Zoomed Spectrum

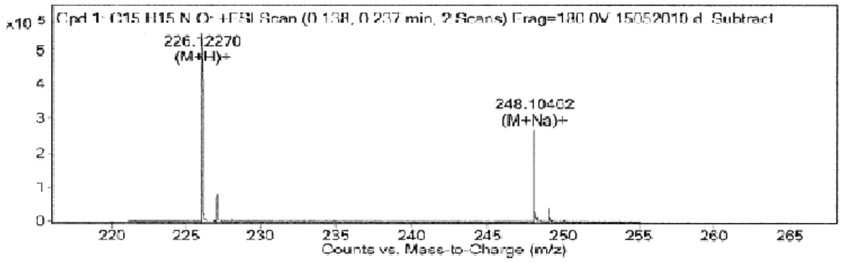

MS Spectrum Peak List

Ion	Abund	Formula	Calculated Mass	Measured Mass	Difference	Diff (ppm)
(M+H)+	562384	C15H15NO	226.12264	226.1227	-0.06	-0.27
(M+Na)+	269108.41	C15H15NO	248.10459	248.10462	-0.03	-0.13

--- End Of Report ---

Supplementary Figure 19. HRMS (High Resolution Mass Spectroscopy) of (4-(Dimethylamino)phenyl)(phenyl)methanone

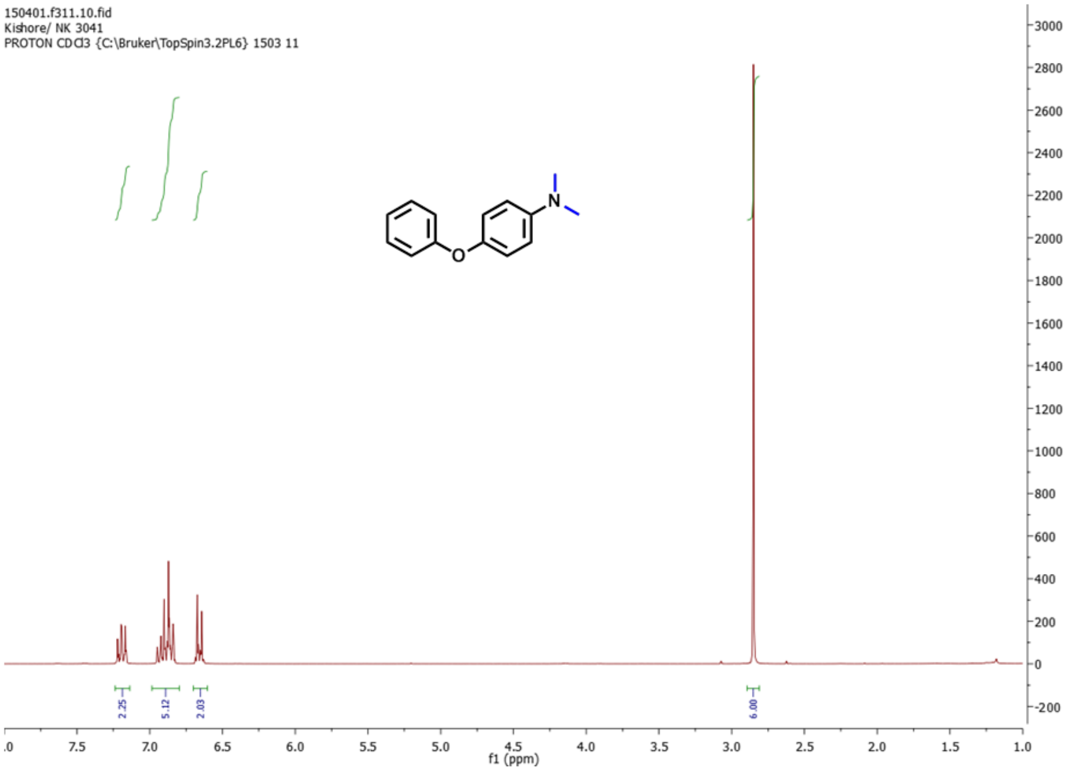

Supplementary Figure 20. ^{1}H NMR of *N*,*N*-Dimethyl-4-phenoxyaniline

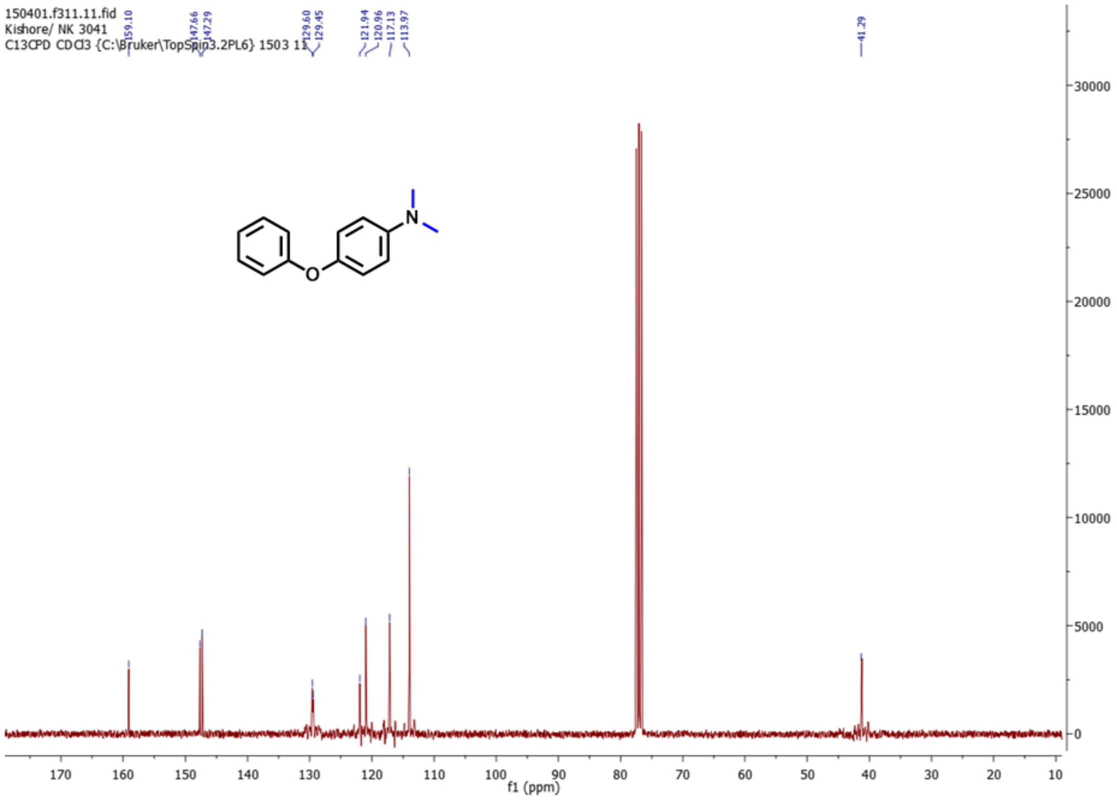

Supplementary Figure 21. ^{13}C NMR of *N*,*N*-Dimethyl-4-phenoxyaniline

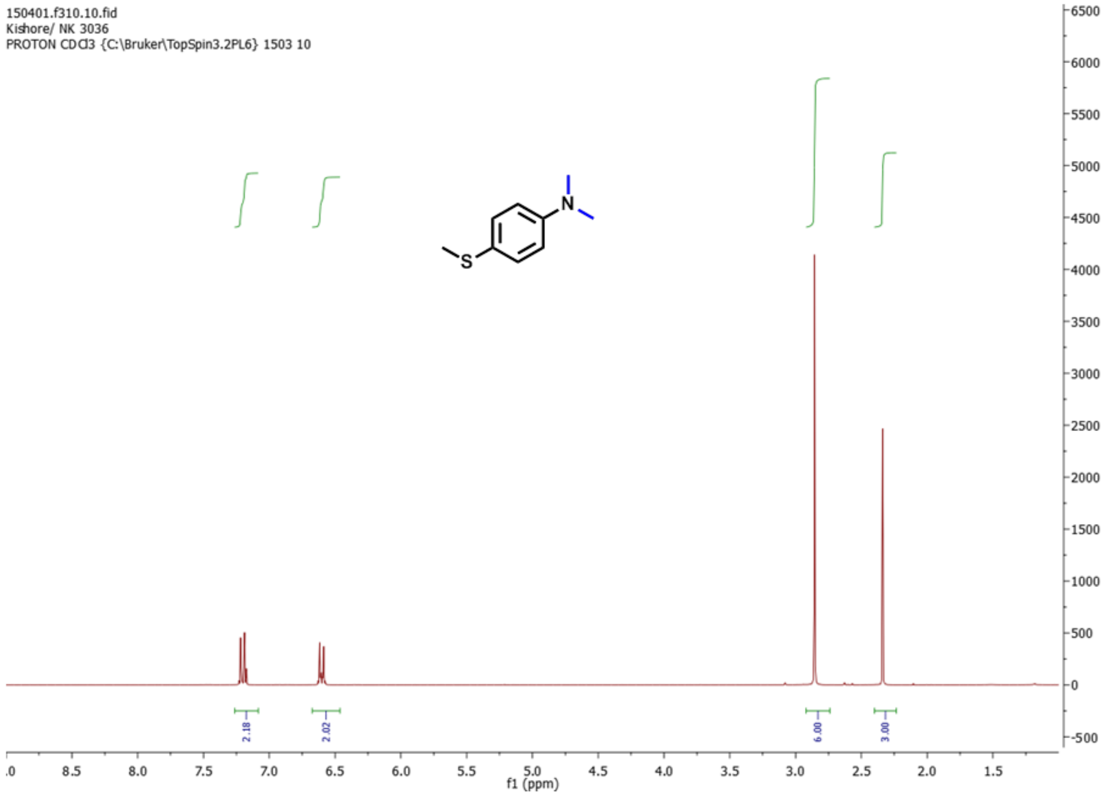

Supplementary Figure 22. ^{1}H NMR of *N*,*N*-Dimethyl-4-(methylthio)aniline

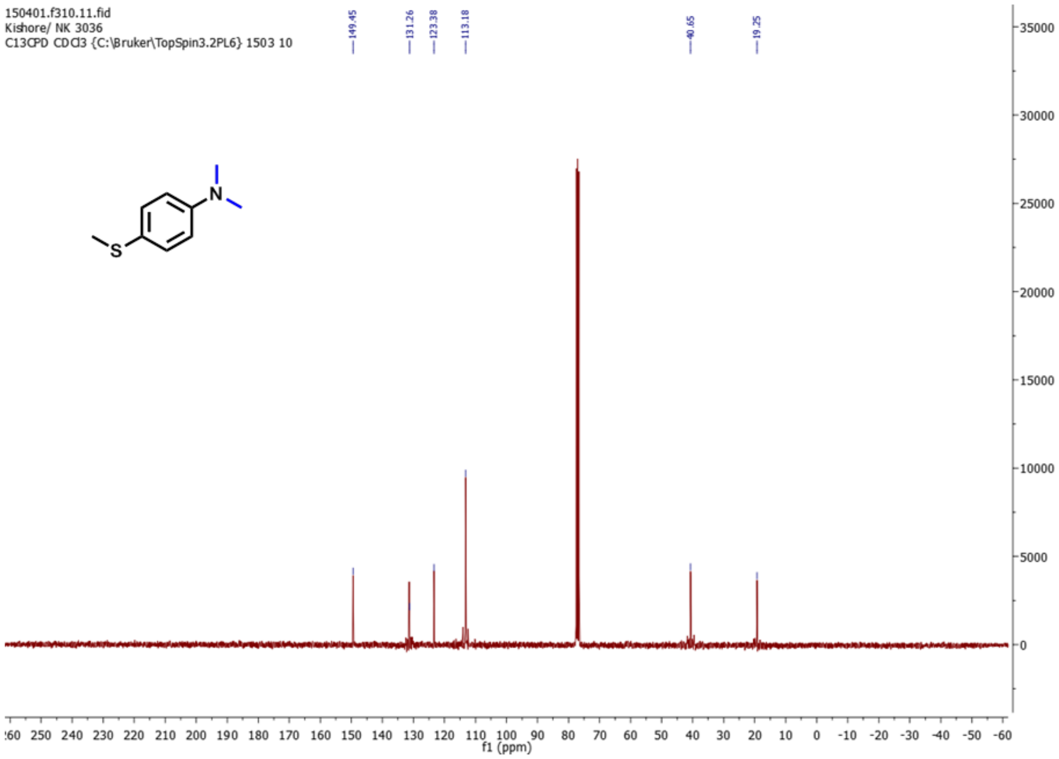

Supplementary Figure 23. ^{13}C NMR of *N*,*N*-Dimethyl-4-(methylthio)aniline

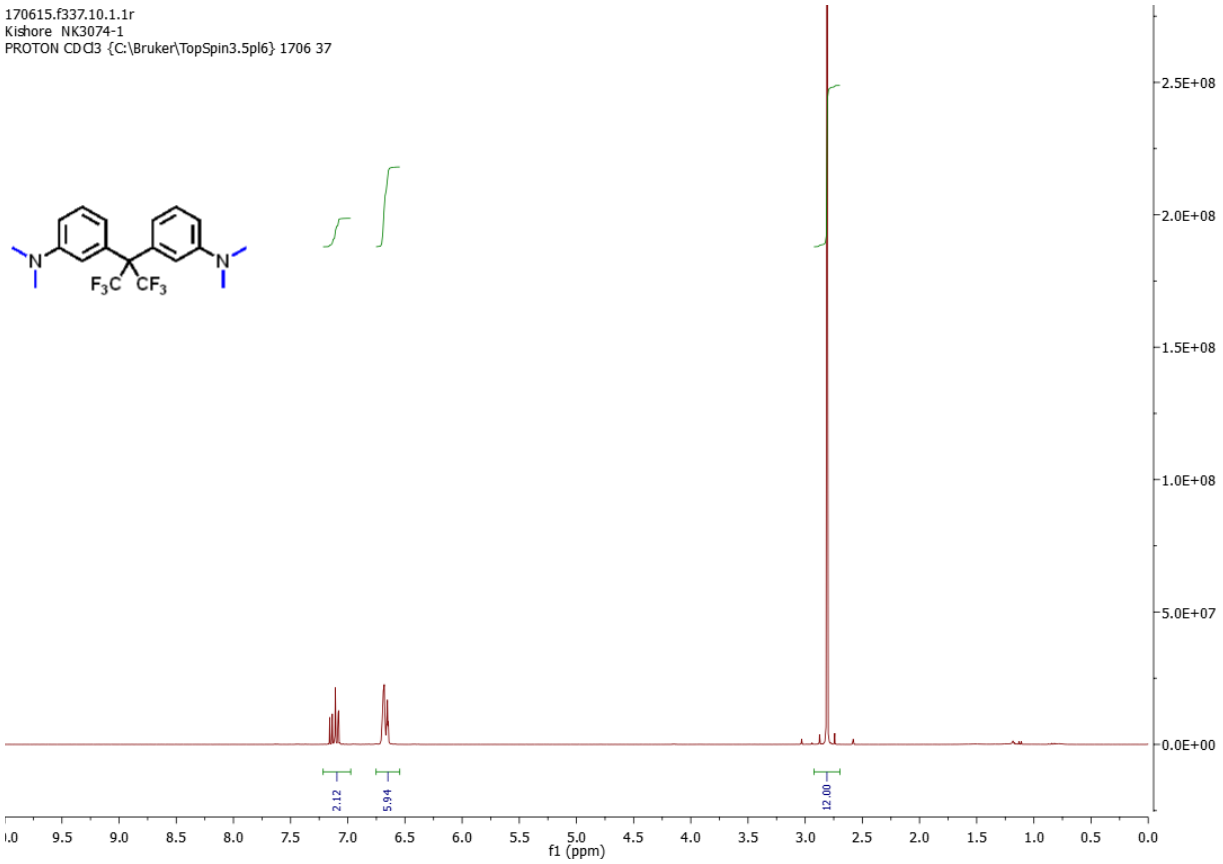

Supplementary Figure 24. ^{1}H NMR of 3,3'-(perfluoropropane-2,2-diyl)bis(N,N-dimethylaniline)

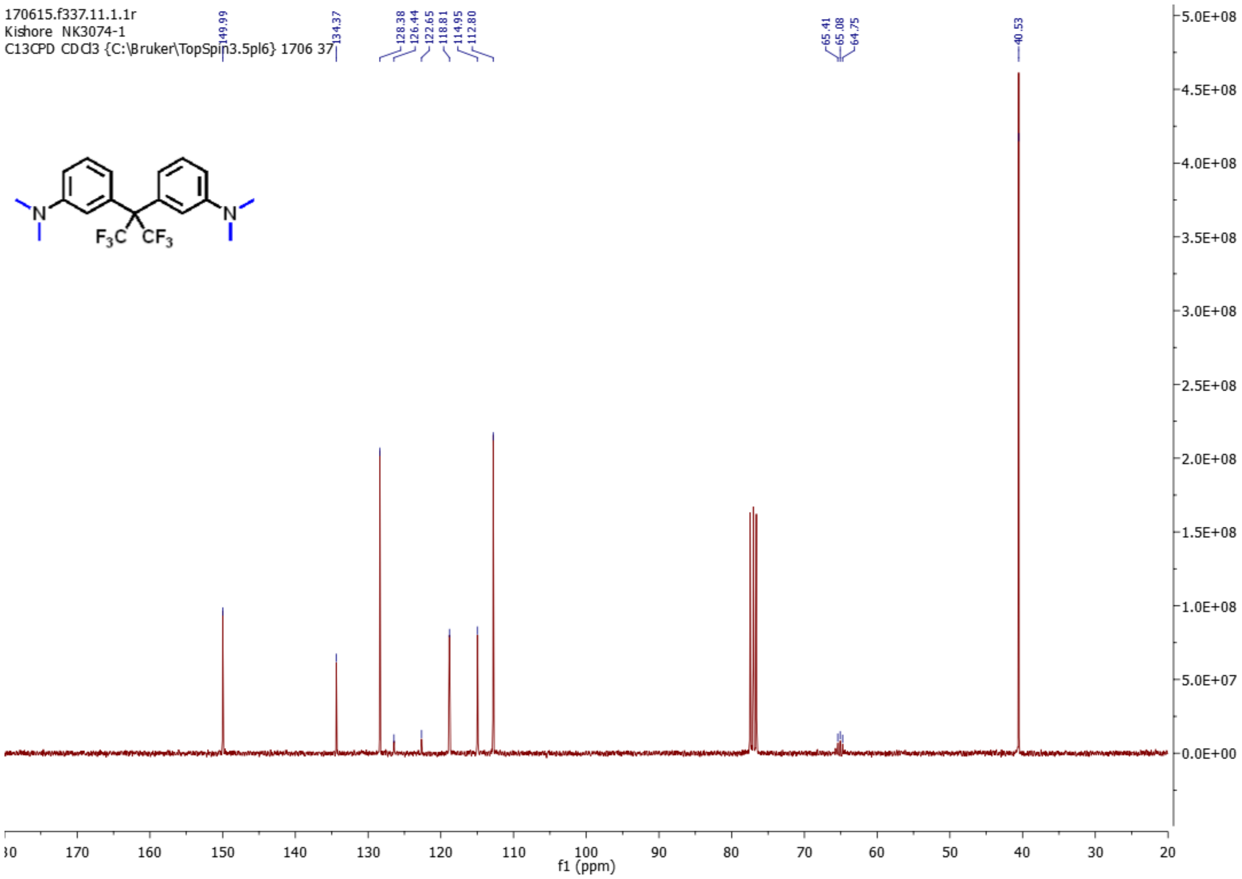

Supplementary Figure 25. ^{13}C NMR of 3,3'-(perfluoropropane-2,2-diyl)bis(N,N-dimethylaniline)

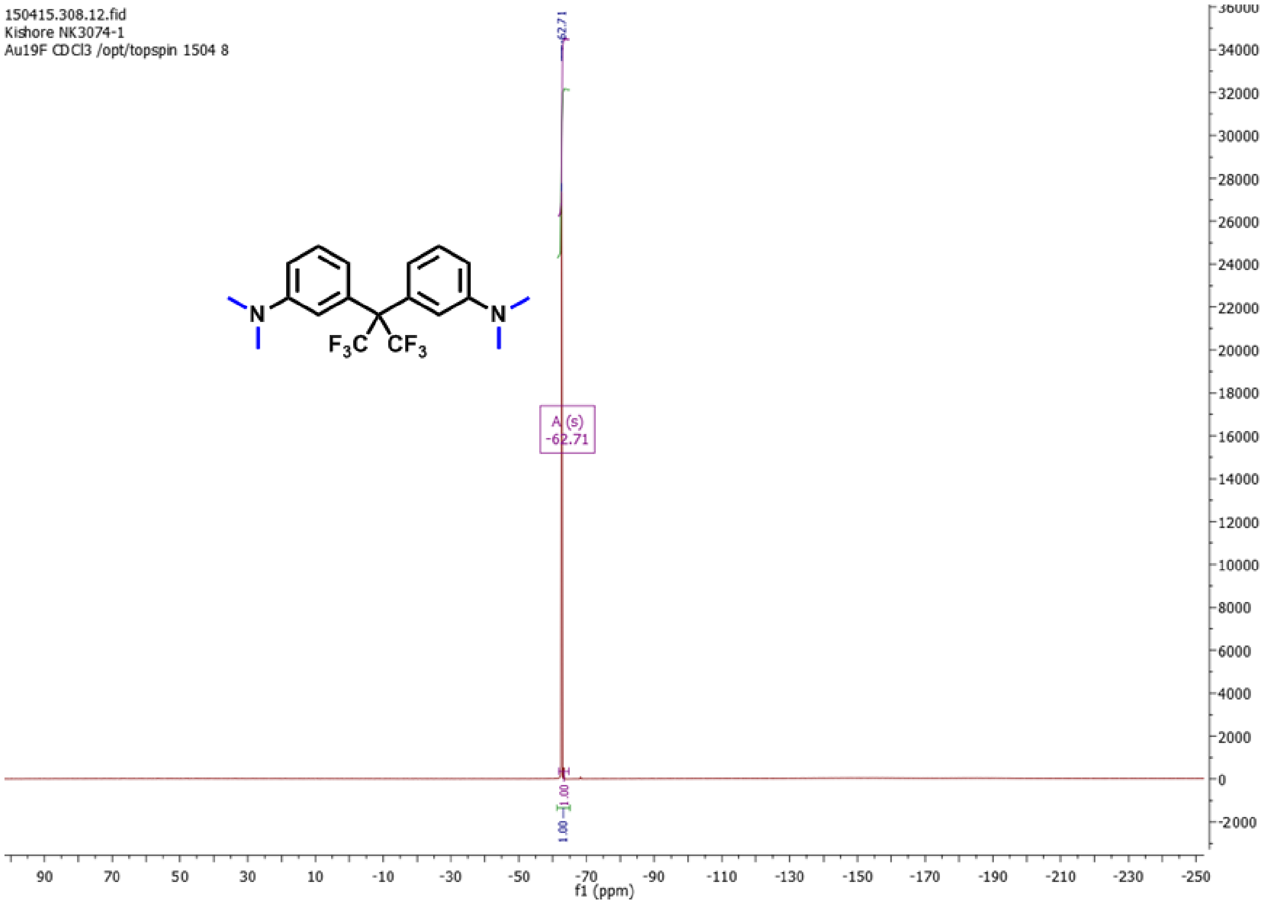

Supplementary Figure 26. ^{19}F NMR of 3,3'-(perfluoropropane-2,2-diyl)bis(N,N-dimethylaniline)

Qualitative Compound Report

**Instrument Name**	ESI-TOF/MS	**Date Filename**	D:\MassHunter\Data\1505\15051905.d
**Acq Method**	HRMS Pos oS.m	**Sample Name**	NK3074-1
**DA Method**	HRMS.m	**Position**	Vial 75
**User Name**	Fischer	**Comment**	MeOH/0,1%HCOOH in H2O 90:10

Compound Table

Name	RT	Abund	Formula	Ion Mass	Ionization Mode
1	0.172	54906	C19 H20 F6 N2	390.1531	Positive

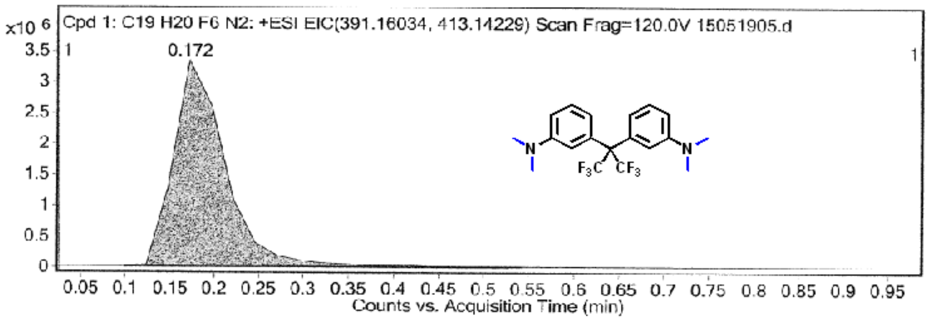

MS Zoomed Spectrum

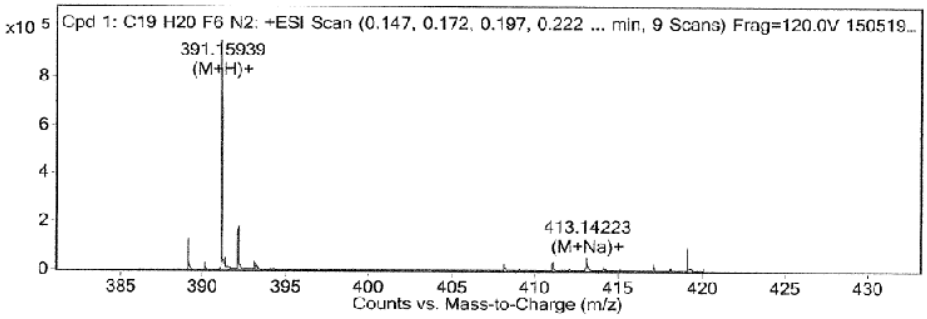

MS Spectrum Peak List

Ion	Abund	Formula	Calculated Mass	Measured Mass	Difference	Diff (ppm)
(M+H)+	950353.63	C19H20F6N2	391.16034	391.15939	0.96	2.45
(M+Na)+	54906.38	C19H20F6N2	413.14229	413.14223	0.06	0.15

--- End Of Report ---

Supplementary Figure 27. HRMS (High Resolution Mass Spectroscopy) of 3,3'-(perfluoropropane-2,2-diyl)bis(N,N-dimethylaniline)

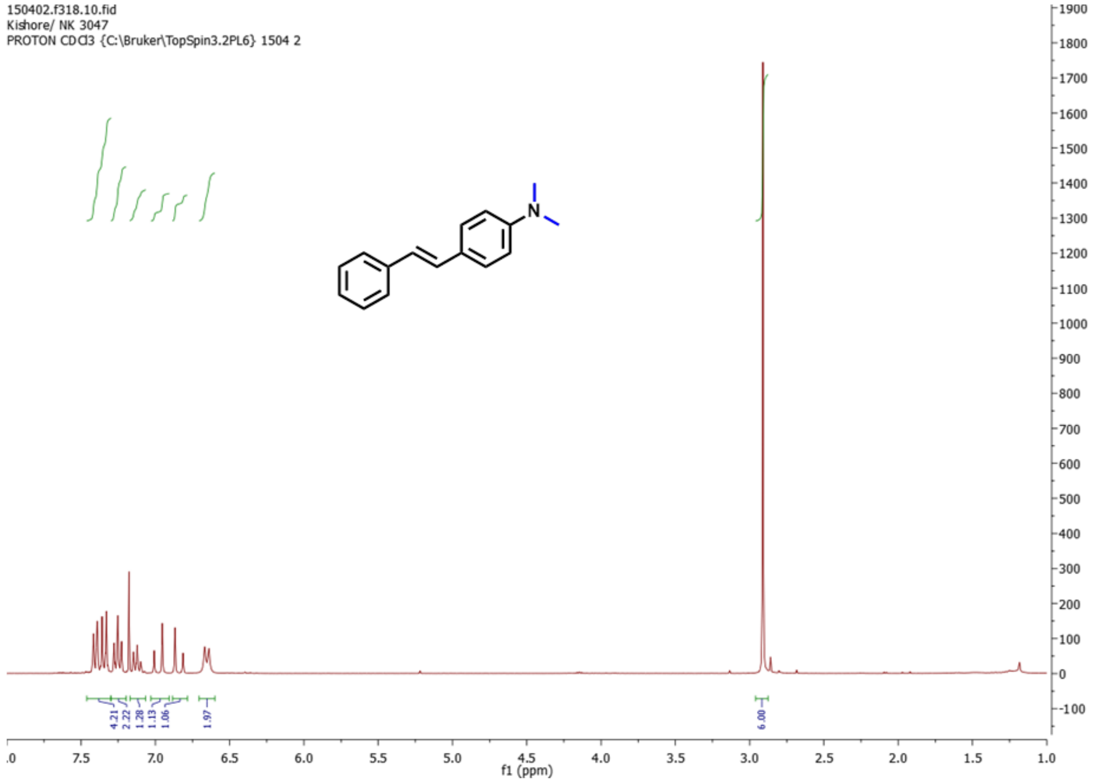

Supplementary Figure 28. ^{1}H NMR of (E)-*N*,*N*-Dimethyl-4-styrylaniline

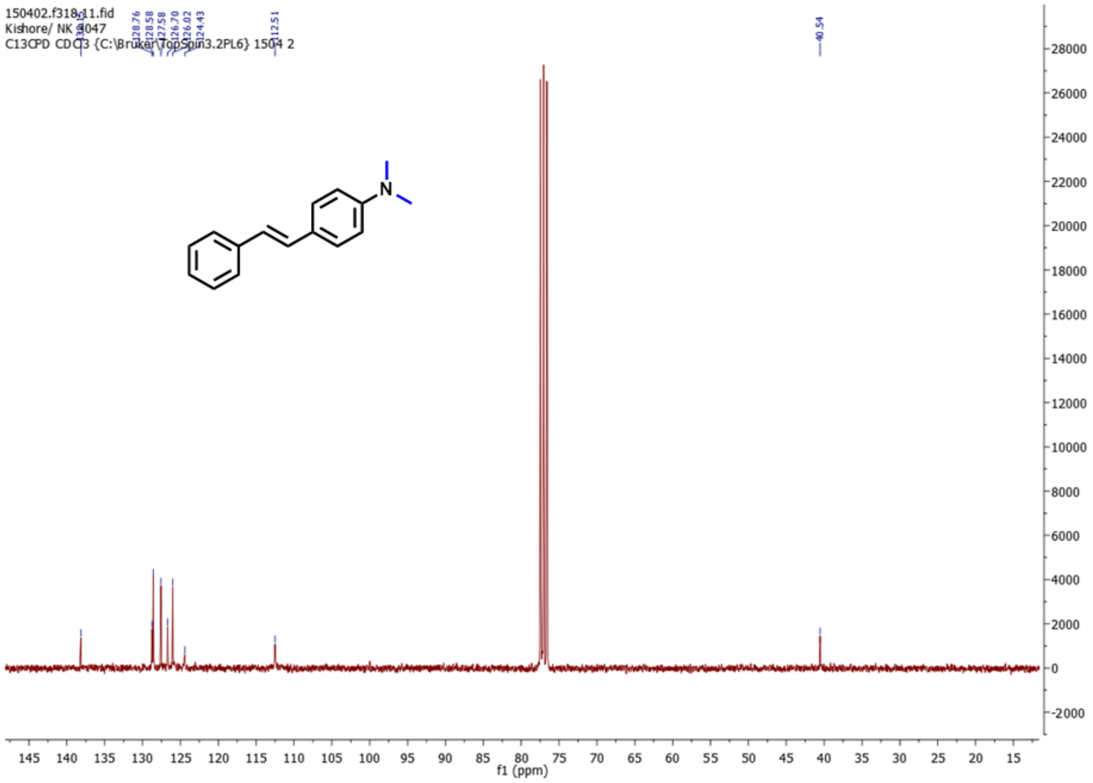

Supplementary Figure 29. ^{13}C NMR of (E)-*N*,*N*-Dimethyl-4-styrylaniline

Qualitative Compound Report

**Instrument Name**	ESI-TOF/MS	**Date Filename**	D:\MassHunter\Data\1505\15051807.d
**Acq Method**	HRMS Pos oS.m	**Sample Name**	NK 3047
**DA Method**	HRMS.m	**Position**	Vial 57
**User Name**	Fischer	**Comment**	MeOH/0,1%HCOOH in H2O 90:10

Compound Table

Name	RT	Abund	Formula	Ion Mass	Ionization Mode
1	0.186	285138	C16 H17 N	223.1361	Positive

MS Zoomed Spectrum

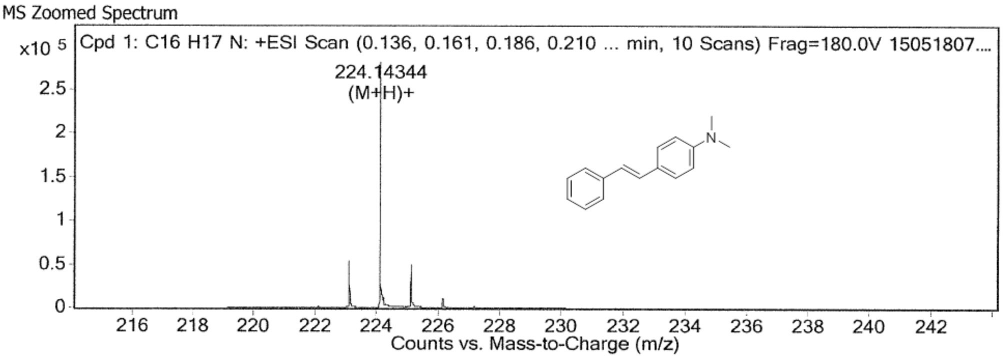

MS Spectrum Peak List

Ion	Abund	Formula	Calculated Mass	Measured Mass	Difference	Diff (ppm)
(M+H)+	285138.09	C16H17N	224.14338	224.14344	-0.07	-0.3

--- End Of Report ---

Supplementary Figure 30. HRMS (High Resolution Mass Spectroscopy) of (E)-*N*,*N*-Dimethyl-4-styrylaniline

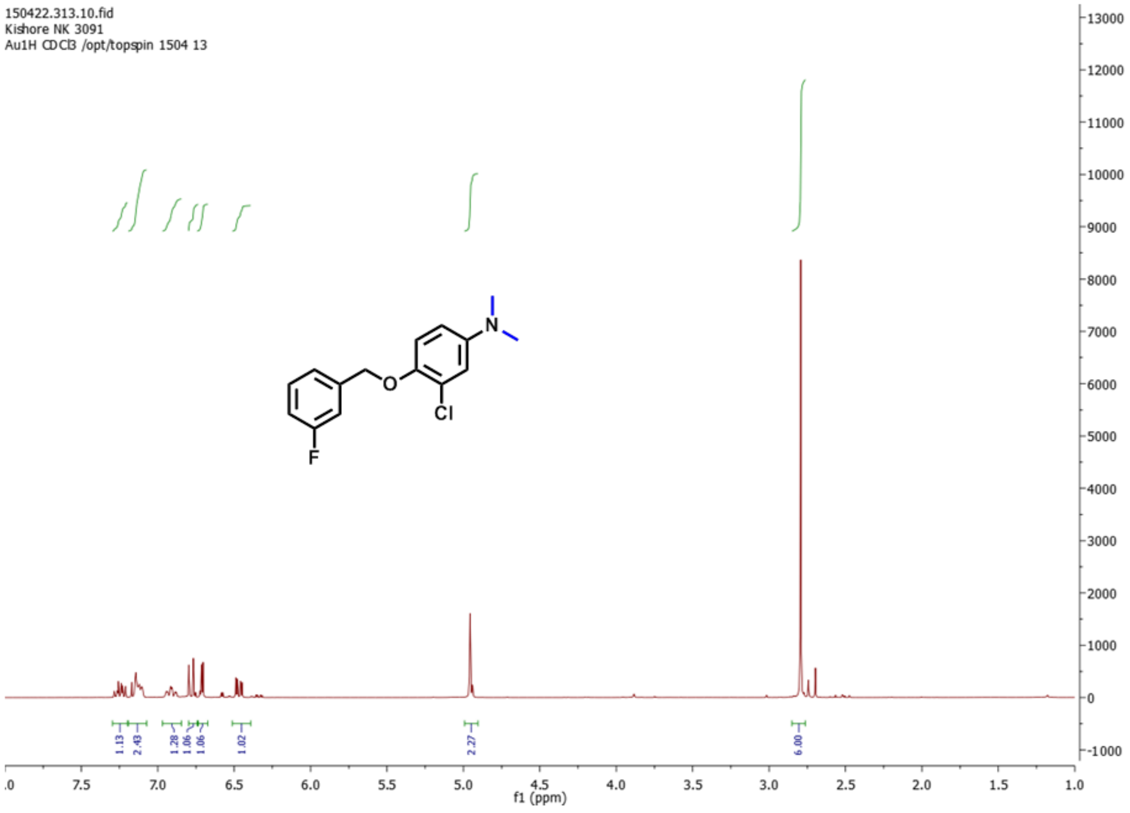

Supplementary Figure 31. ^{1}H NMR of 3-Chloro-4-((3-fluorobenzyl)oxy)-*N*,*N*-dimethylaniline

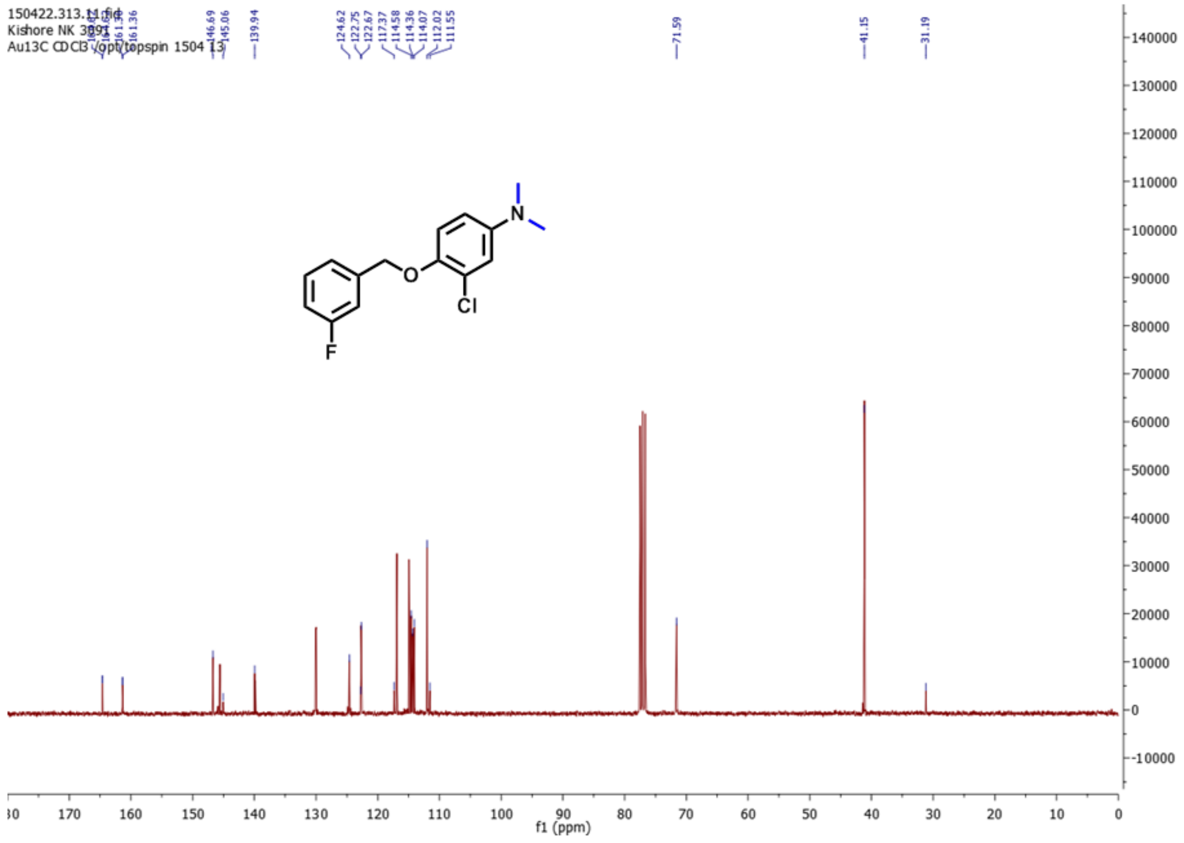

Supplementary Figure 32. ^{13}C NMR of 3-Chloro-4-((3-fluorobenzyl)oxy)-*N*,*N*-dimethylaniline

Qualitative Compound Report

**Instrument Name**	ESI-TOF/MS	**Date Filename**	D:\MassHunter\Data\1505\15051910.d
**Acc Method**	HRMS Neg nS.m	**Sample Name**	NK3091
**DA Method**	HRMS.m	**Position**	Vial 77
**User Name**	Fischer	**Comment**	MeOH/0.1%HCOOH in H2O 90:10

Compound Table

Name	RT	Abund	Formula	Ion Mass	Ionization Mode
1	0.166	181200	C15 H15 Cl F N O	279.0826	Negative

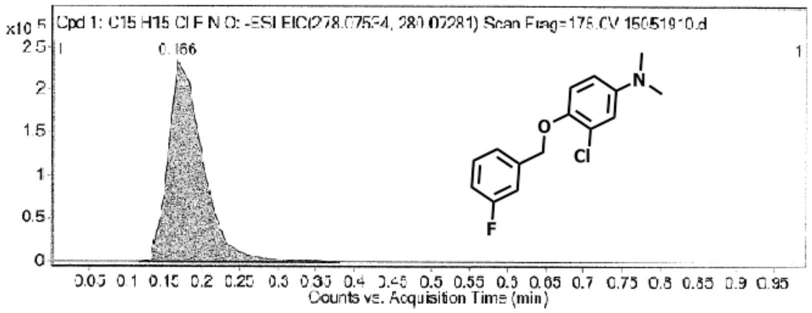

MS Zoomed Spectrum

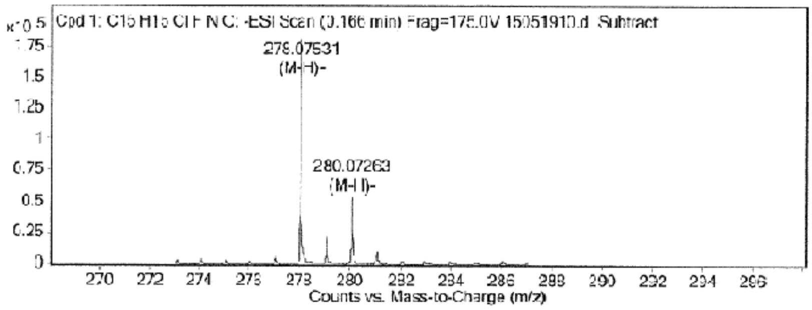

MS Spectrum Peak List

Ion	Abund	Formula	Calculated Mass	Measured Mass	Difference	Diff (ppm)
(M-H)-	181200.33	C15H15ClFNO	278.07534	278.07531	0.04	0.14
(M-H)-	54184.1	C15H15ClFNO	280.07281	280.07263	0.18	0.64

--- End Of Report ---

Supplementary Figure 33. HRMS (High Resolution Mass Spectroscopy) of 3-Chloro-4-((3-fluorobenzyl)oxy)-*N*,*N*-dimethylaniline

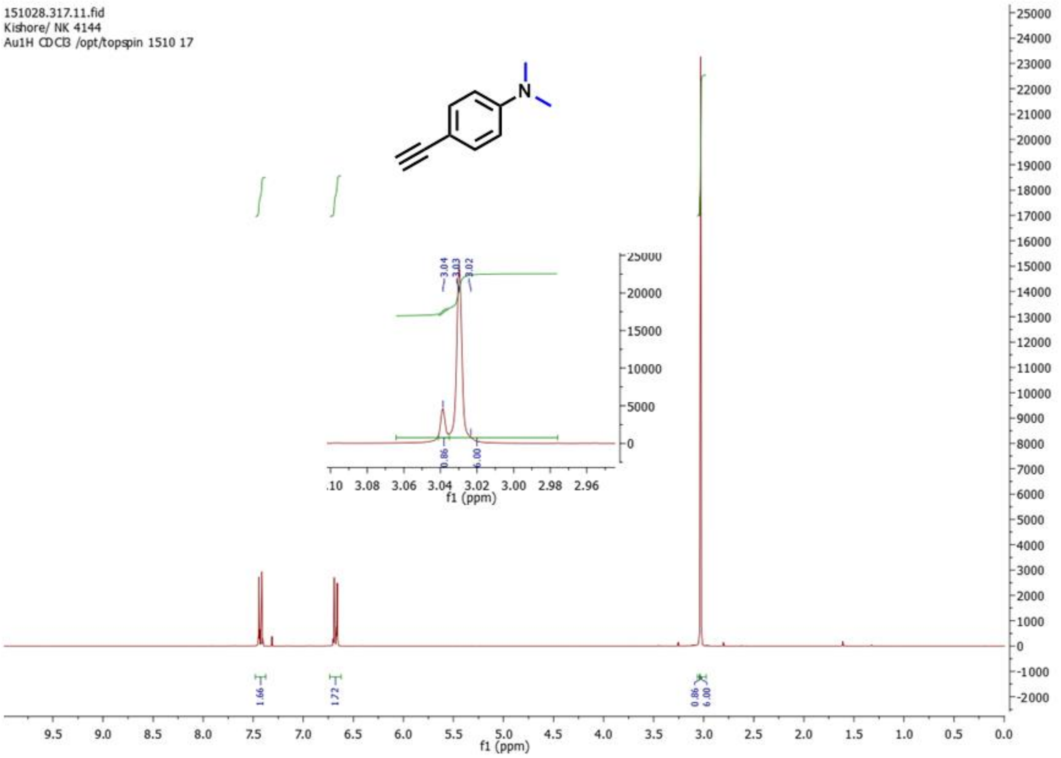

Supplementary Figure 34. ^{1}H NMR of 4-Ethynyl-*N*,*N*-dimethylaniline

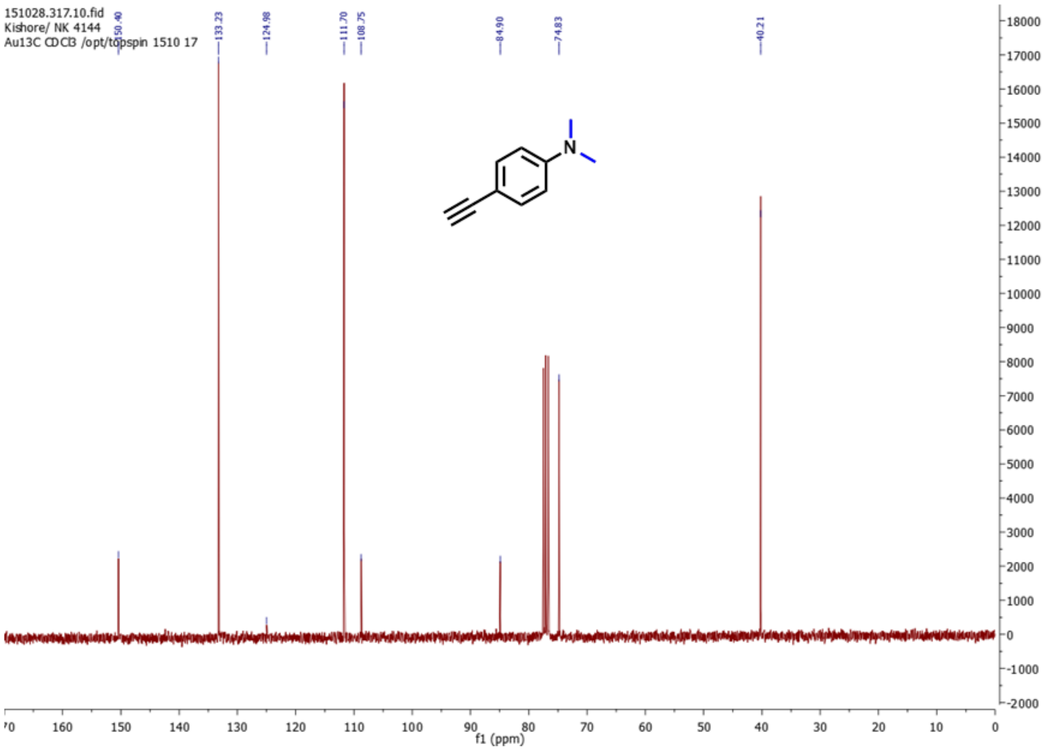

Supplementary Figure 35. ^{13}C NMR of 4-Ethynyl-*N*,*N*-dimethylaniline

Qualitative Compound Report

**Instrument Name**	ESI-TOF/MS	**Date Filename**	D:\MassHunter\Data\1511\15110909.d
**Acq Method**	HRMS Pos oS.m	**Sample Name**	NK4144
**DA Method**	HRMS.m	**Position**	Vial 72
**User Name**	Fischer	**Comment**	MeOH/0,1%HCOOH in H2O 90:10

Compound Table

Name	RT	Abund	Formula	Ion Mass	Ionization Mode
1	0.177	862283	C10 H11 N	145.08915	Positive

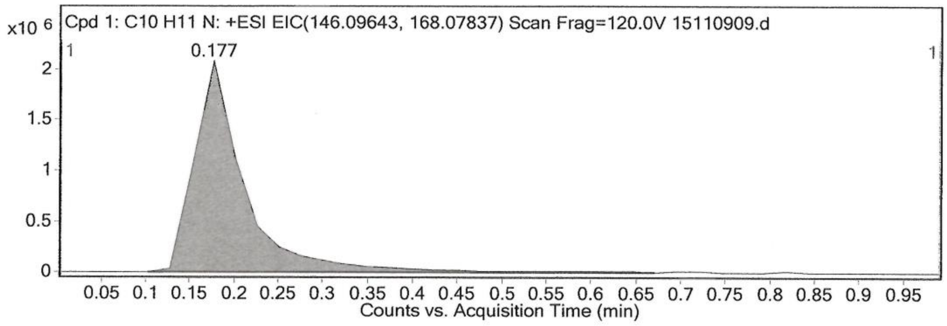

MS Zoomed Spectrum

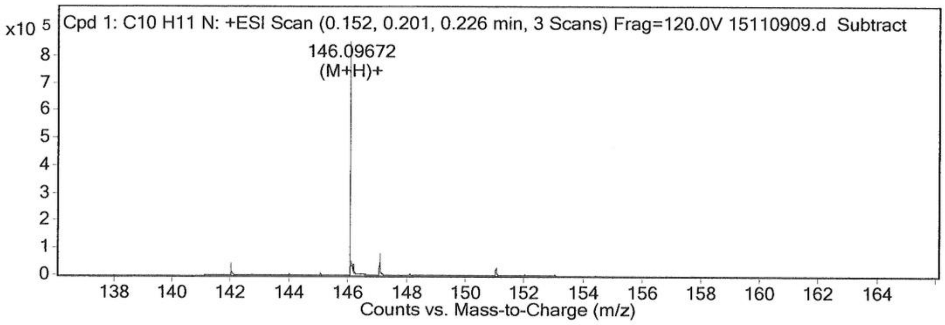

MS Spectrum Peak List

Ion	Abund	Formula	Calculated Mass	Measured Mass	Difference	Diff (ppm)
(M+H)+	862282.75	C10H11N	146.09643	146.09672	-0.29	-2

--- End Of Report ---

Supplementary Figure 36. HRMS (High Resolution Mass Spectroscopy) of 4-Ethynyl-*N*,*N*-dimethylaniline

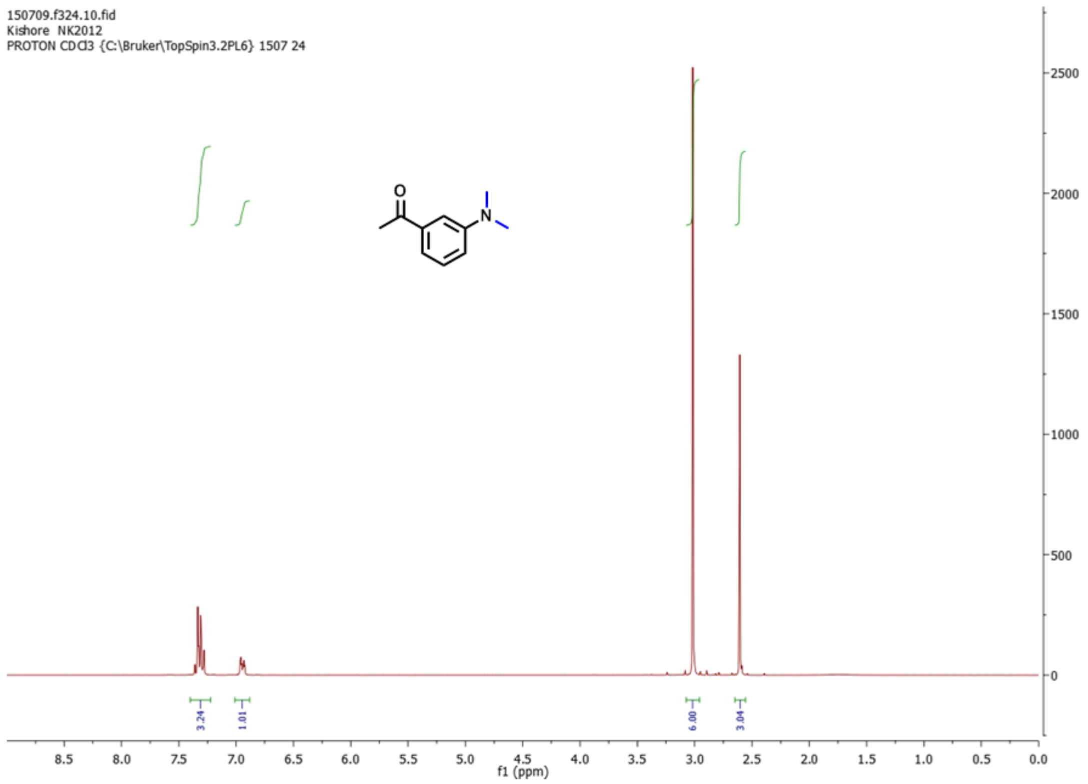

Supplementary Figure 37. ^{1}H NMR of 1-(3-(Dimethylamino)phenyl)ethan-1-one

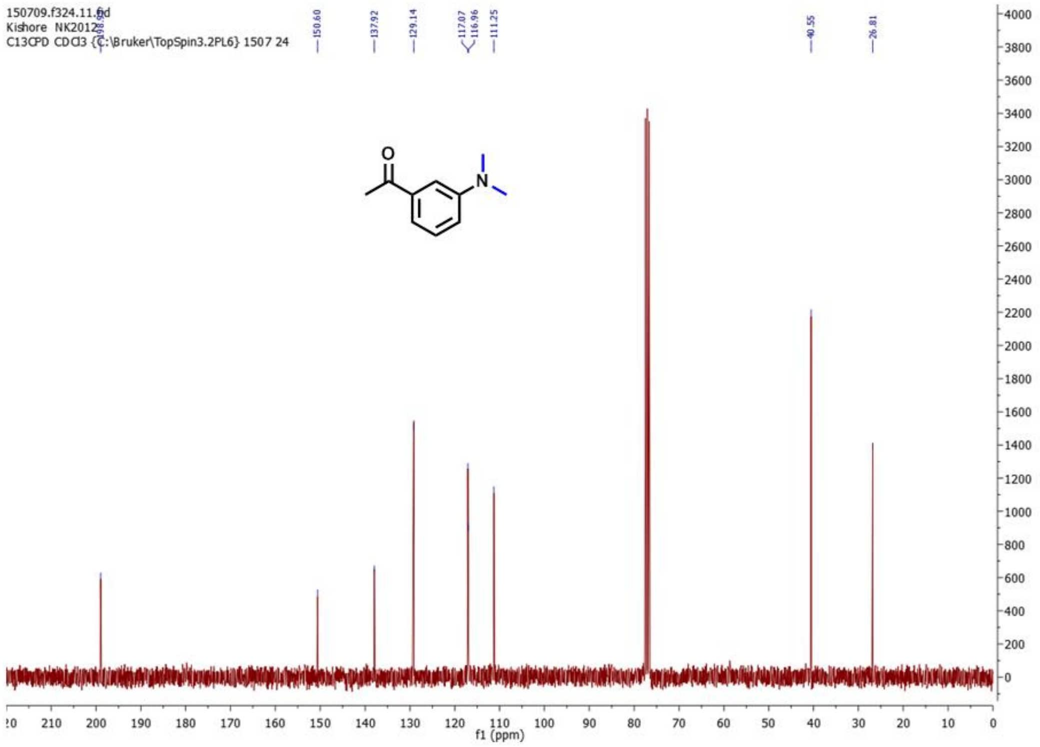

Supplementary Figure 38. ^{13}C NMR of 1-(3-(Dimethylamino)phenyl)ethan-1-one

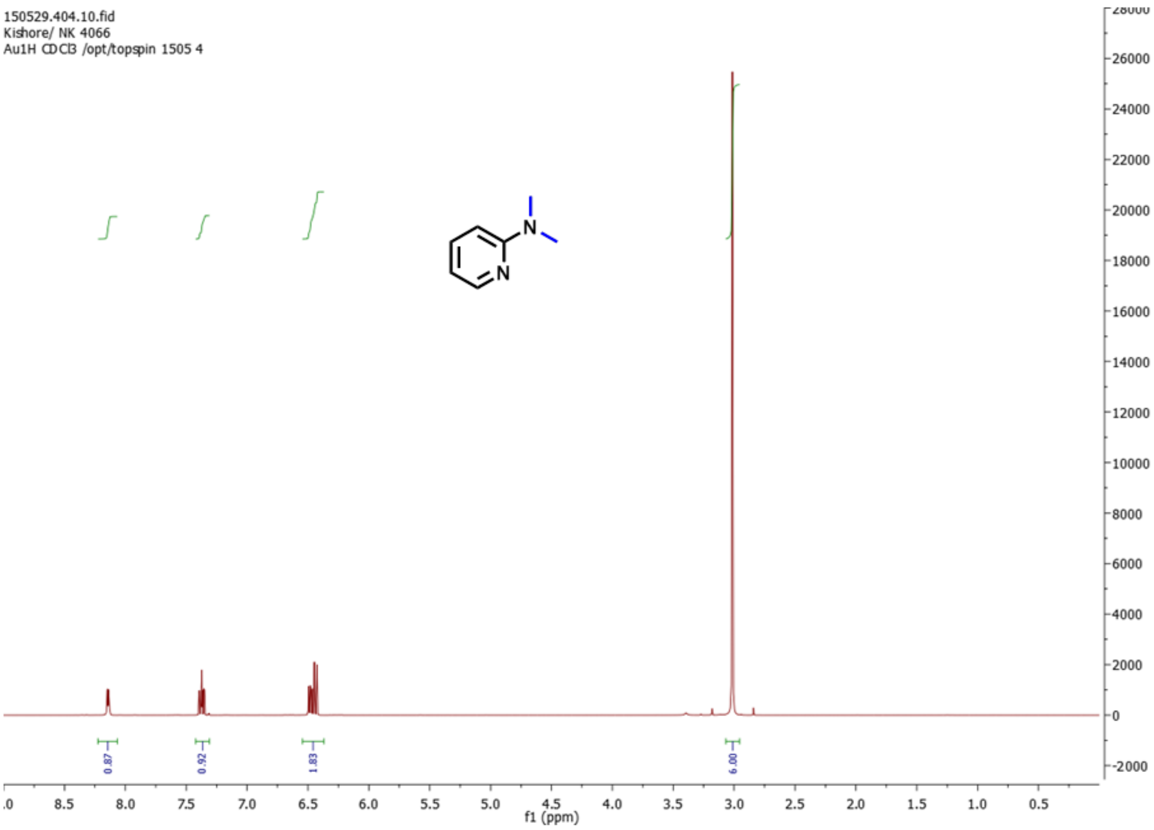

Supplementary Figure 39. ^{1}H NMR of *N*,*N*-Dimethylpyridin-2-amine

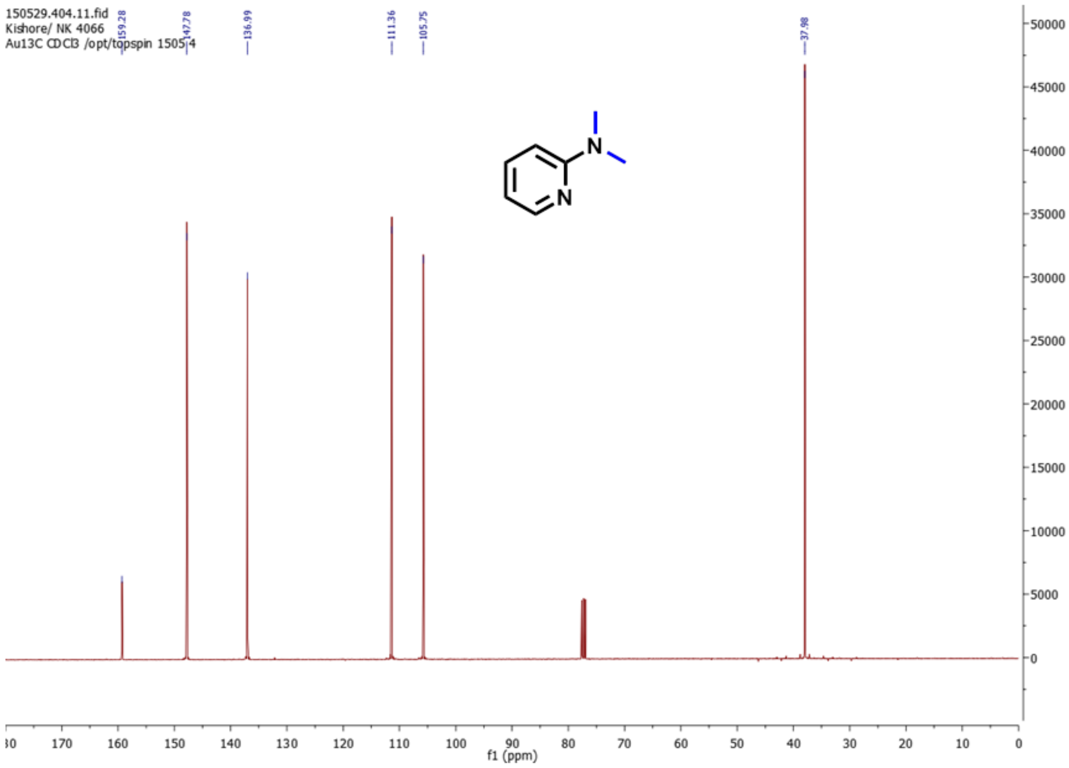

Supplementary Figure 40. ^{13}C NMR of *N*,*N*-Dimethylpyridin-2-amine

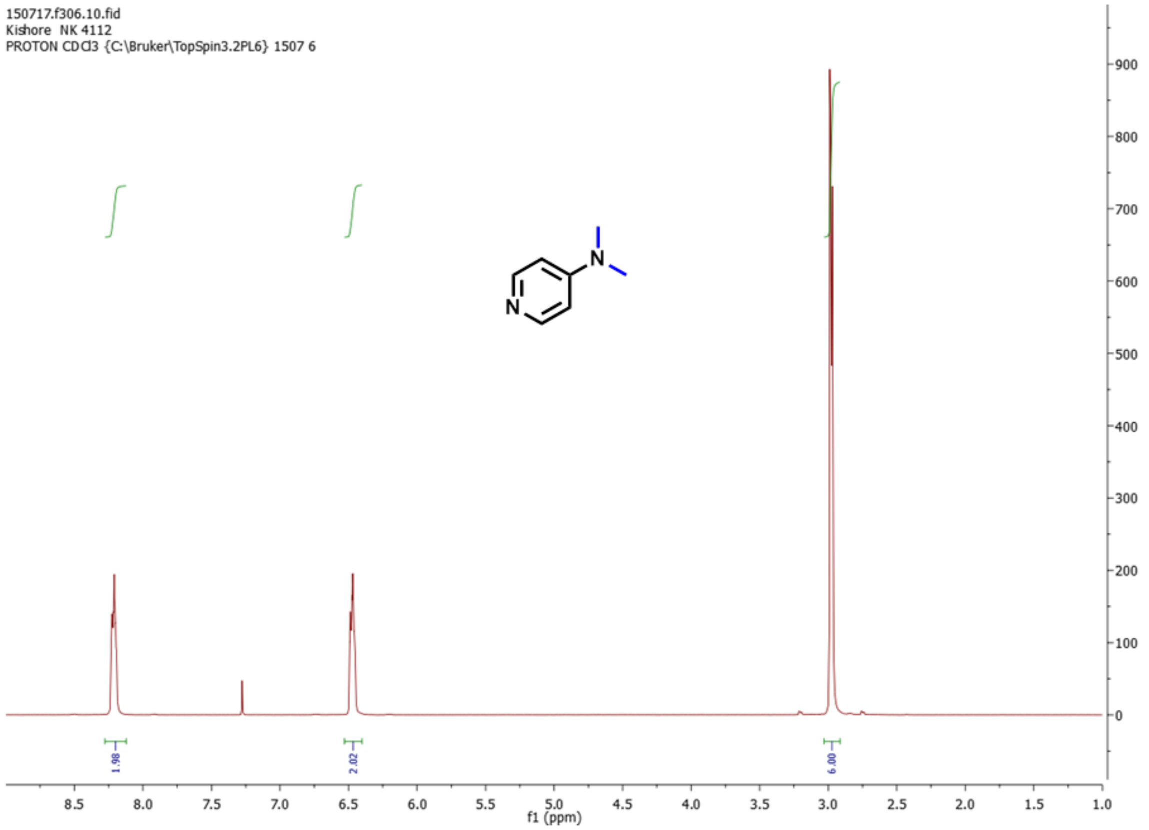

Supplementary Figure 41. ^{1}H NMR of *N*,*N*-Dimethylpyridin-4-amine

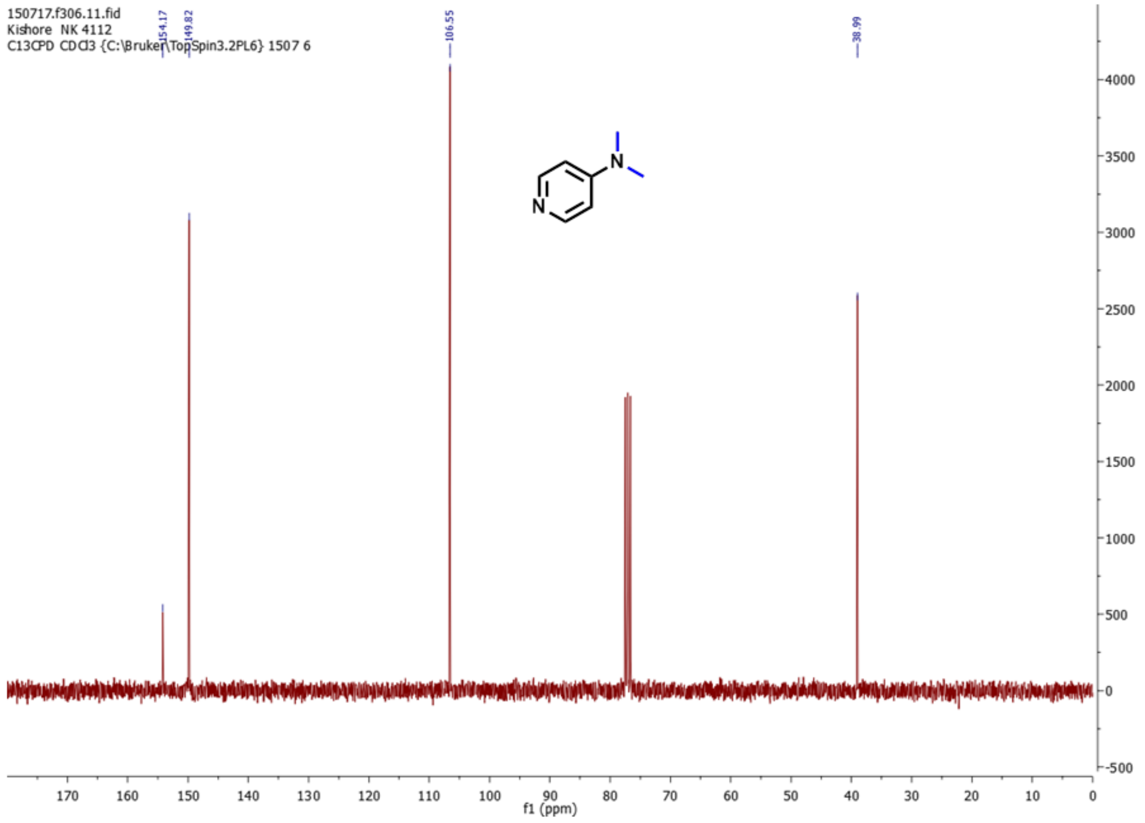

Supplementary Figure 42. ^{13}C NMR of *N*,*N*-Dimethylpyridin-4-amine

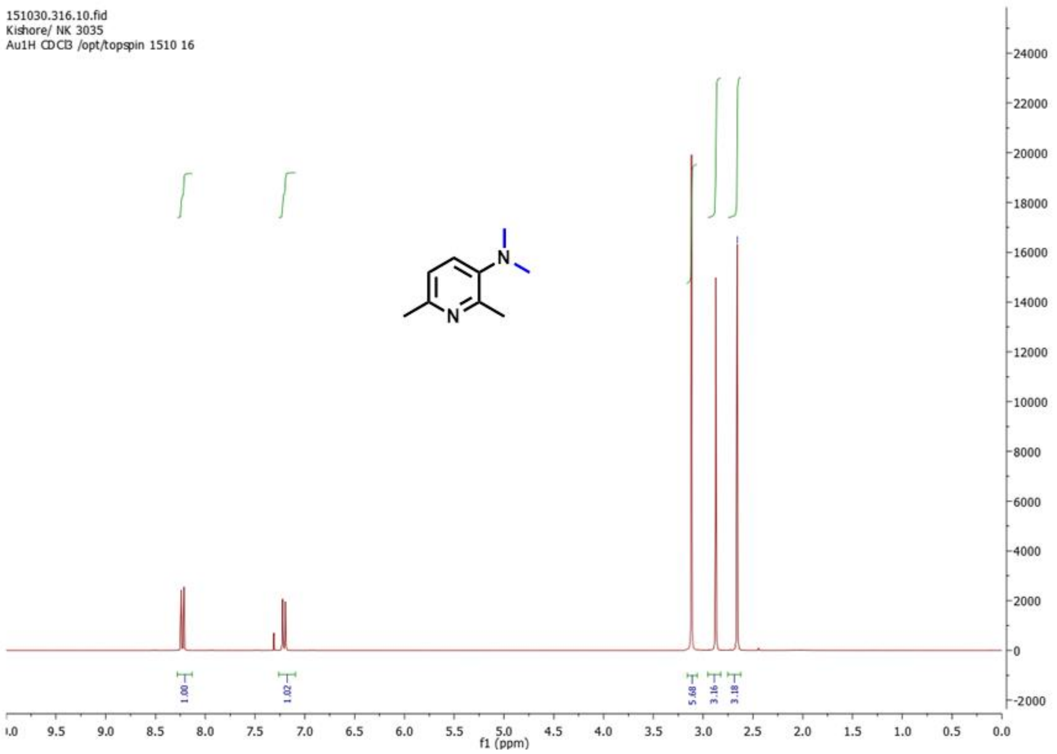

Supplementary Figure 43. ^{1}H NMR of *N*,*N*,2,6-Tetramethylpyridin-3-amine

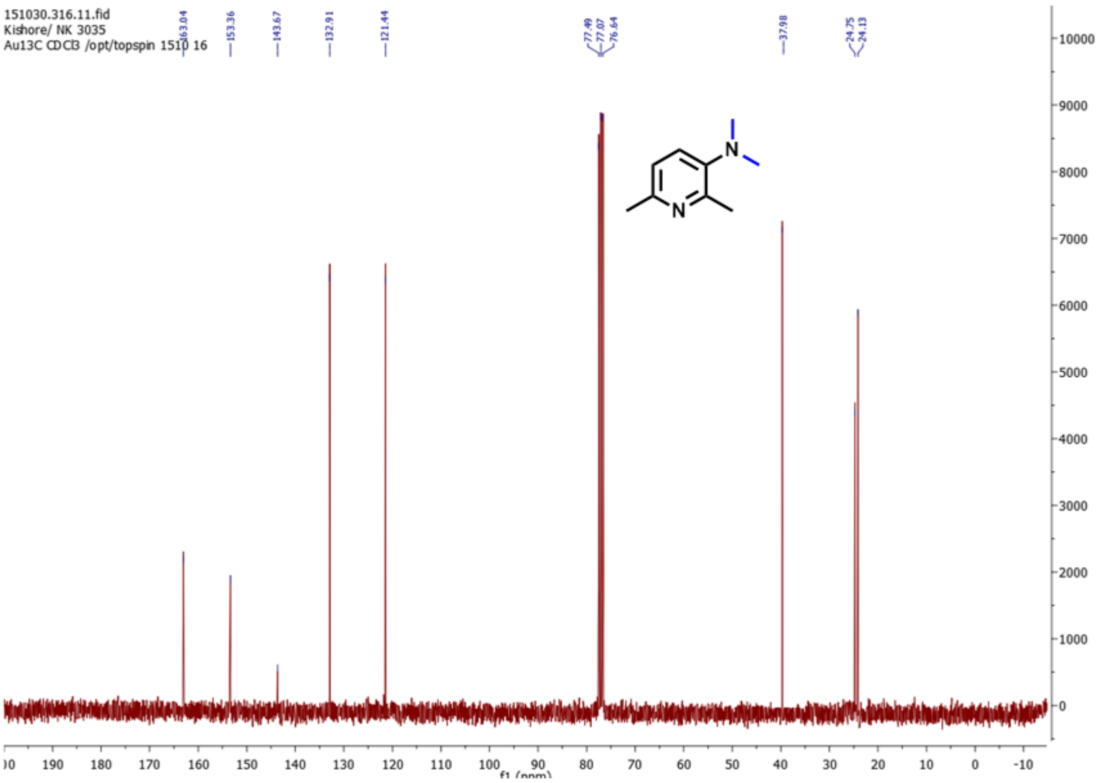

Supplementary Figure 44. ^{13}C NMR of *N*,*N*,2,6-Tetramethylpyridin-3-amine

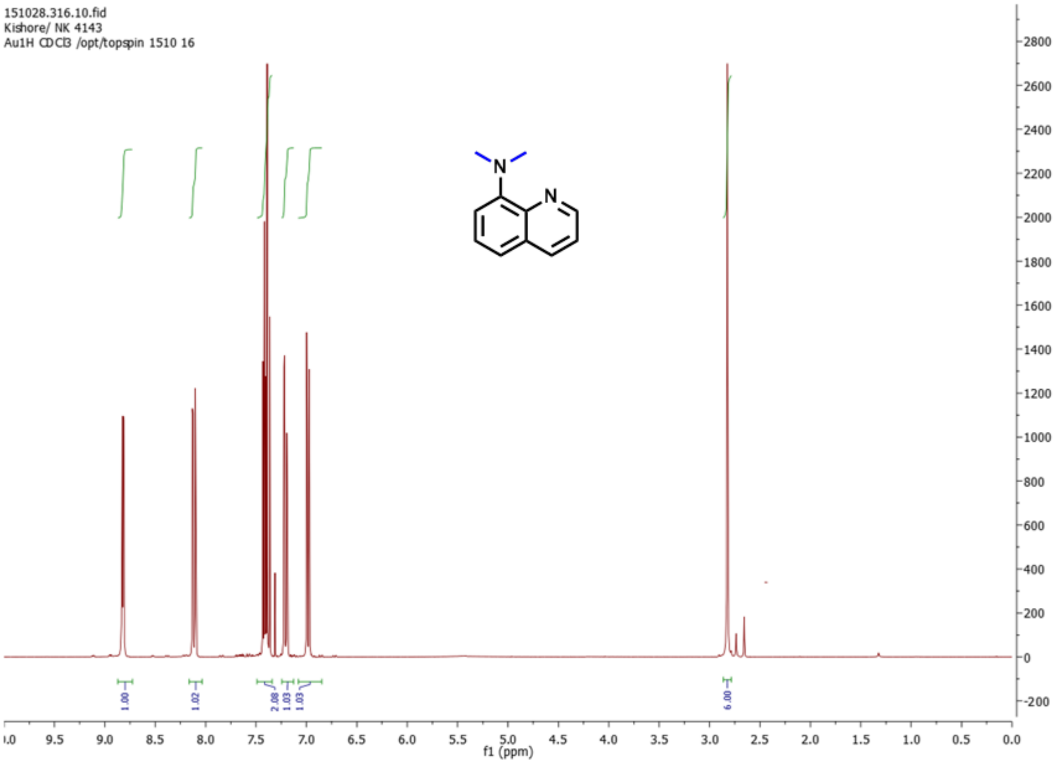

Supplementary Figure 45. ^{1}H NMR of *N*,*N*-Dimethylquinolin-8-amine

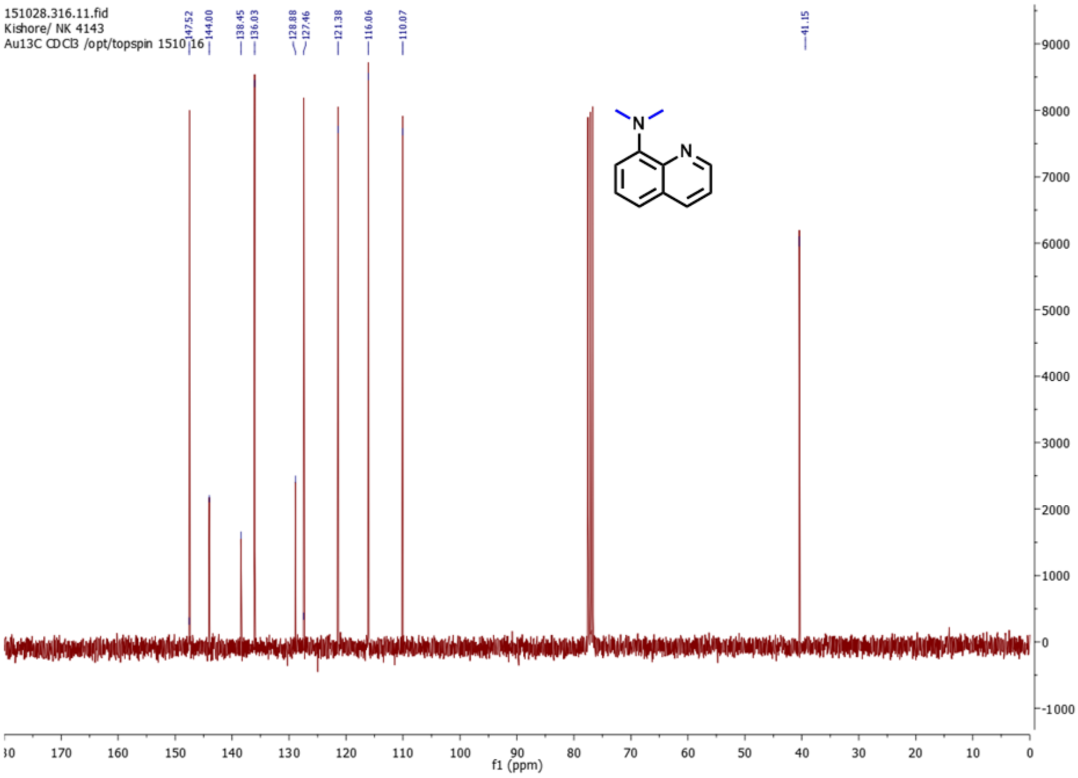

Supplementary Figure 46. ^{13}C NMR of *N*,*N*-Dimethylquinolin-8-amine

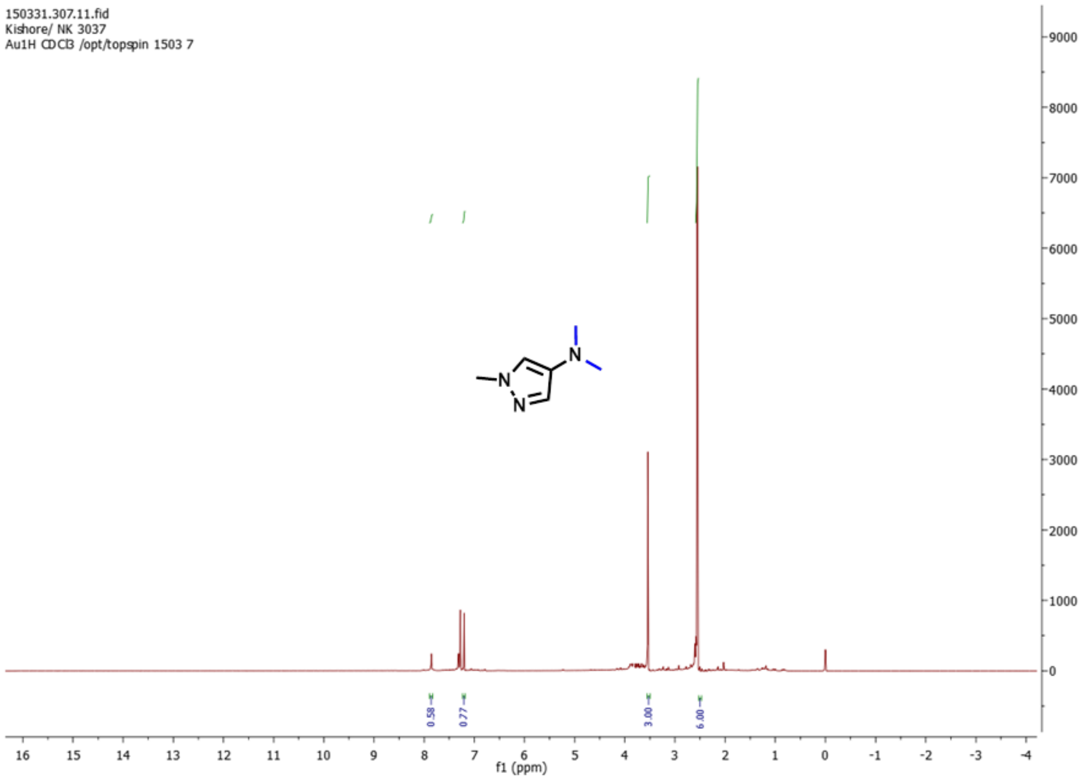

Supplementary Figure 47. ^{1}H NMR of *N*,*N*,1-Trimethyl-1H-pyrazol-4-amine

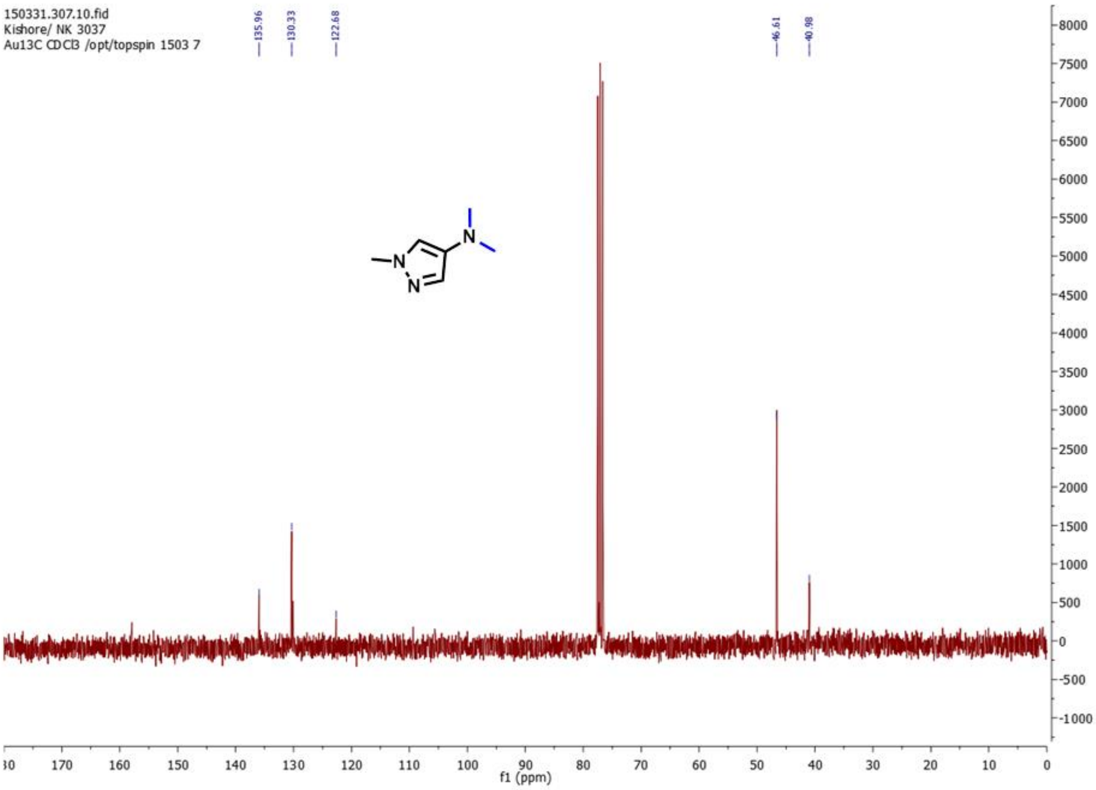

Supplementary Figure 48. ^{13}C NMR of *N*,*N*,1-Trimethyl-1H-pyrazol-4-amine

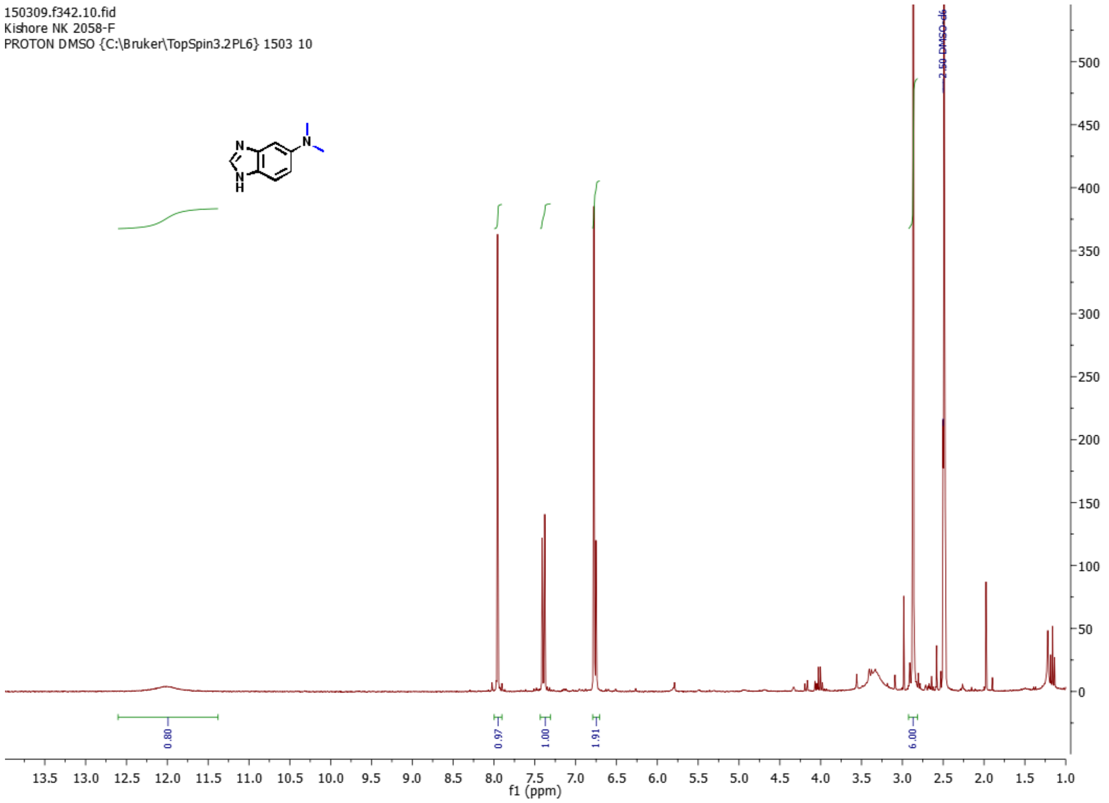

Supplementary Figure 49. ^{1}H NMR of *N*,*N*-dimethyl-1*H*-benzo[d]imidazol-5-amine

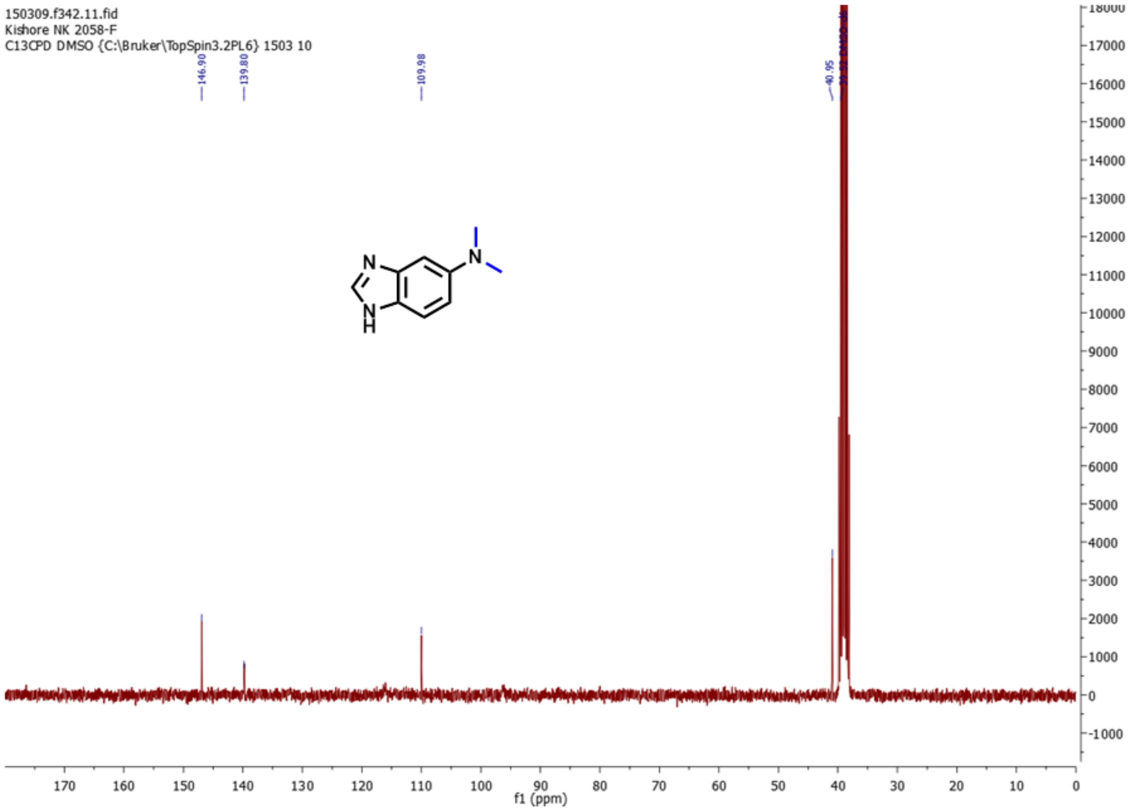

Supplementary Figure 50. ^{13}C NMR of *N*,*N*-dimethyl-1*H*-benzo[d]imidazol-5-amine

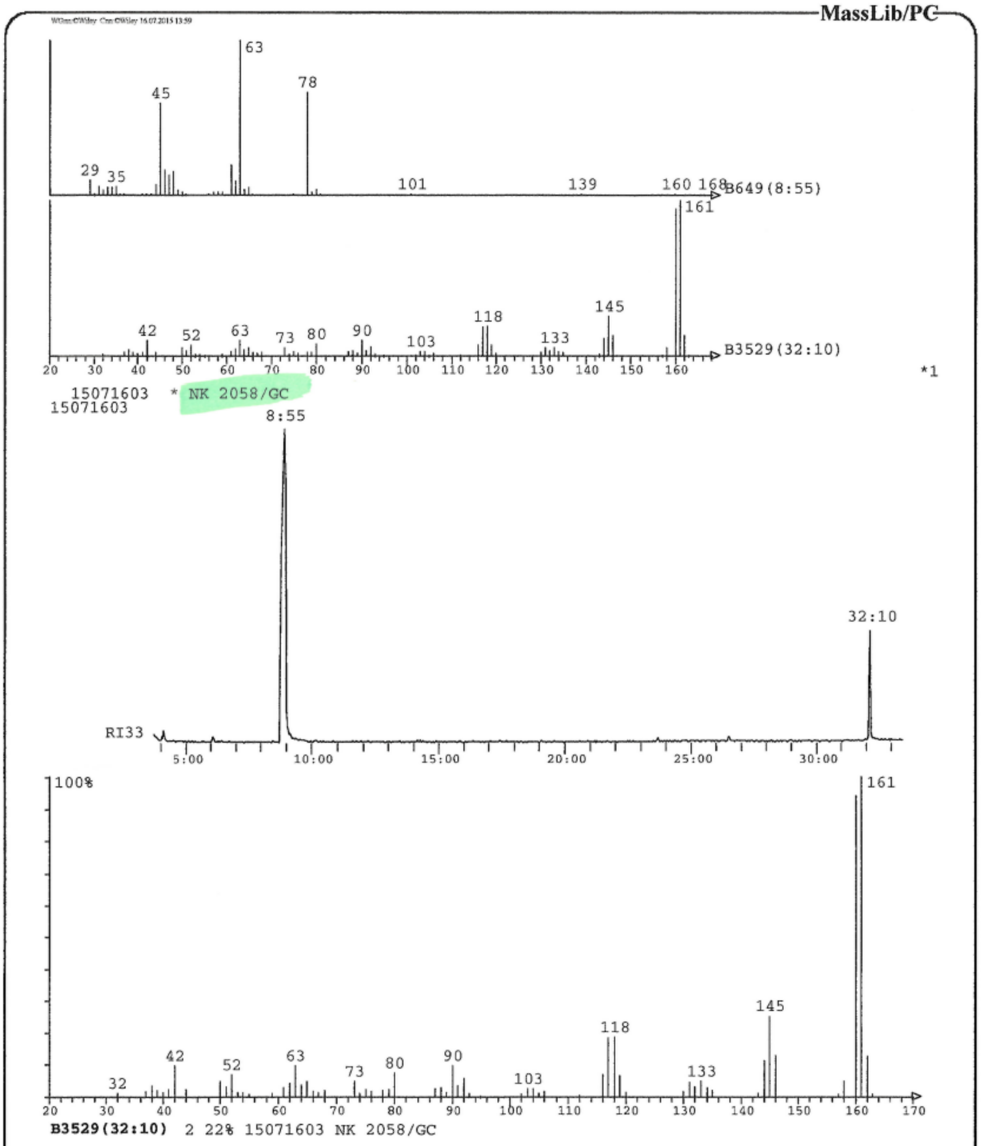

32	0.87	67	1.14	106	1.28	162	12.43
37	1.53	68	1.68	112	0.51	163	0.59
38	3.21	73	4.57	116	6.66		
39	1.74	74	0.87	117	18.14		
40	1.19	75	2.04	118	18.45		
41	1.88	76	1.28	119	6.40		
42	9.43	78	1.82	120	1.13		
44	1.82	79	1.82	130	1.49		
50	4.45	80	7.19	131	4.42		
51	2.86	87	2.18	132	2.97		
52	6.71	88	2.65	133	4.53		
53	1.06	89	1.12	134	2.47		
54	0.97	90	9.58	135	1.75		
55	0.58	91	3.01	143	0.85		
59	0.78	92	5.43	144	10.85		
61	2.57	93	0.84	145	25.02		
62	4.10	95	0.35	146	12.78		
63	9.37	102	0.63	157	0.56		
64	3.28	103	2.36	158	4.57		
65	4.66	104	2.16	160	94.08		
66	1.45	105	0.70	161	100.00		

B3529(32:10) 2 22% 15071603 NK 2058/GC

MassLib/PC V9.2 3063 Admin,IfOK, Rostock

Supplementary Figure 51. GC-MS of *N*,*N*-dimethyl-1*H*-benzo[d]imidazol-5-amine

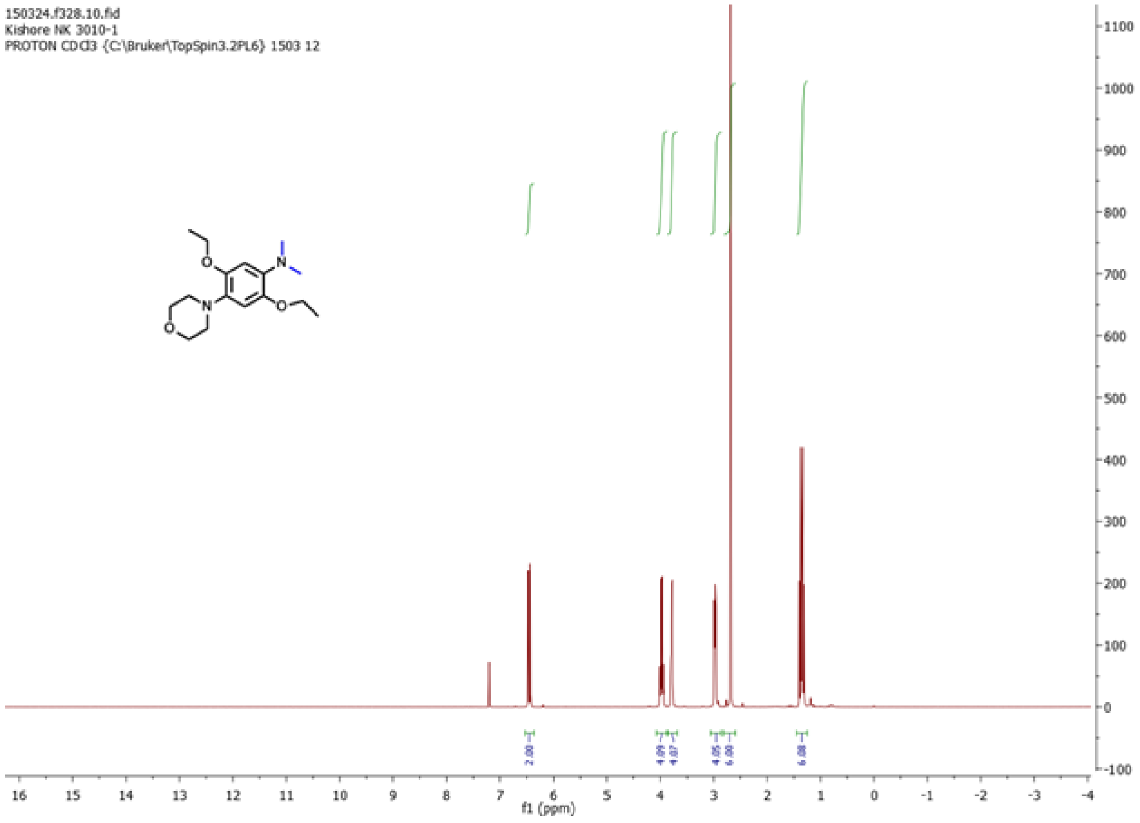

Supplementary Figure 52. ^{1}H NMR of 2,5-diethoxy-*N*,*N*-dimethyl-4-morpholinoaniline

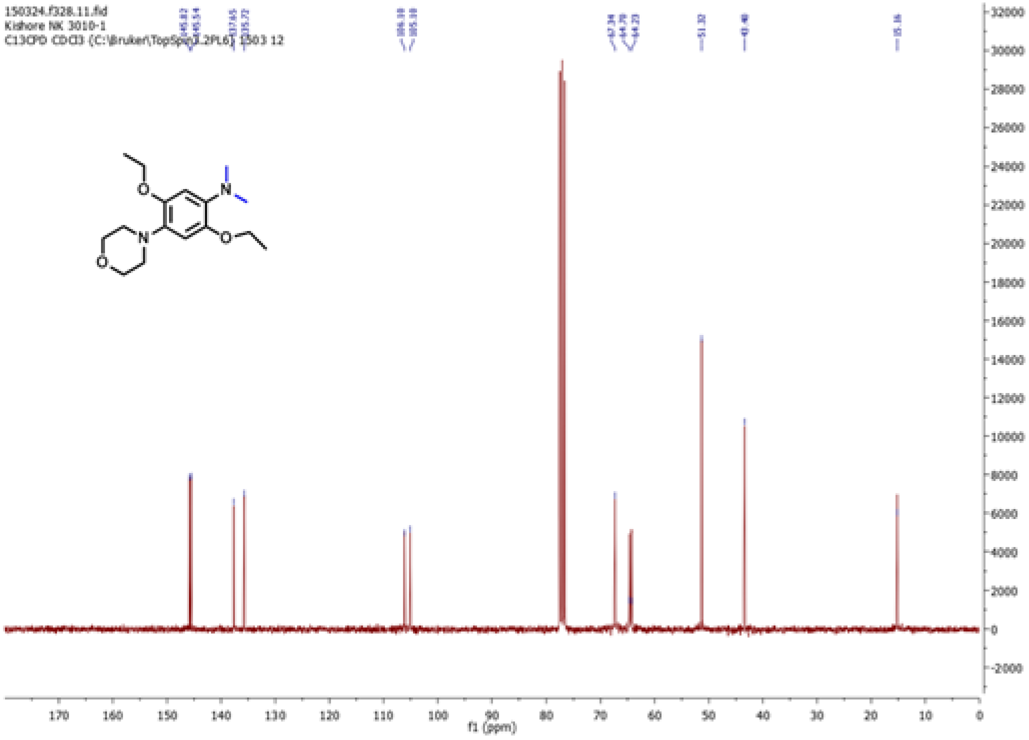

Supplementary Figure 53. ^{13}C NMR of 2,5-diethoxy-*N*,*N*-dimethyl-4-morpholinoaniline

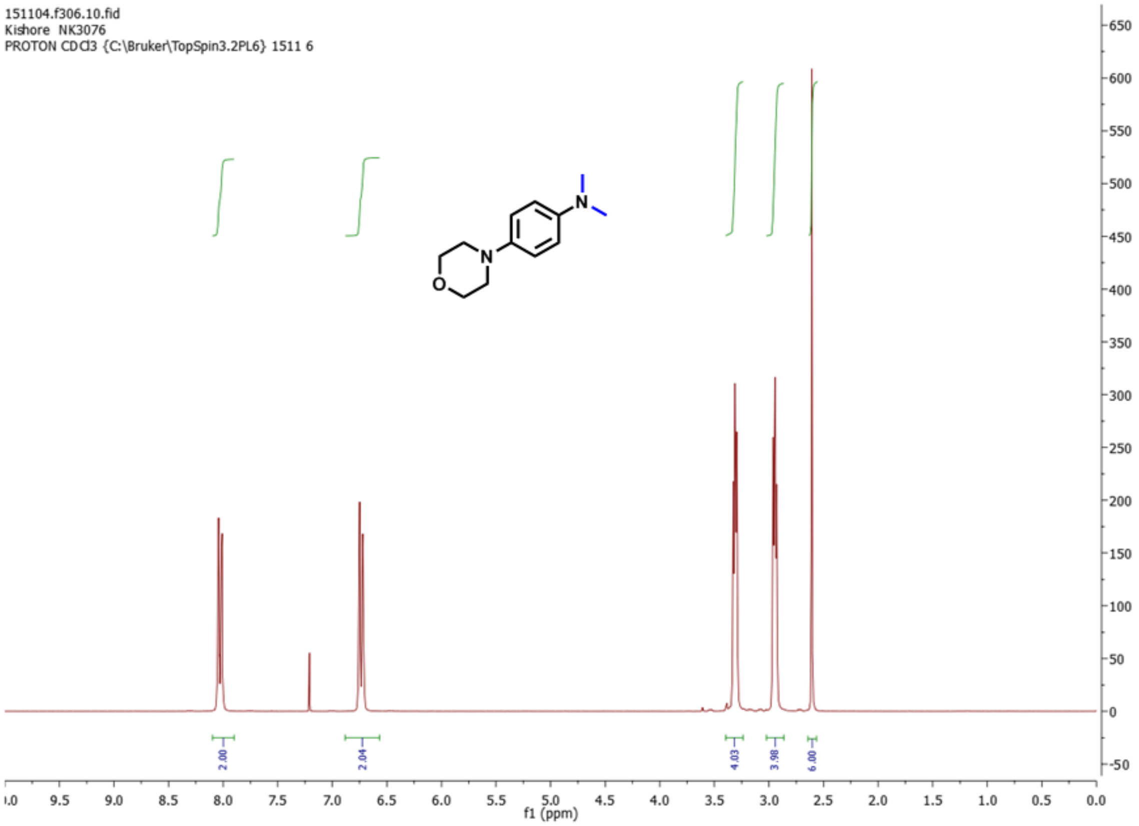

Supplementary Figure 54. ^{1}H NMR of *N*,*N*-dimethyl-4-morpholinoaniline

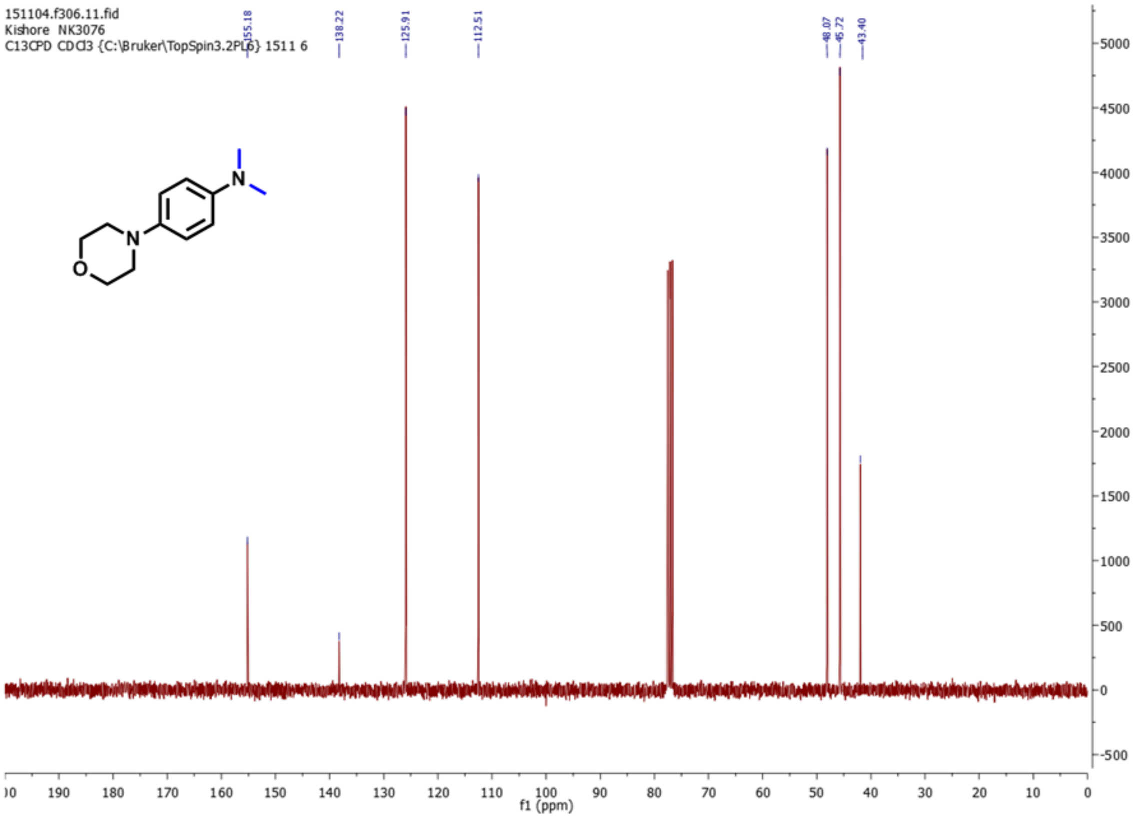

Supplementary Figure 55. ^{13}C NMR of *N*,*N*-dimethyl-4-morpholinoaniline

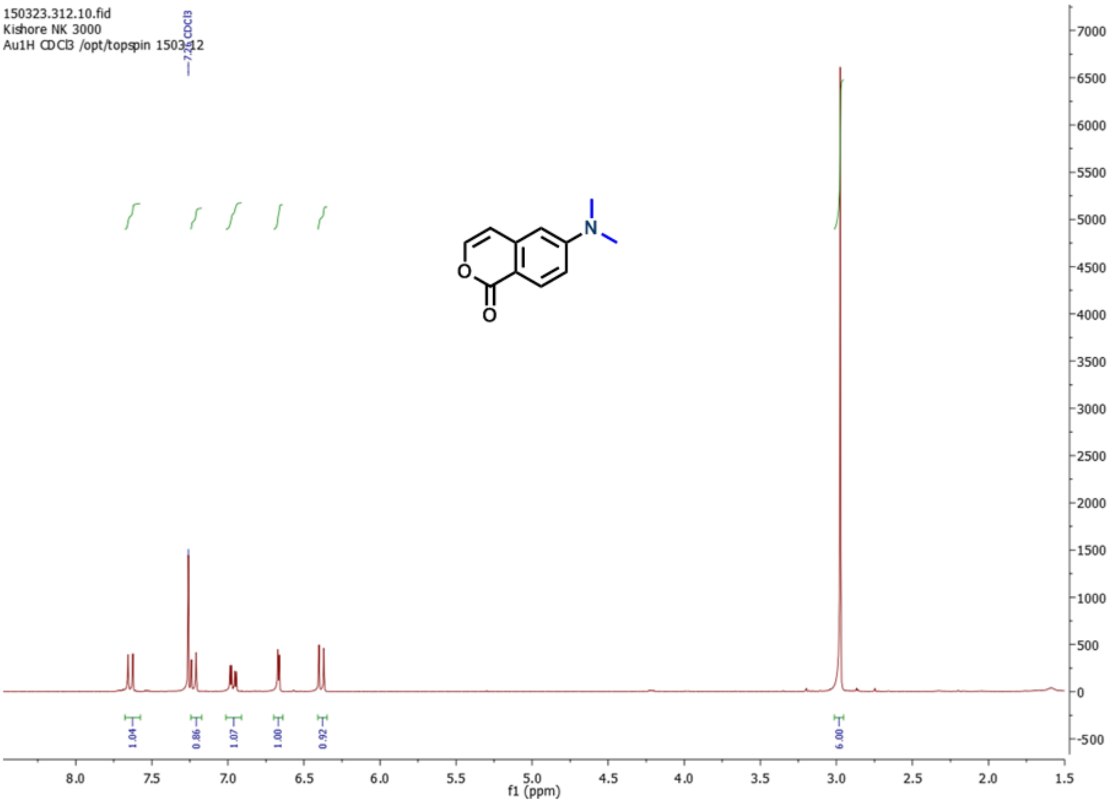

Supplementary Figure 56. ^{1}H NMR of 6-(Dimethylamino)-1*H*-isochromen-1-one

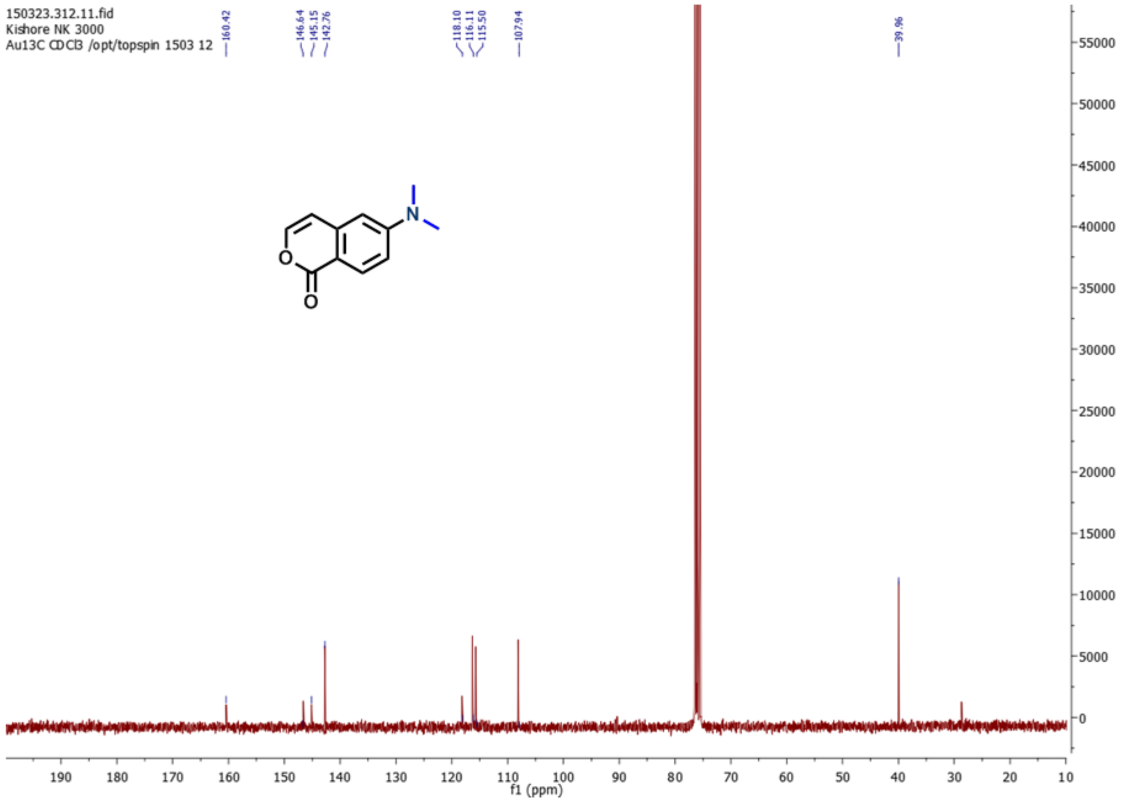

Supplementary Figure 57. ^{13}C NMR of 6-(Dimethylamino)-1*H*-isochromen-1-one

Qualitative Compound Report

**Instrument Name**	ESI-TOF/MS	**Date Filename**	D:\MassHunter\Data\1505\15052011.d
**Acq Method**	HRMS Pos oS.m	**Sample Name**	NK3003
**DA Method**	HRMS.m	**Position**	Vial 72
**User Name**	Fischer	**Comment**	MeOH/0,1%HCOOH in H2O 90 10

Compound Table

Name	RT	Abund	Formula	Ion Mass	Ionization Mode
1	0.189	182031	C11 H11 N O2	189.079	Positive

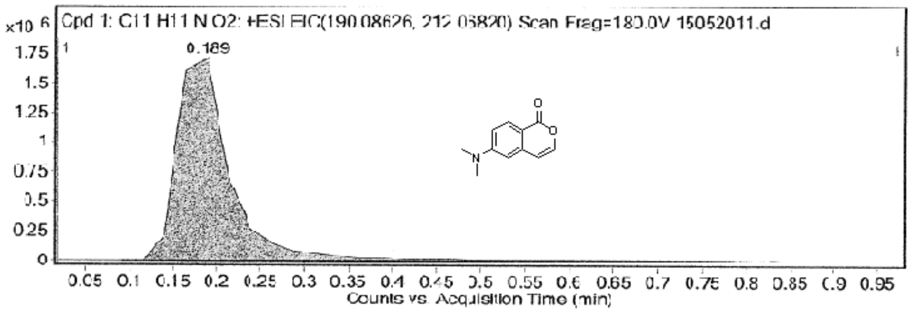

MS Zoomed Spectrum

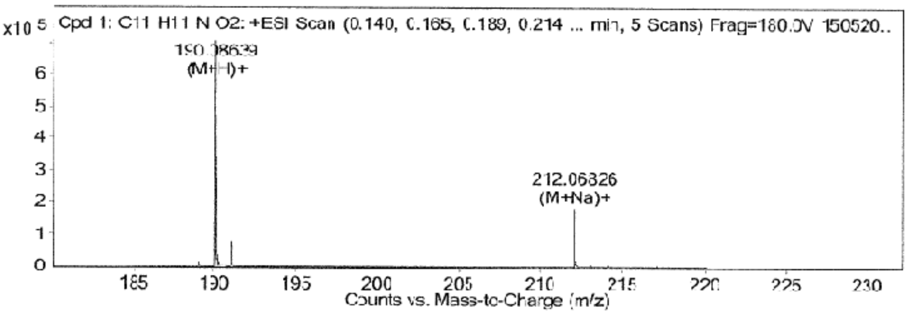

MS Spectrum Peak List

Ion	Abund	Formula	Calculated Mass	Measured Mass	Difference	Diff (ppm)
(M+H)+	720359.06	C11H11NO2	190.08626	190.08639	-0.14	-0.72
(M+Na)+	182031.31	C11H11NO2	212.0682	212.06826	-0.06	-0.27

--- End Of Report ---

Supplementary Figure 58. HRMS (High Resolution Mass Spectroscopy) of 6-(Dimethylamino)-1*H*-isochromen-1-one

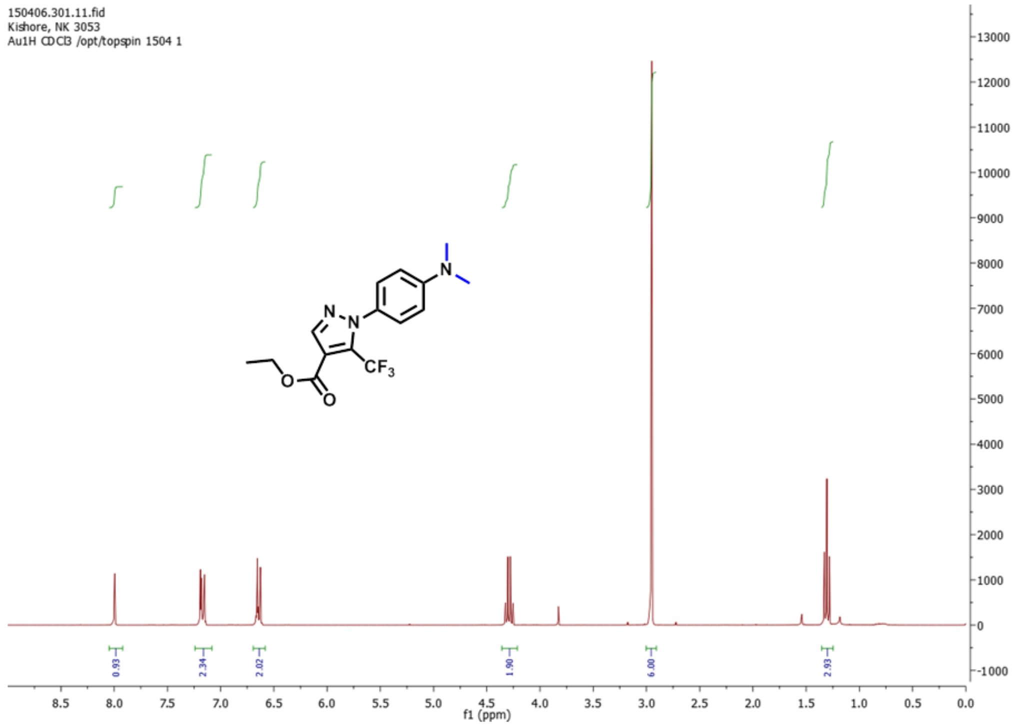

Supplementary Figure 59. ^{1}H NMR of Ethyl 1-(4-(dimethylamino)phenyl)-5-(trifluoromethyl)-1*H*-pyrazole-4-carboxylate

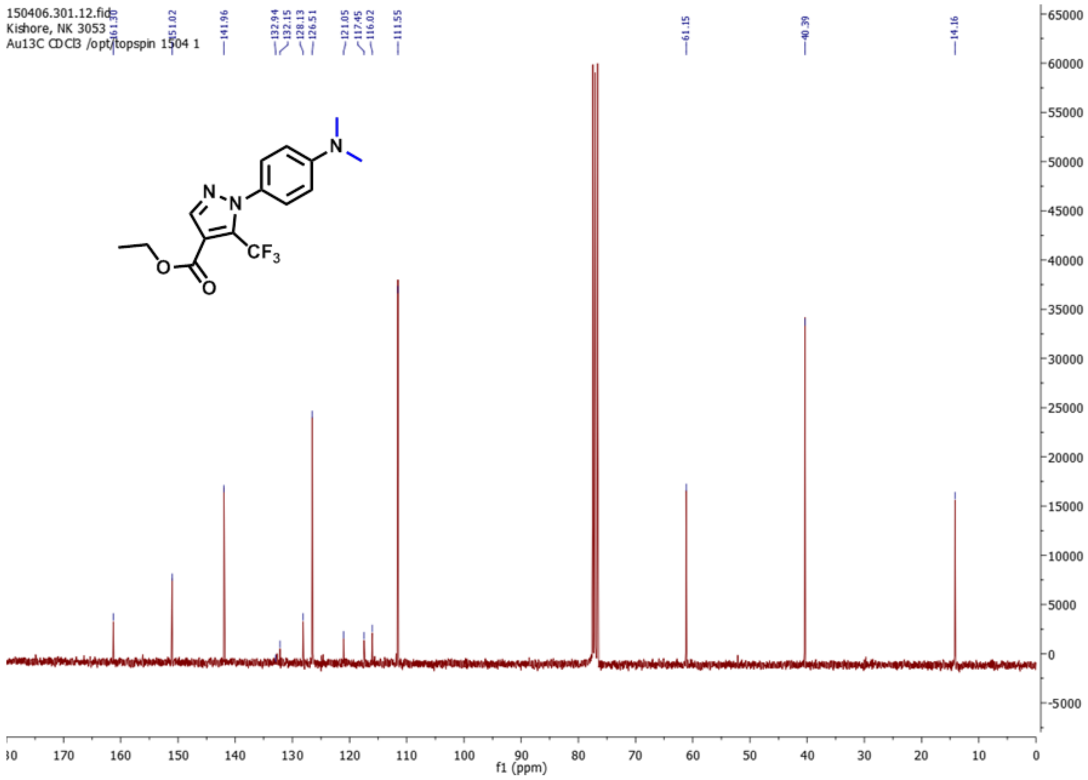

Supplementary Figure 60. ^{13}C NMR of Ethyl 1-(4-(dimethylamino)phenyl)-5-(trifluoromethyl)-1*H*-pyrazole-4-carboxylate

Qualitative Compound Report

**Instrument Name**	ESI-TOF/MS	**Date Filename**	D:\MassHunter\Data\1505\15051806.d
**Acq Method**	HRMS Pos oS.m	**Sample Name**	NK 3053
**DA Method**	HRMS.m	**Position**	Vial 56
**User Name**	Fischer	**Comment**	MeOH/0,1%HCOOH in H2O 90:10

Compound Table

Name	RT	Abund	Formula	Ion Mass	Ionization Mode
1	0.187	187678	C15 H16 F3 N3 O2	327.1195	Positive

MS Zoomed Spectrum

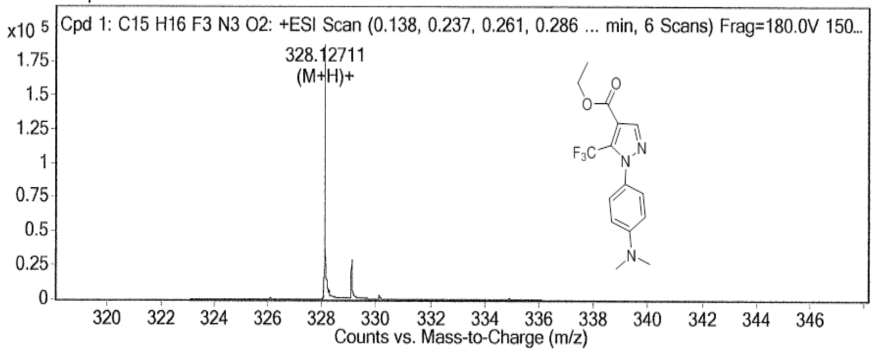

MS Spectrum Peak List

Ion	Abund	Formula	Calculated Mass	Measured Mass	Difference	Diff (ppm)
(M+H)+	187678.3	C15H16F3N3O2	328.12674	328.12711	-0.37	-1.14

--- End Of Report ---

Supplementary Figure 61. HRMS (High Resolution Mass Spectroscopy) of Ethyl 1-(4-(dimethylamino)phenyl)-5-(trifluoromethyl)-1H-pyrazole-4-carboxylate

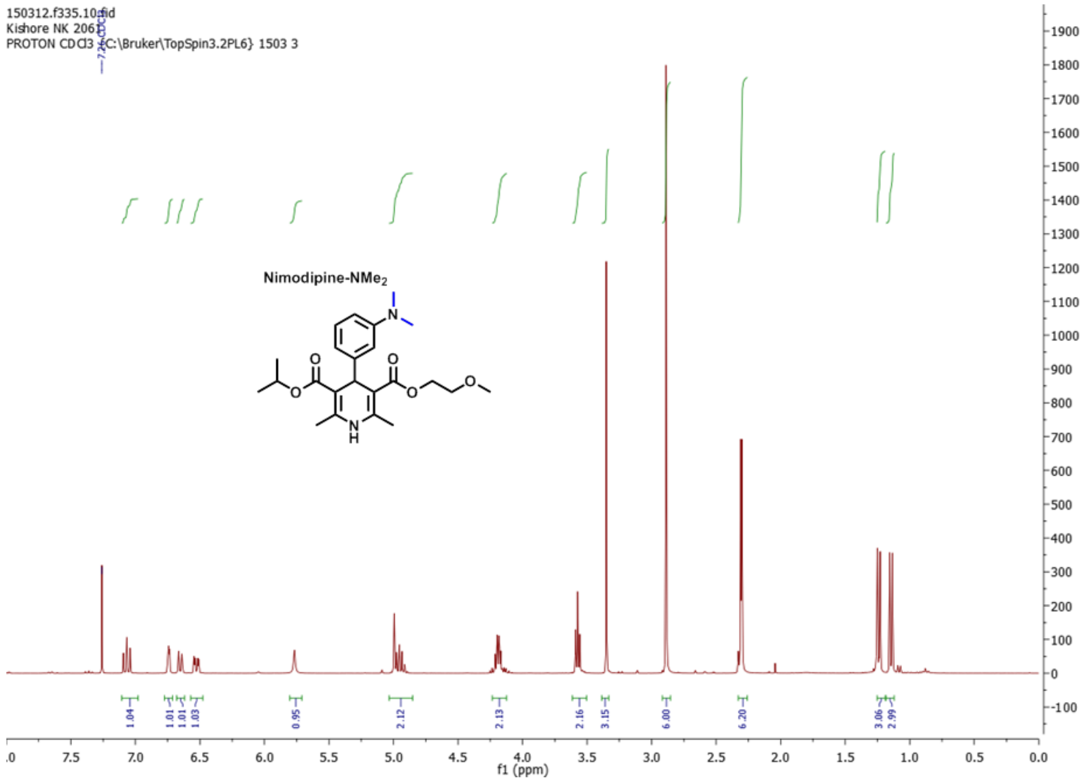

Supplementary Figure 62. ^{1}H NMR of Nimodipine-NMe2

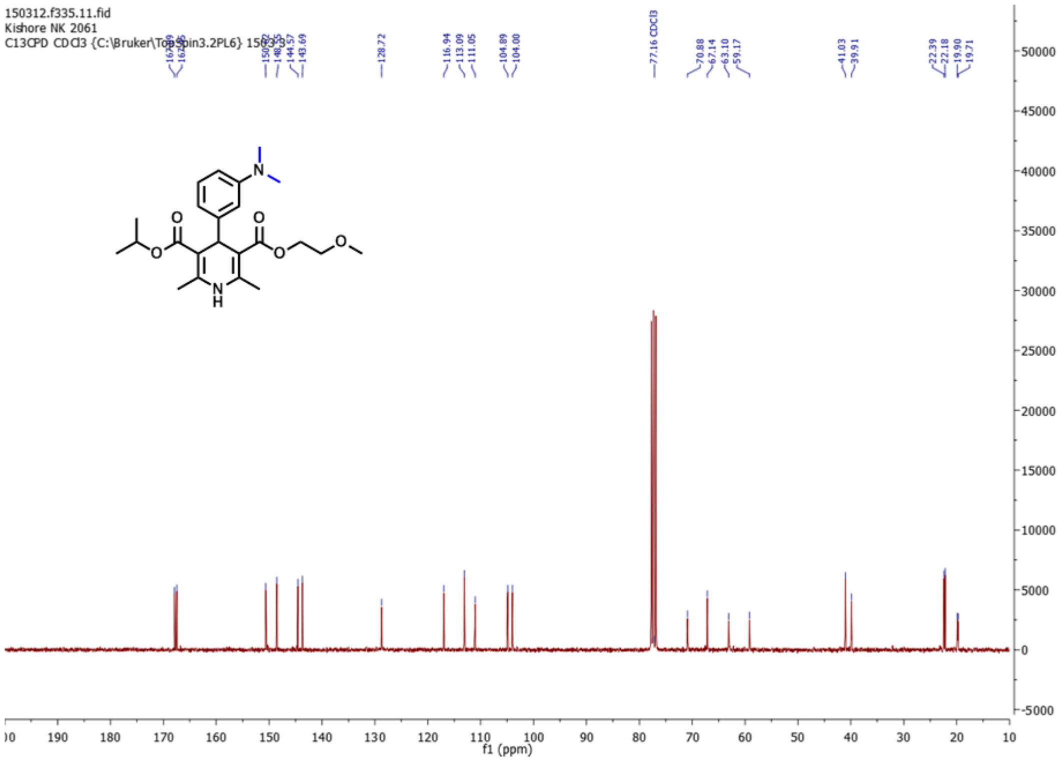

Supplementary Figure 63. ^{13}C NMR of Nimodipine-NMe2

```
File       :D:\MSDChem\1\DATA\1505\NK4076.D
Operator   : Sandra
Acquired   :  2 Jun 2015   6:39    using AcqMethod KLAUSX.M
Instrument :    GC-MSD
Sample Name: NK4076
Misc Info  :
Vial Number: 7
```

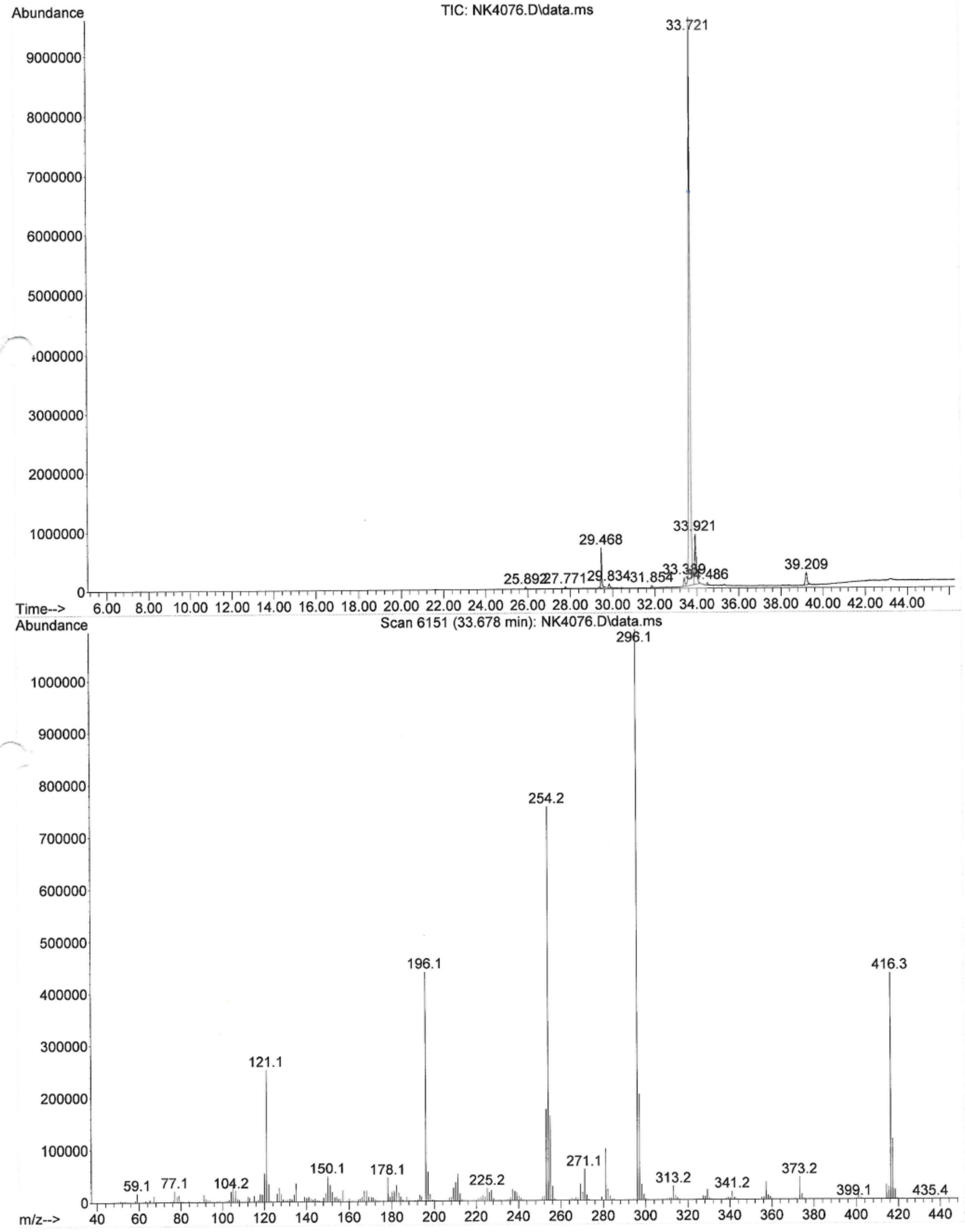

Supplementary Figure 64. GC-MS of Nimodipine-NMe2 (gram scale synthesis)

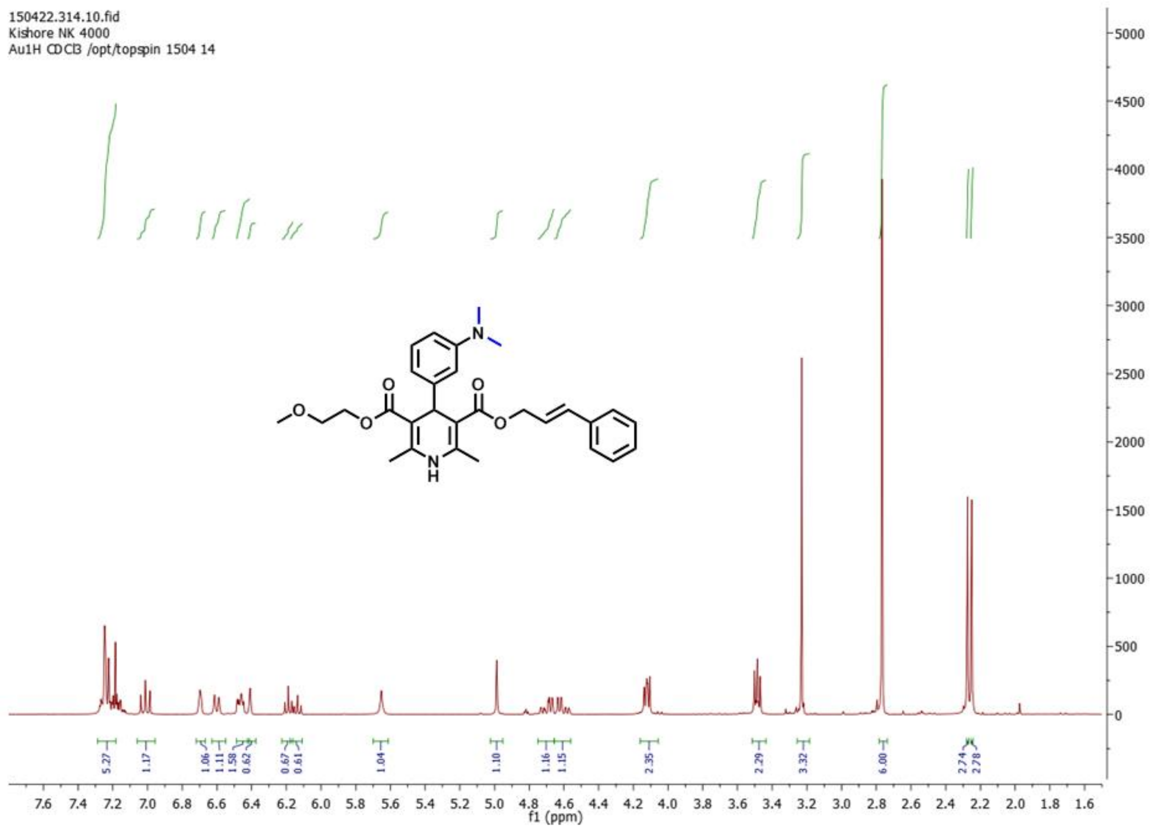

Supplementary Figure 65. ^{1}H NMR of Clinidipine-NMe2

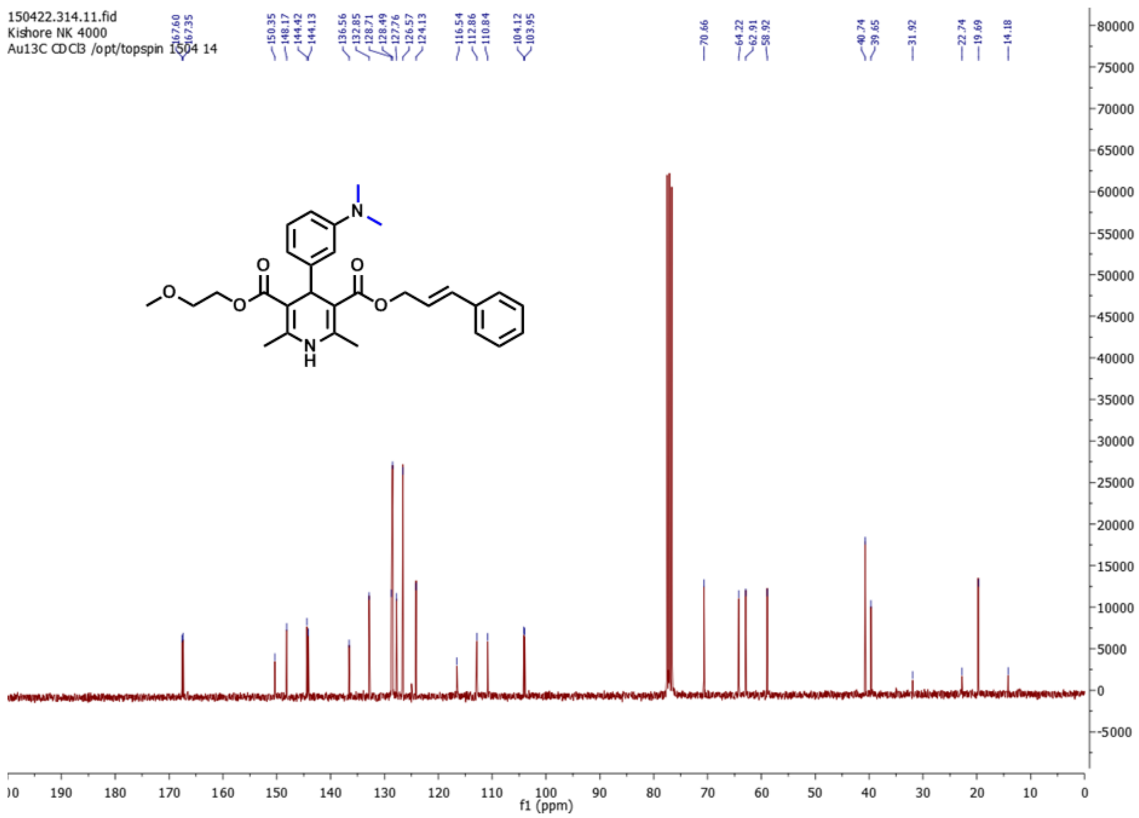

Supplementary Figure 66. ^{13}C NMR of Clinidipine-NMe2

Qualitative Compound Report

**Instrument Name**	ESI-TOF/MS	**Date Filename**	D:\MassHunter\Data\1505\15051908.d
**Acq Method**	HRMS Pos oS.m	**Sample Name**	NK4000
**DA Method**	HRMS.m	**Position**	Vial 78
**User Name**	Fischer	**Comment**	MeOH/0.1%HCOOH in H2O 90:10

Compound Table

Name	RT	Abund	Formula	Ion Mass	Ionization Mode
1	0.173	723853	C29 H34 N2 O5	490.2468	Positive

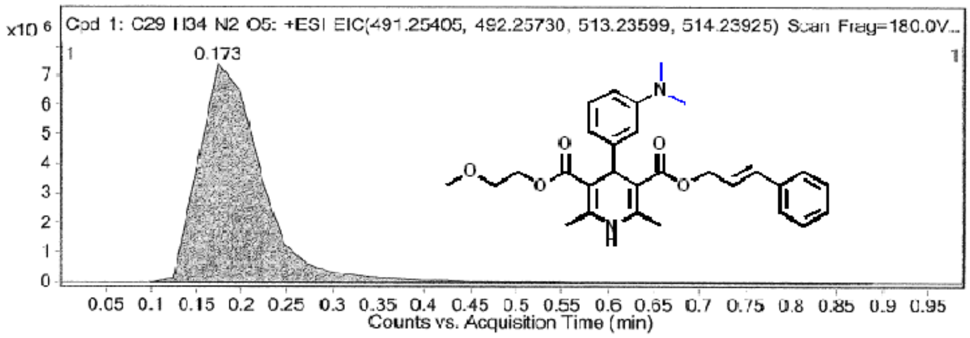

MS Zoomed Spectrum

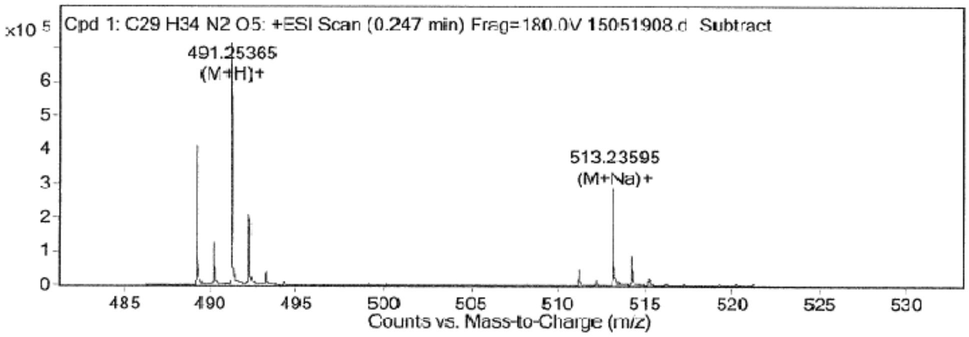

MS Spectrum Peak List

Ion	Abund	Formula	Calculated Mass	Measured Mass	Difference	Diff (ppm)
(M+H)+	723853.19	C29H34N2O5	491.25405	491.25365	0.4	0.81
(M+Na)+	283310.25	C29H34N2O5	513.23599	513.23595	0.04	0.08

--- End Of Report ---

Supplementary Figure 67. HRMS (High Resolution Mass Spectroscopy) of Clinidipine-NMe2

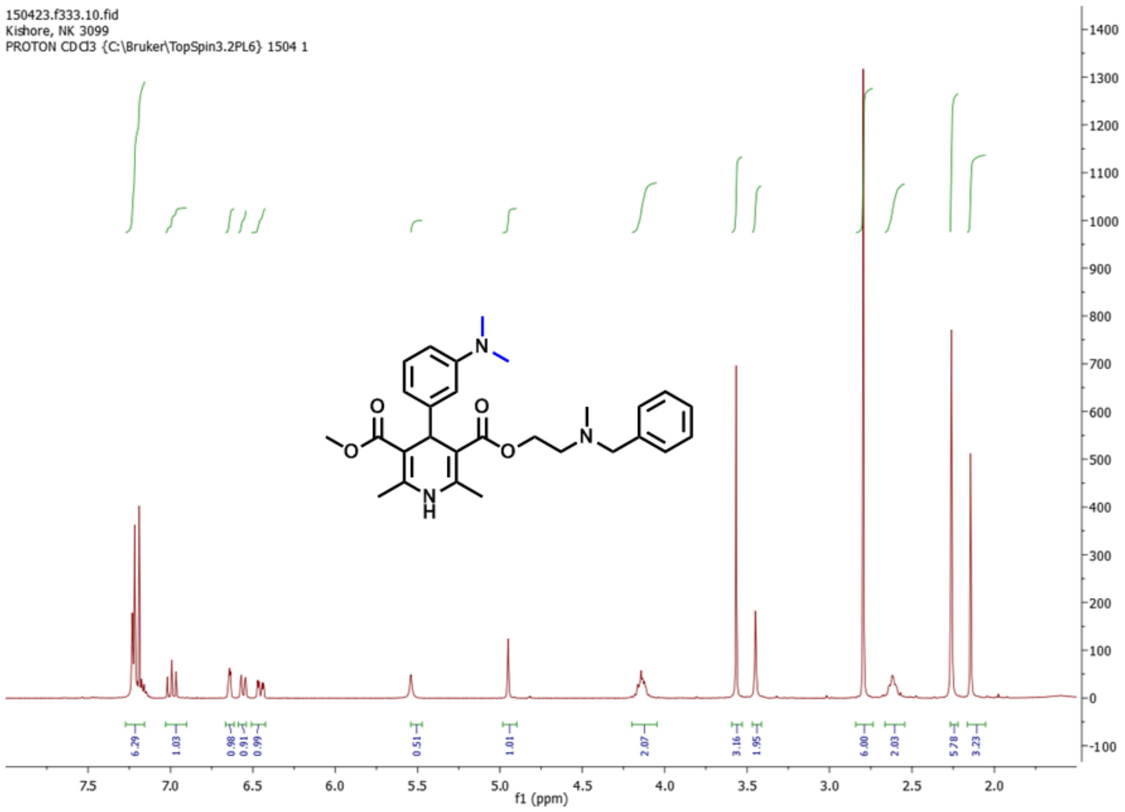

Supplementary Figure 68. ^{1}H NMR of Nicardipine-NMe2

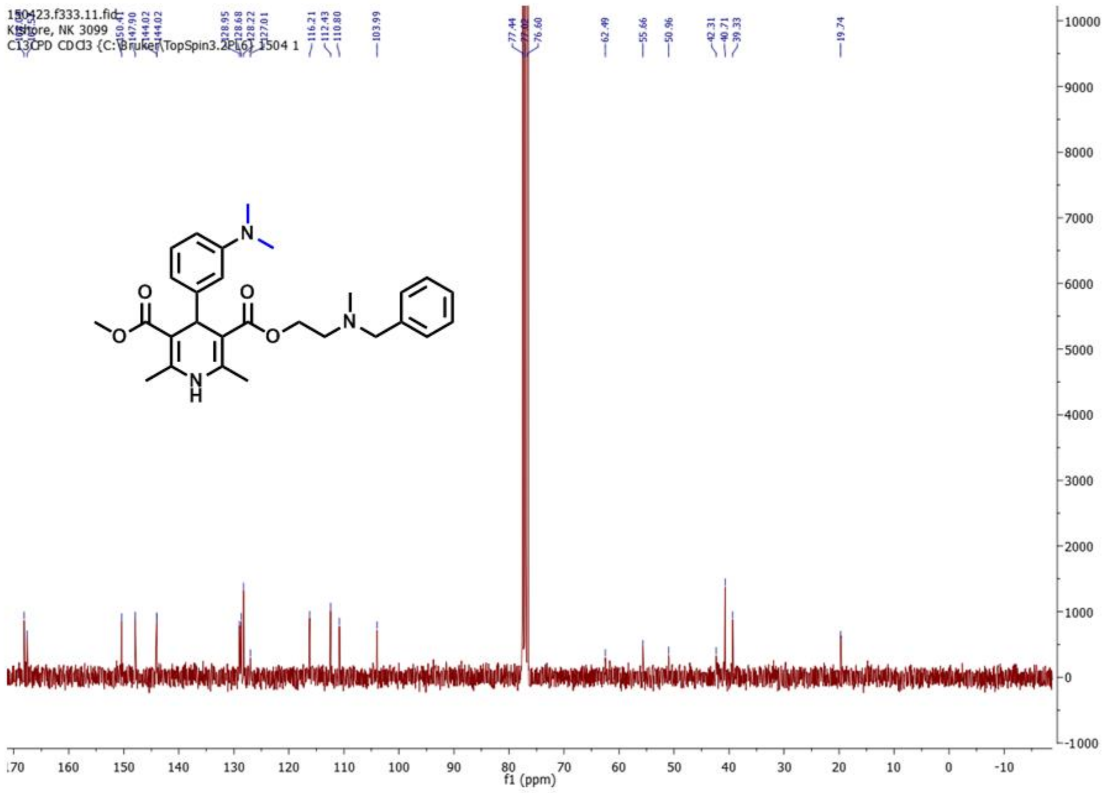

Supplementary Figure 69. ^{13}C NMR of Nicardipine-NMe2

Qualitative Compound Report

**Instrument Name**	ESI-TOF/MS	**Data Filename**	D:\MassHunter\Data\1505\15051909.d
**Acq Method**	HRMS Pos cS.m	**Sample Name**	NK3099
**DA Method**	HRMS.m	**Position**	Vial 79
**User Name**	Fischer	**Comment**	MeOH/0,1%HCOOH in H2O 90:10

Compound Table

Name	RT	Abund	Formula	Ion Mass	Ionization Mode
1	0.185	175230	C28 H35 N3 O4	477.2528	Positive

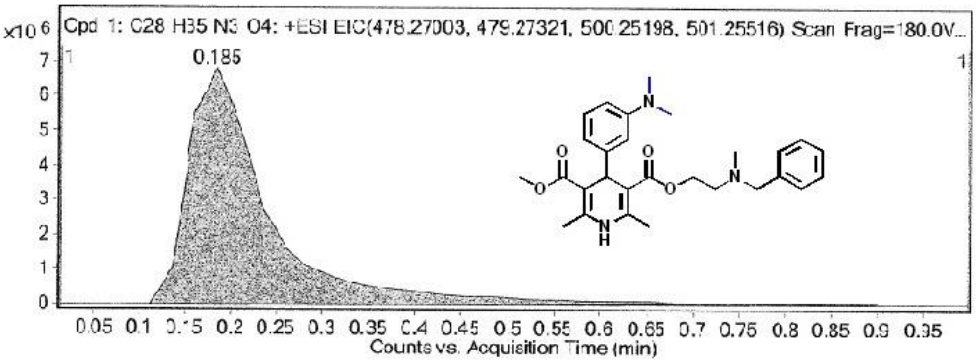

MS Zoomed Spectrum

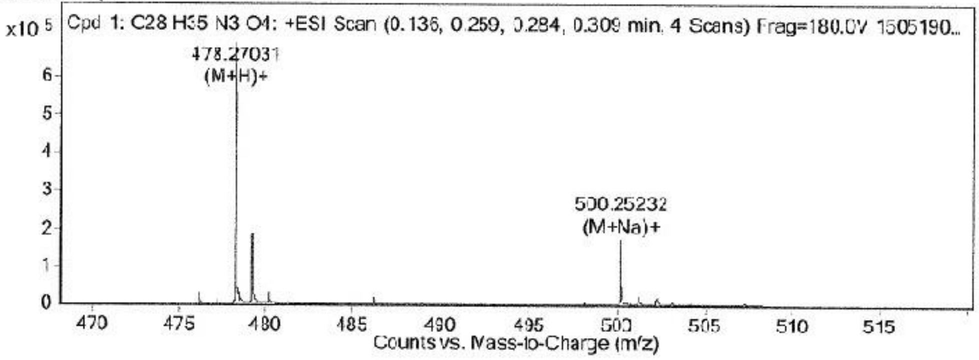

MS Spectrum Peak List

Ion	Abund	Formula	Calculated Mass	Measured Mass	Difference	Diff (ppm)
(M+H)+	694086.88	C28H35N3O4	478.27003	478.27031	-0.28	-0.58
(M+Na)+	176279.83	C28H35N3O4	500.25198	500.25232	-0.35	-0.69

--- End Of Report ---

Supplementary Figure 70. HRMS (High Resolution Mass Spectroscopy) of Nicardipine-NMe2

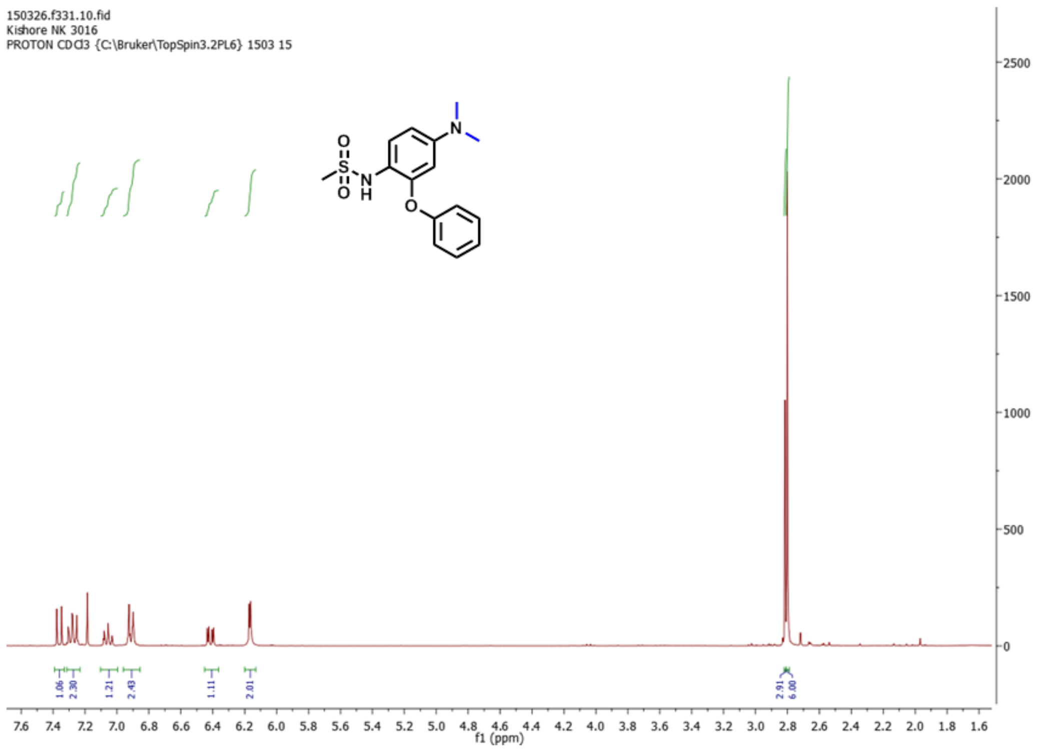

Supplementary Figure 71. ^{1}H NMR of Nimisulide-NMe2

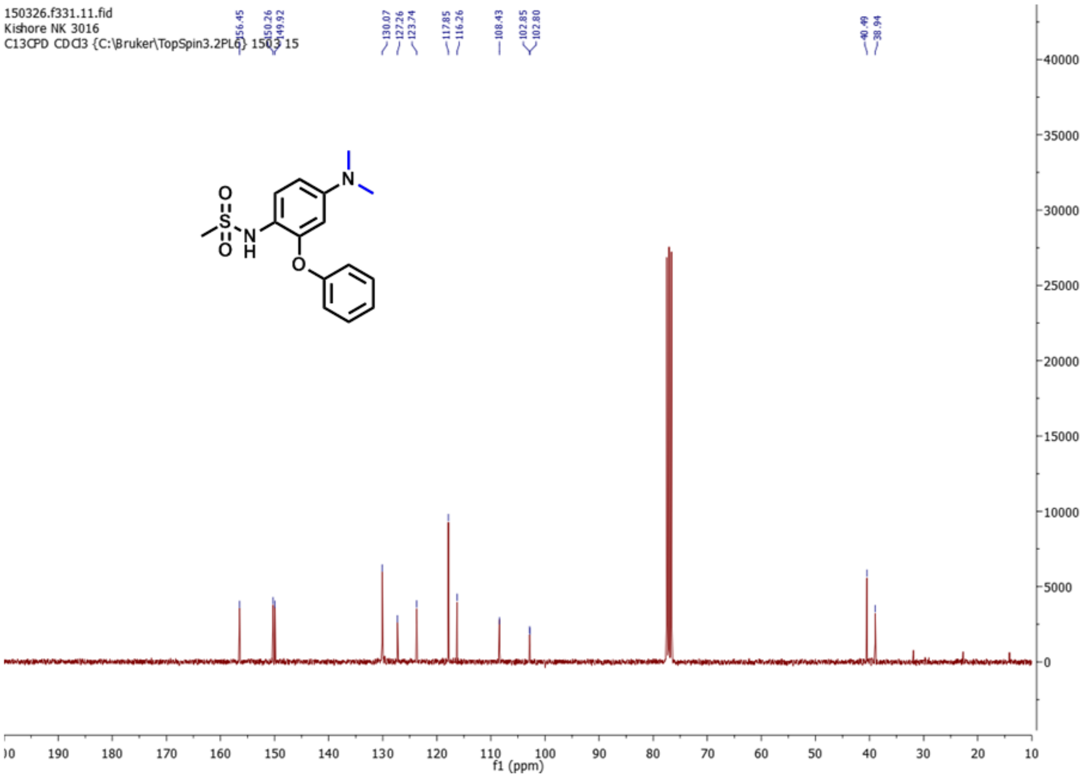

Supplementary Figure 72. ^{13}C NMR of Nimisulide-NMe2

Qualitative Compound Report

**Instrument Name**	ESI-TOF/MS	**Date Filename**	D:\MassHunter\Data\1505\15051803.d
**Acq Method**	HRMS Pos oS.m	**Sample Name**	NK 3016
**DA Method**	HRMS.m	**Position**	Vial 53
**User Name**	Fischer	**Comment**	MeOH/0,1%HCOOH in H2O 90:10

Compound Table

Name	RT	Abund	Formula	Ion Mass	Ionization Mode
1	0.184	101559	C15 H18 N2 O3 S	306.1038	Positive

MS Zoomed Spectrum

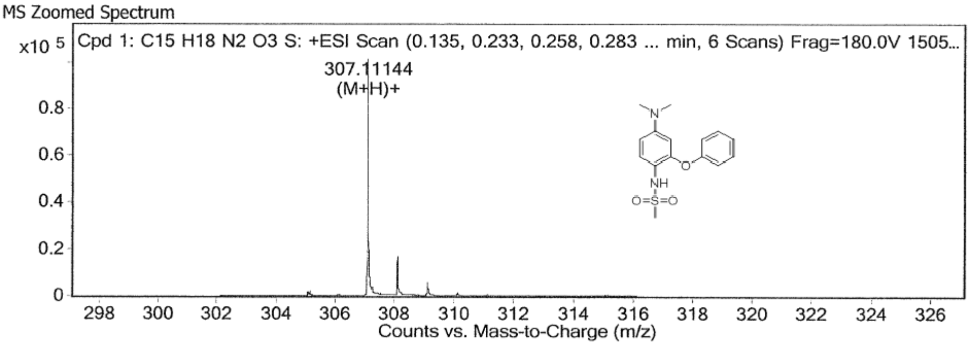

MS Spectrum Peak List

Ion	Abund	Formula	Calculated Mass	Measured Mass	Difference	Diff (ppm)
(M+H)+	101558.73	C15H18N2O3S	307.11109	307.11144	-0.35	-1.13

--- End Of Report ---

Supplementary Figure 73. HRMS (High Resolution Mass Spectroscopy) of Nimisulide-NMe2

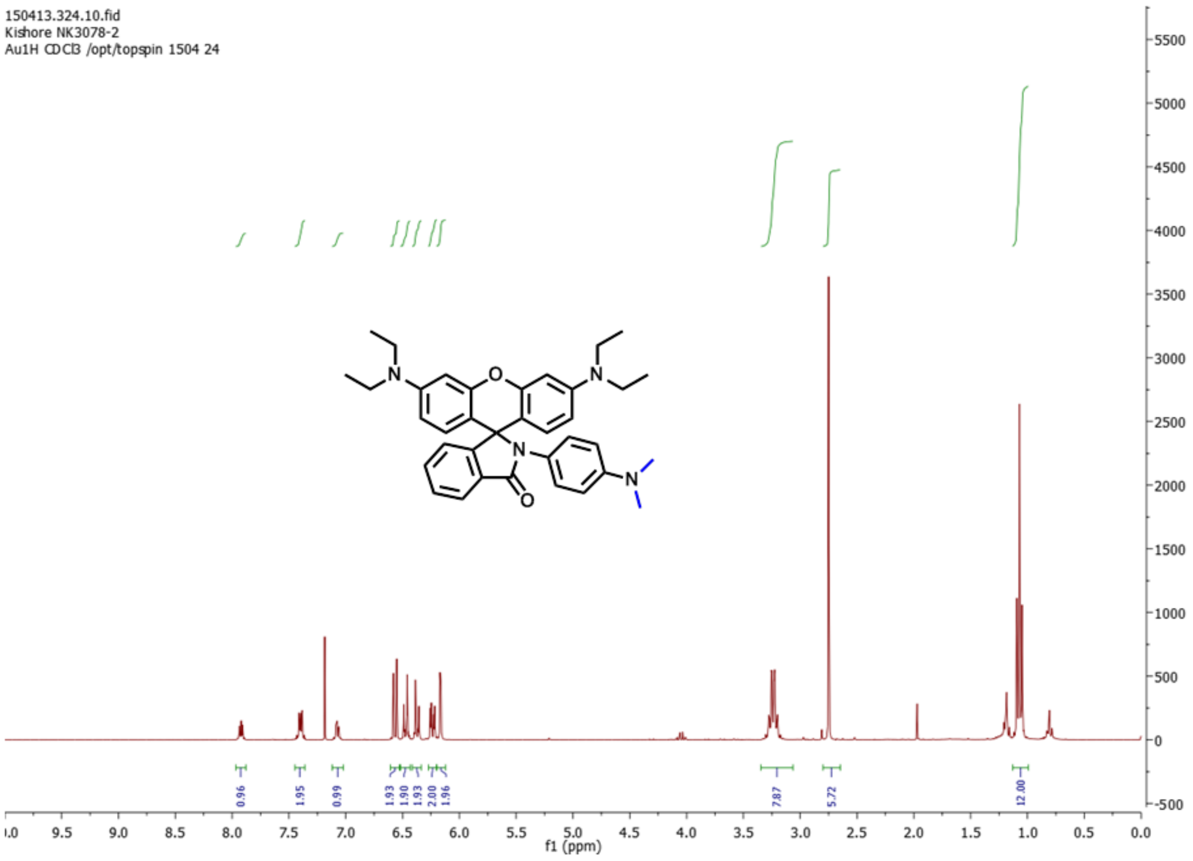

Supplementary Figure 74. ^{1}H NMR of 3',6'-bis(Diethylamino)-2-(4-(dimethylamino)phenyl)spiro[isoindoline-1,9'-xanthen]-3-one

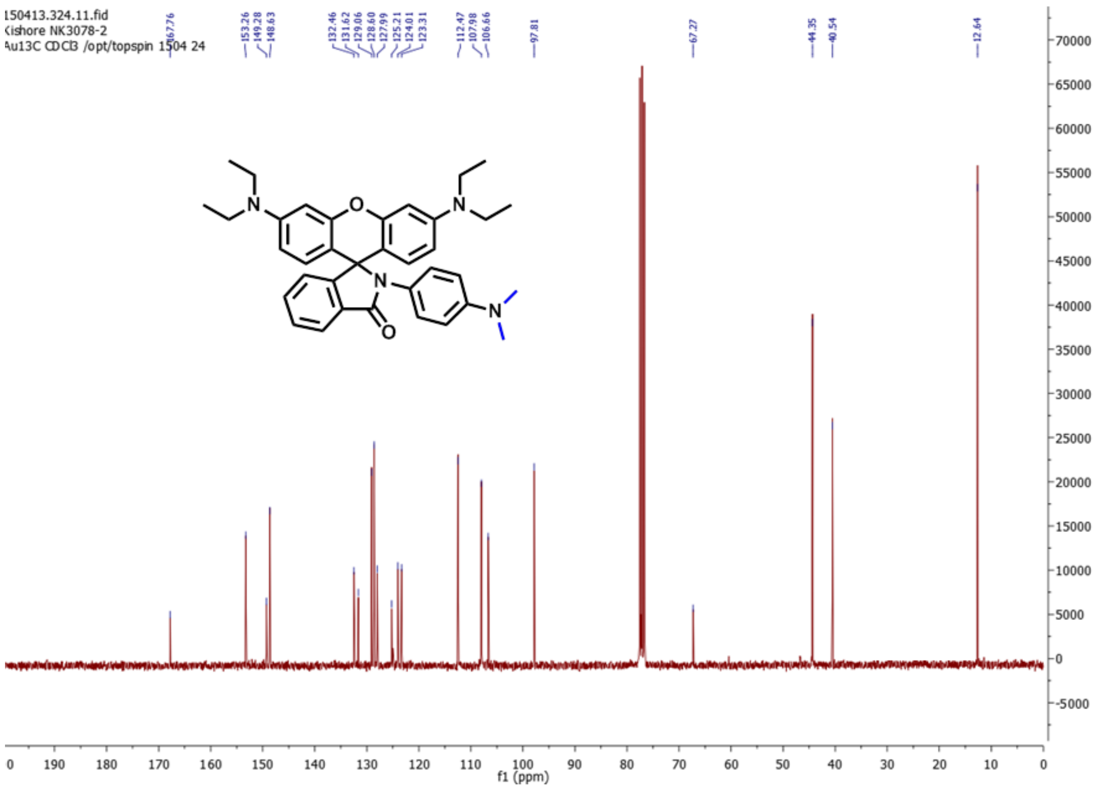

Supplementary Figure 75. ^{13}C NMR of 3',6'-bis(Diethylamino)-2-(4-(dimethylamino)phenyl)spiro[isoindoline-1,9'-xanthen]-3-one

Qualitative Compound Report

**Instrument Name**	ESI-TOF/MS	**Date Filename**	D:\MassHunter\Data\1505\15052022.d
**Acq Method**	HRMS Pos oS.m	**Sample Name**	NK3078-2*
**DA Method**	HRMS.m	**Position**	Vial 64
**User Name**	Fischer	**Comment**	MeOH/0,1%HCOOH in H2O 90:10

Compound Table

Name	RT	Abund	Formula	Ion Mass	Ionization Mode
1	0.171	291099	C36 H40 N4 O2	560.3151	Positive

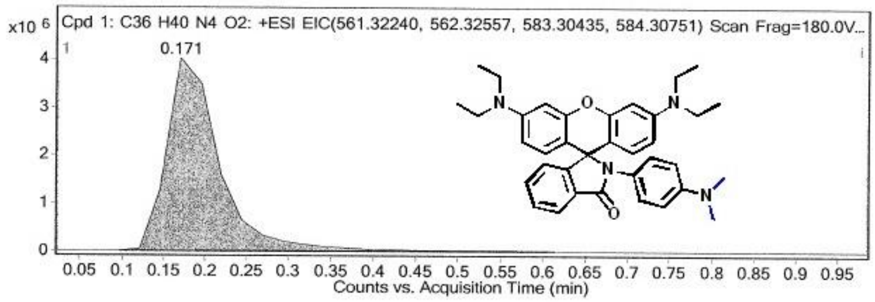

MS Zoomed Spectrum

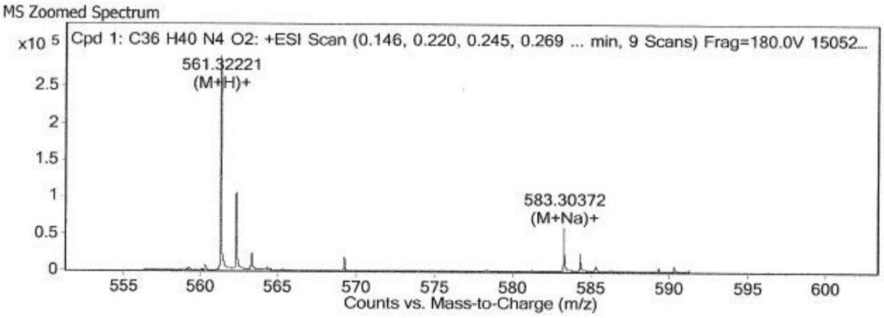

MS Spectrum Peak List

Ion	Abund	Formula	Calculated Mass	Measured Mass	Difference	Diff (ppm)
(M+H)+	291099.31	C36H40N4O2	561.3224	561.32221	0.19	0.34
(M+Na)+	58504.85	C36H40N4O2	583.30435	583.30372	0.63	1.08

--- End Of Report ---

Supplementary Figure 76. HRMS (High Resolution Mass Spectroscopy) of 3',6'-bis(Diethylamino)-2-(4-(dimethylamino)phenyl)spiro[isoindoline-1,9'-xanthen]-3-one

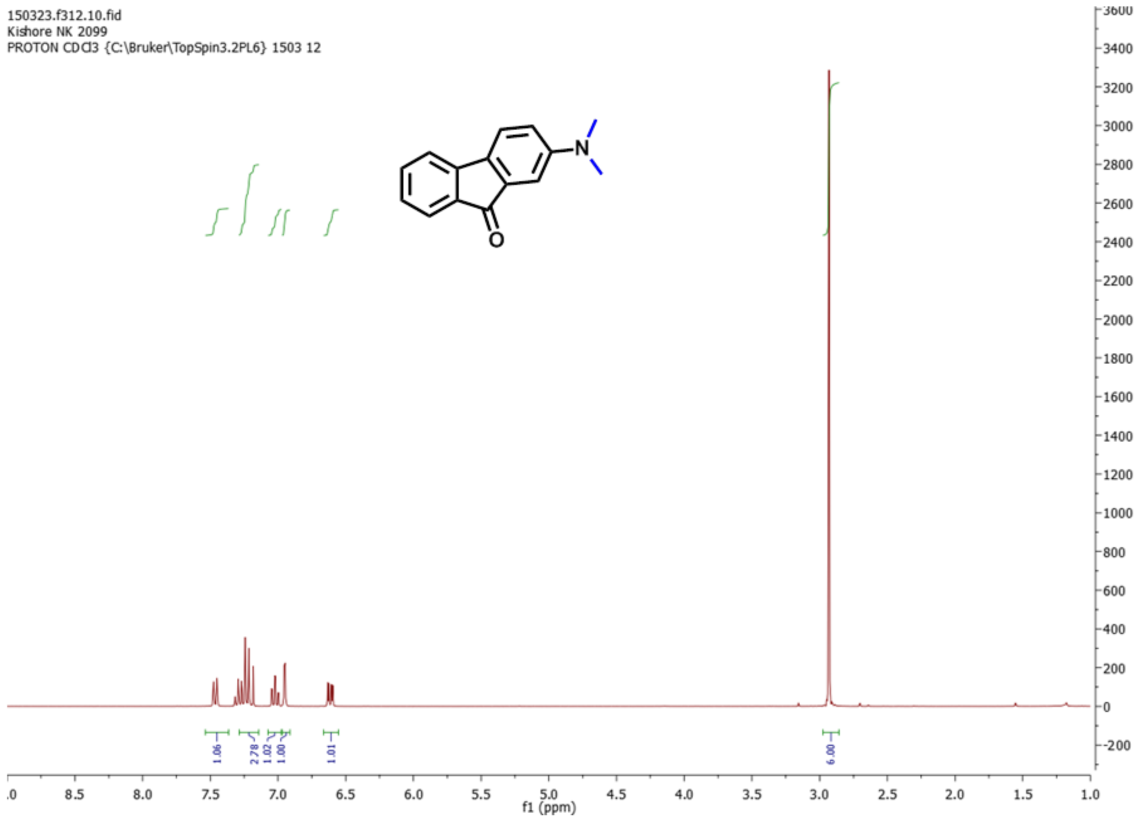

Supplementary Figure 77. ^{1}H NMR of Dimethylaminofluorenone

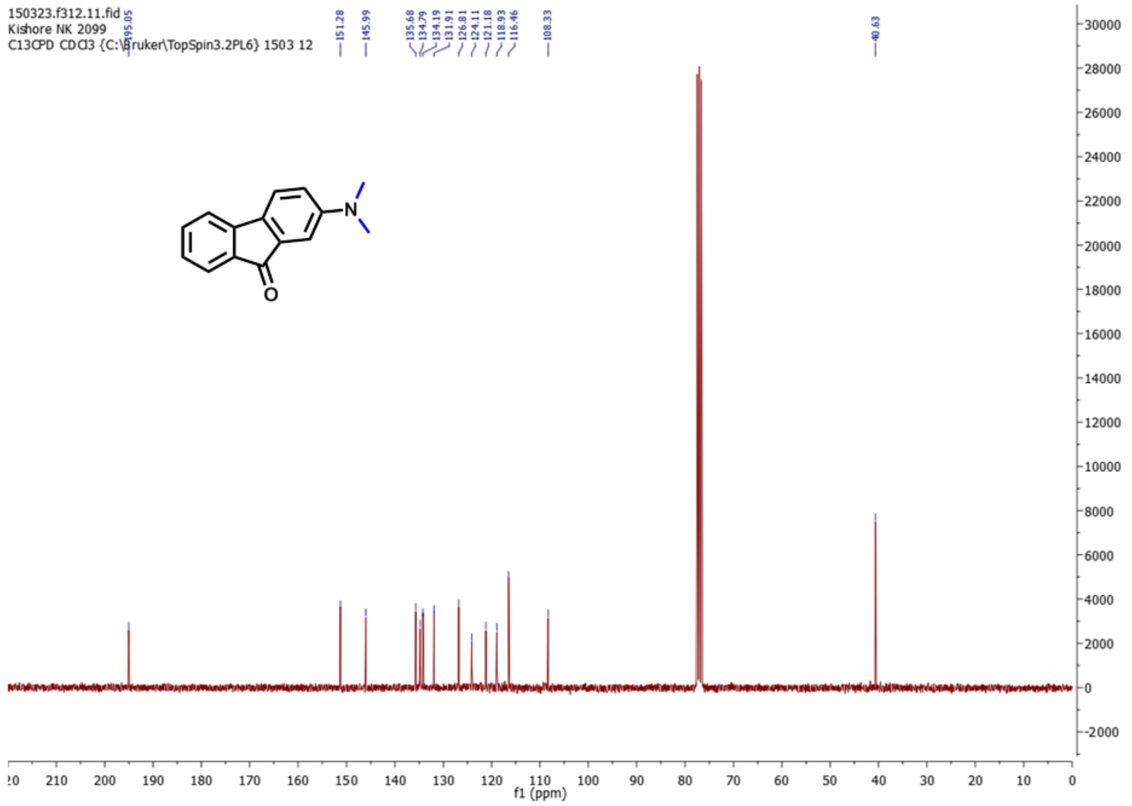

Supplementary Figure 78. ^{13}C NMR of Dimethylaminofluorenone

Qualitative Compound Report

**Instrument Name**	ESI-TOF/MS	**Date Filename**	D:\MassHunter\Data\1505\15051801.d
**Acq Method**	HRMS Pos oS.m	**Sample Name**	NK 2099
**DA Method**	HRMS.m	**Position**	Vial 51
**User Name**	Fischer	**Comment**	MeOH/0,1%HCOOH in H2O 90:10

Compound Table

Name	RT	Abund	Formula	Ion Mass	Ionization Mode
1	0.176	361479	C15 H13 N O	223.0997	Positive

MS Zoomed Spectrum

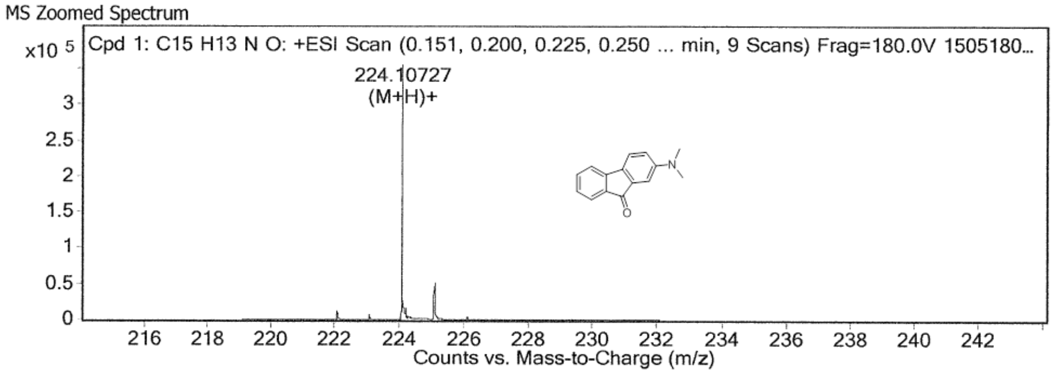

MS Spectrum Peak List

Ion	Abund	Formula	Calculated Mass	Measured Mass	Difference	Diff (ppm)
(M+H)+	361478.66	C15H13NO	224.10699	224.10727	-0.28	-1.26

--- End Of Report ---

Supplementary Figure 79. HRMS (High Resolution Mass Spectroscopy) of Dimethylaminofluorenone

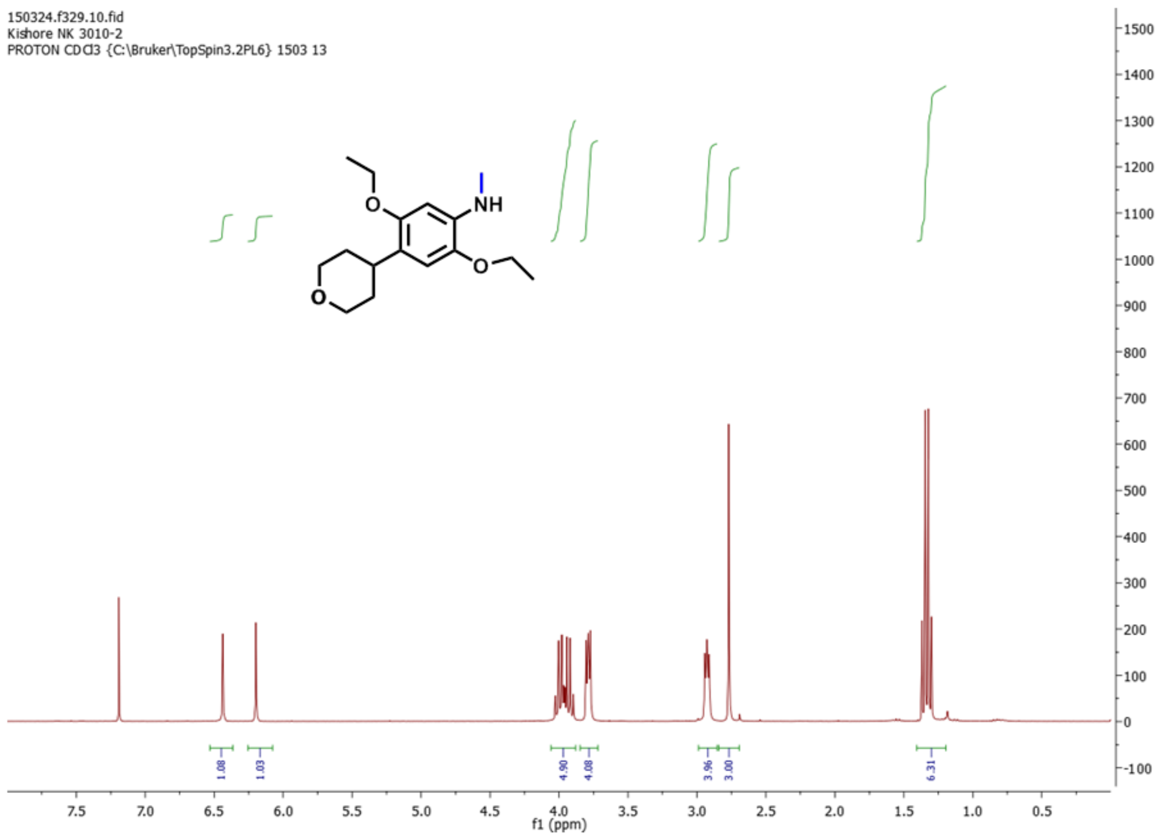

Supplementary Figure 80. ^{1}H NMR of 2,5-Diethoxy-*N*-methyl-4-(tetrahydro-2*H*-pyran-4-yl)aniline

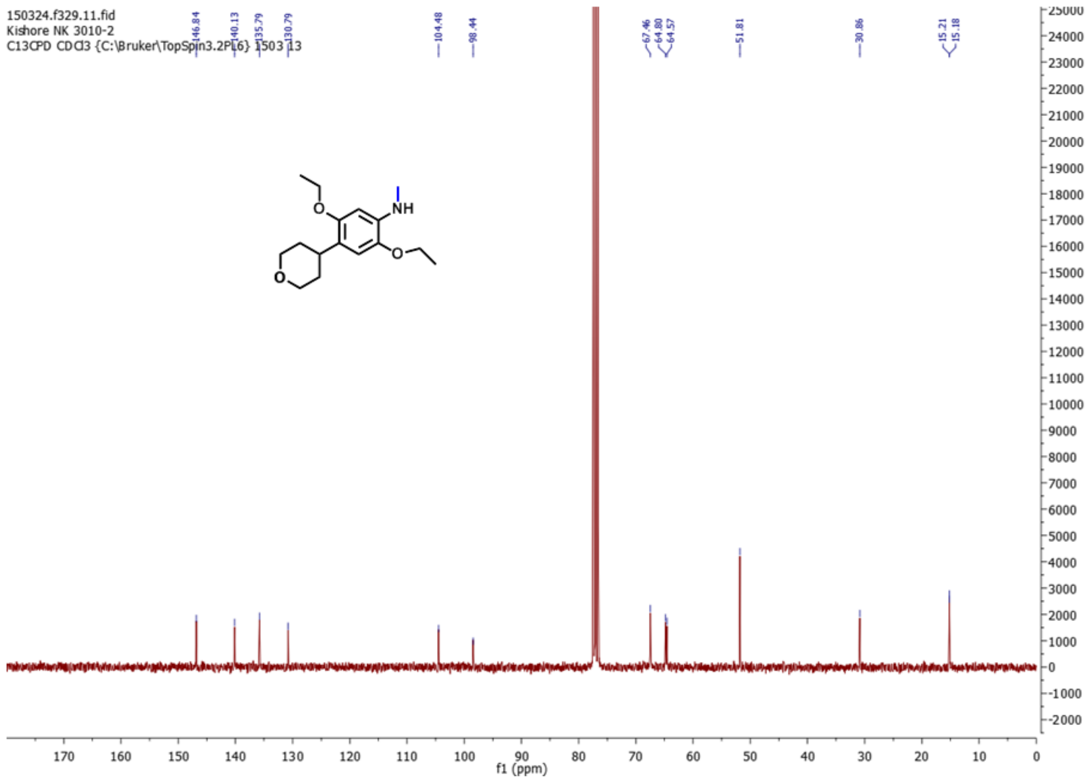

Supplementary Figure 81. ^{13}C NMR of 2,5-Diethoxy-*N*-methyl-4-(tetrahydro-2*H*-pyran-4-yl)aniline

Qualitative Compound Report

**Instrument Name**	ESI-TOF/MS	**Date Filename**	D:\MassHunter\Data\1505\15052013.d
**Acq Method**	HRMS Pos oS.m	**Sample Name**	NK3010-2
**DA Method**	HRMS.m	**Position**	Vial 74
**User Name**	Fischer	**Comment**	MeOH/0,1%HCOOH in H2O 90:10

Compound Table

Name	RT	Abund	Formula	Ion Mass	Ionization Mode
1	0.179	586304	C15 H23 N2 O3	279.1709	Positive

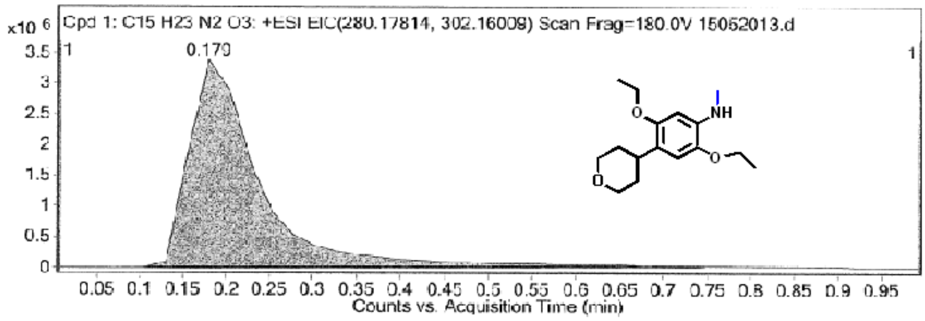

MS Zoomed Spectrum

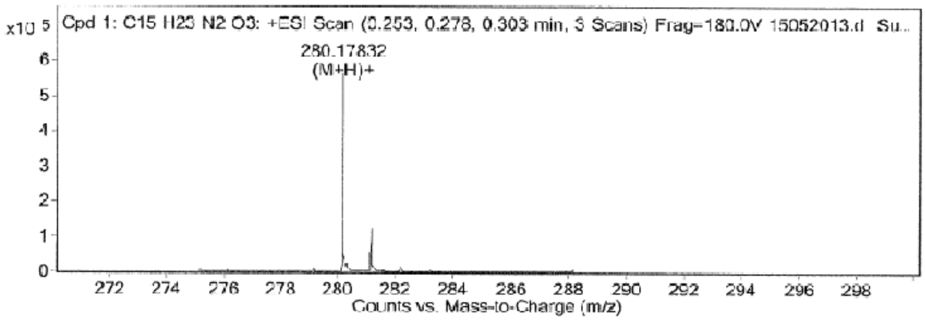

MS Spectrum Peak List

Ion	Abund	Formula	Calculated Mass	Measured Mass	Difference	Diff (ppm)
(M+H)+	586303.69	C15H23N2O3	280.17814	280.17832	-0.17	-0.62

--- End Of Report ---

Supplementary Figure 82. HRMS (High Resolution Mass Spectroscopy) of 2,5-Diethoxy-*N*-methyl-4-(tetrahydro-2*H*-pyran-4-yl)aniline

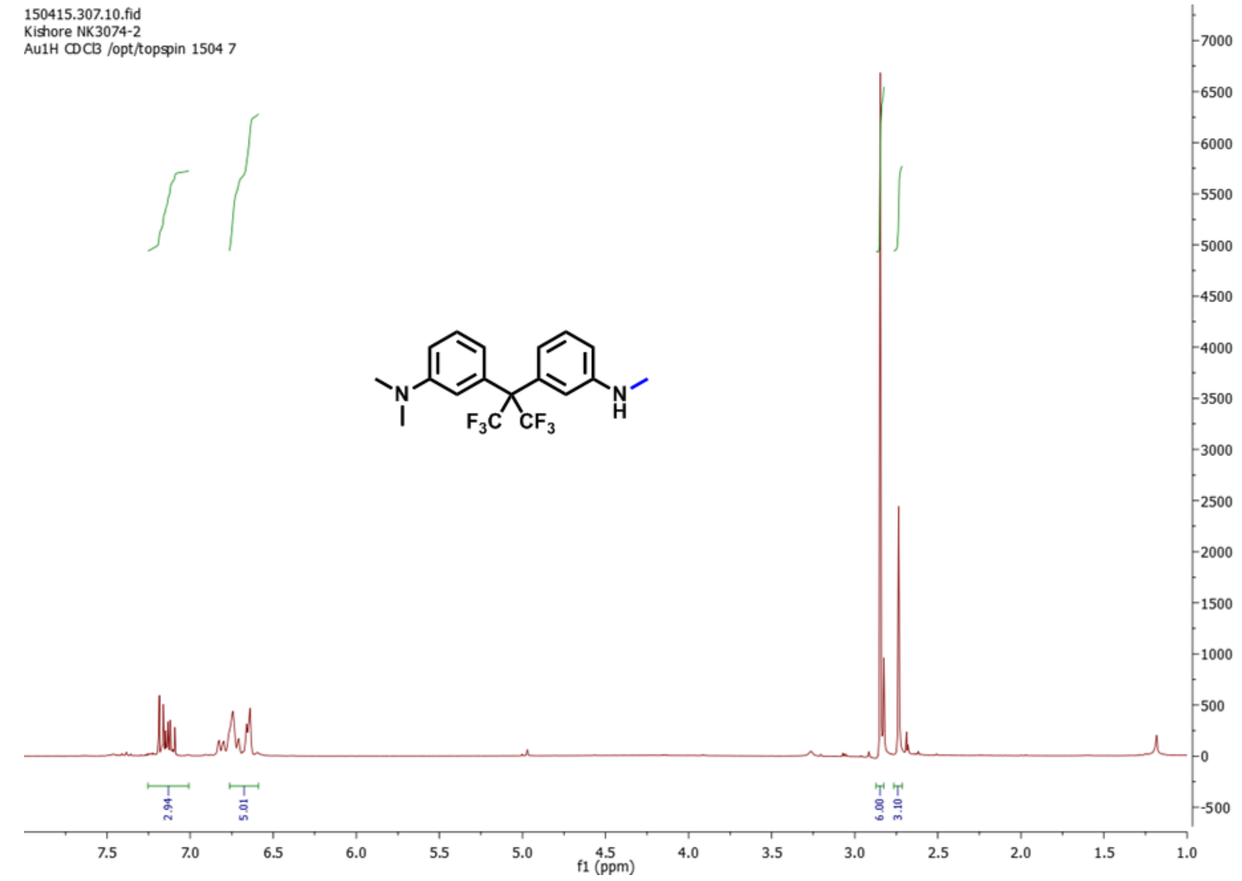

Supplementary Figure 83. ^{1}H NMR of 3-(1,1,1,3,3,3-hexafluoro-2-(3-(methylamino)phenyl)propan-2-yl)-N,N-dimethylaniline

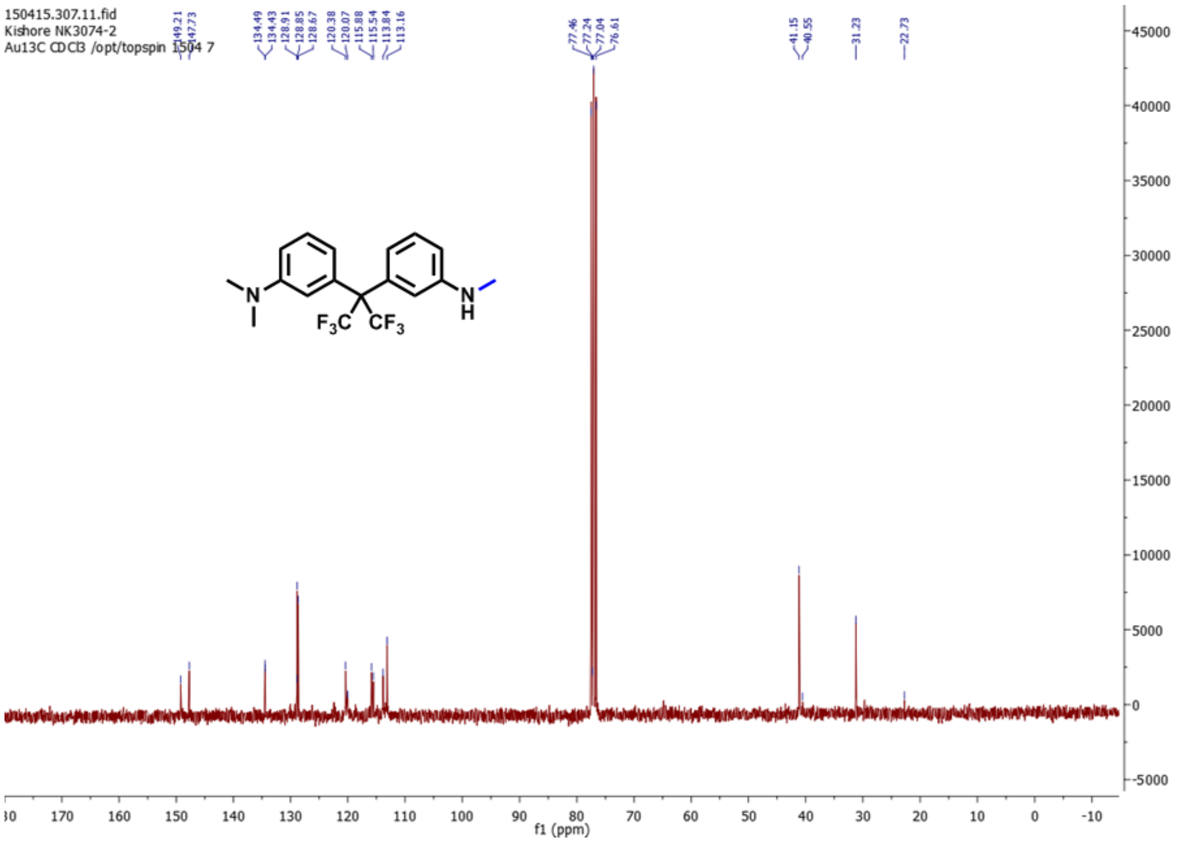

Supplementary Figure 84. ^{13}C NMR 3-(1,1,1,3,3,3-hexafluoro-2-(3-(methylamino)phenyl)propan-2-yl)-N,N-dimethylaniline

Qualitative Compound Report

**Instrument Name**	ESI-TOF/MS	**Date Filename**	D:\MassHunter\Data\1505\15051906.d
**Acq Method**	HRMS Pos cS.m	**Sample Name**	NK3074-2
**DA Method**	HRMS.m	**Position**	Vial 76
**User Name**	Fischer	**Comment**	MeOH/0,1%HCOOH in H2O 90:10

Compound Table

Name	RT	Abund	Formula	Ion Mass	Ionization Mode
1	0.169	1051688	C18 H18 F6 N2	376.1374	Positive

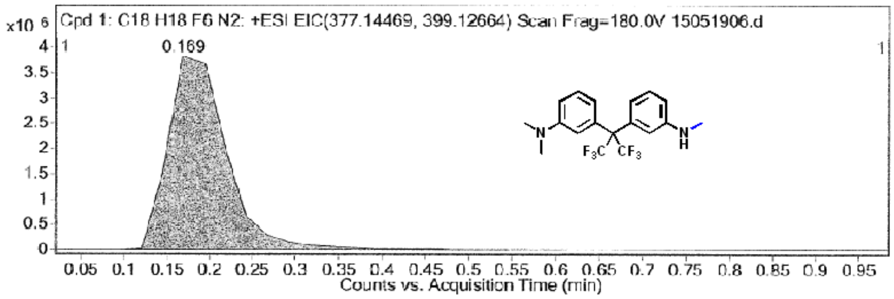

MS Zoomed Spectrum

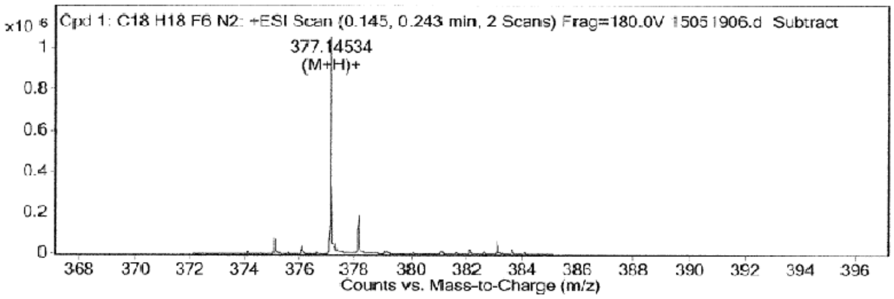

MS Spectrum Peak List

Ion	Abund	Formula	Calculated Mass	Measured Mass	Difference	Diff (ppm)
(M+H)+	1051687.75	C18H18F6N2	377.14469	377.14534	-0.65	-1.71

--- End Of Report ---

Supplementary Figure 85. HRMS (High Resolution Mass Spectroscopy) of 3-(1,1,1,3,3,3-hexafluoro-2-(3-(methylamino)phenyl)propan-2-yl)-N,N-dimethylaniline

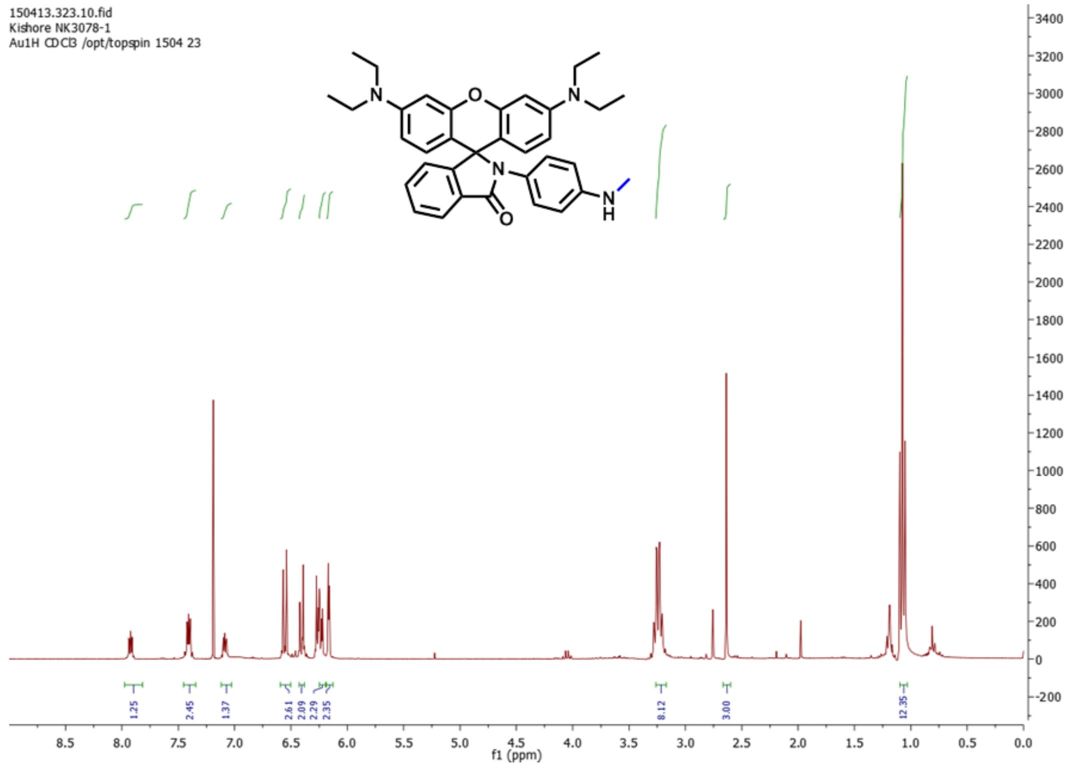

Supplementary Figure 86. ^{1}H NMR of 3',6'-Bis(diethylamino)-2-(4-(methylamino)phenyl)spiro[isoindoline-1,9'-xanthen]-3-one

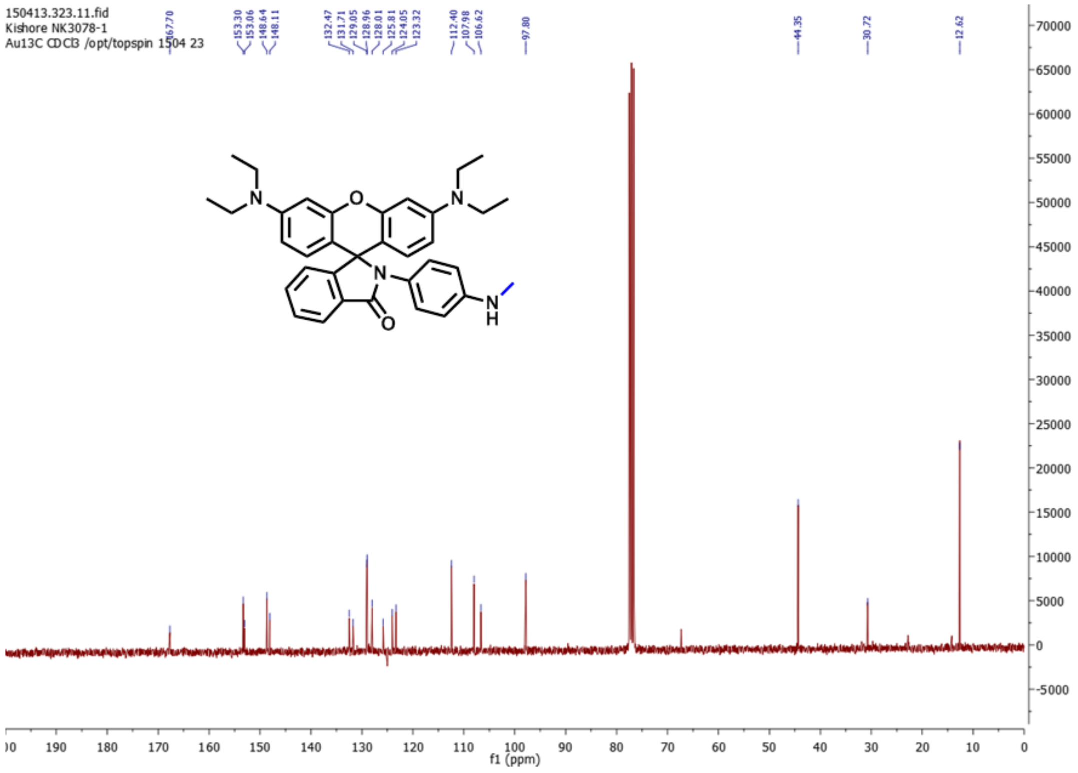

Supplementary Figure 87. ^{13}C NMR of 3',6'-Bis(diethylamino)-2-(4-(methylamino)phenyl)spiro[isoindoline-1,9'-xanthen]-3-one

Qualitative Compound Report

**Instrument Name**	ESI-TOF/MS	**Date Filename**	D:\MassHunter\Data\1505\15052020.d
**Acq Method**	HRMS Pos oS.m	**Sample Name**	NK3078-1
**DA Method**	HRMS.m	**Position**	Vial 62
**User Name**	Fischer	**Comment**	MeOH/0,1%HCOOH in H2O 90:10

Compound Table

Name	RT	Abund	Formula	Ion Mass	Ionization Mode
1	0.183	121883	C35 H38 N4 O2	546.2995	Positive

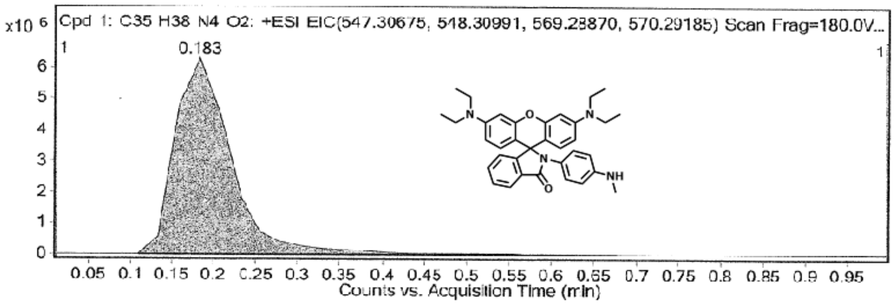

MS Zoomed Spectrum

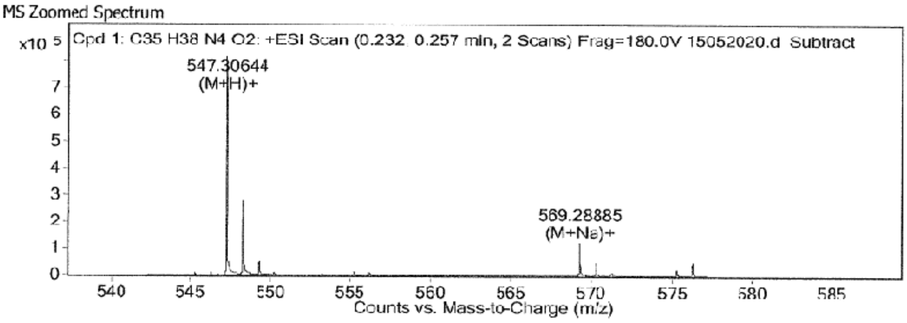

MS Spectrum Peak List

Ion	Abund	Formula	Calculated Mass	Measured Mass	Difference	Diff (ppm)
(M+H)+	817691.31	C35H38N4O2	547.30675	547.30644	0.32	0.58
(M+Na)+	121882.58	C35H38N4O2	569.2887	569.28885	-0.15	-0.27

Supplementary Figure 88. HRMS (High Resolution Mass Spectroscopy) of 3',6'-Bis(diethylamino)-2-(4-(methylamino)phenyl)spiro[isoindoline-1,9'-xanthen]-3-one

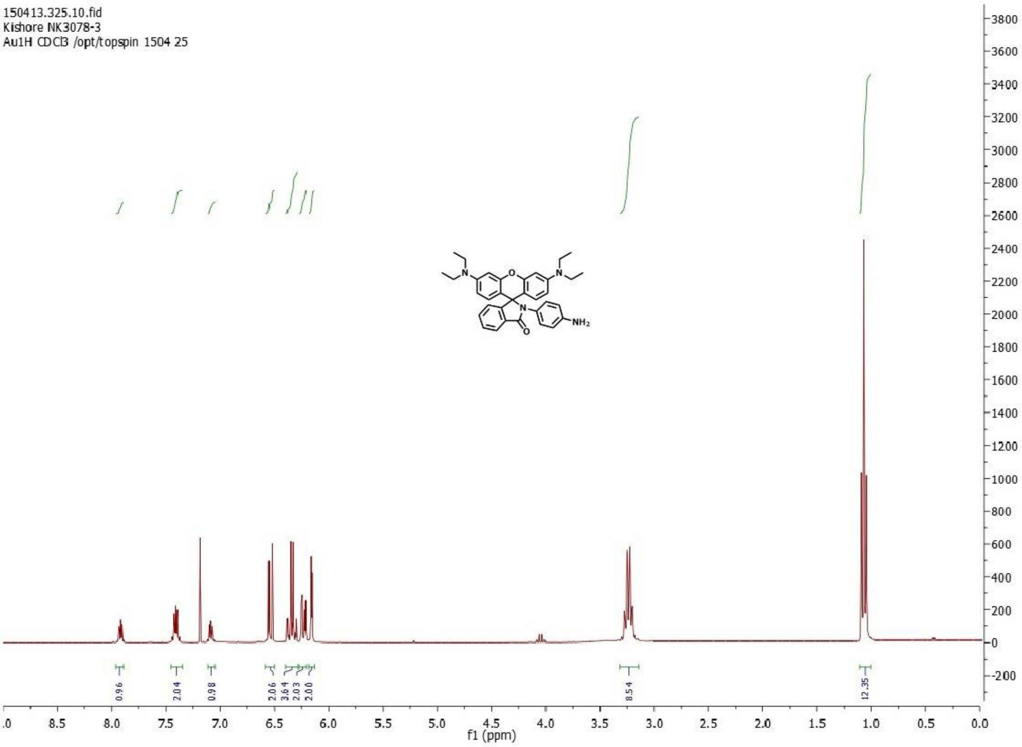

Supplementary Figure 89. ^{1}H NMR of 2-(4-Aminophenyl)-3',6'-bis(diethylamino)spiro[isoindoline-1,9'-xanthen]-3-one

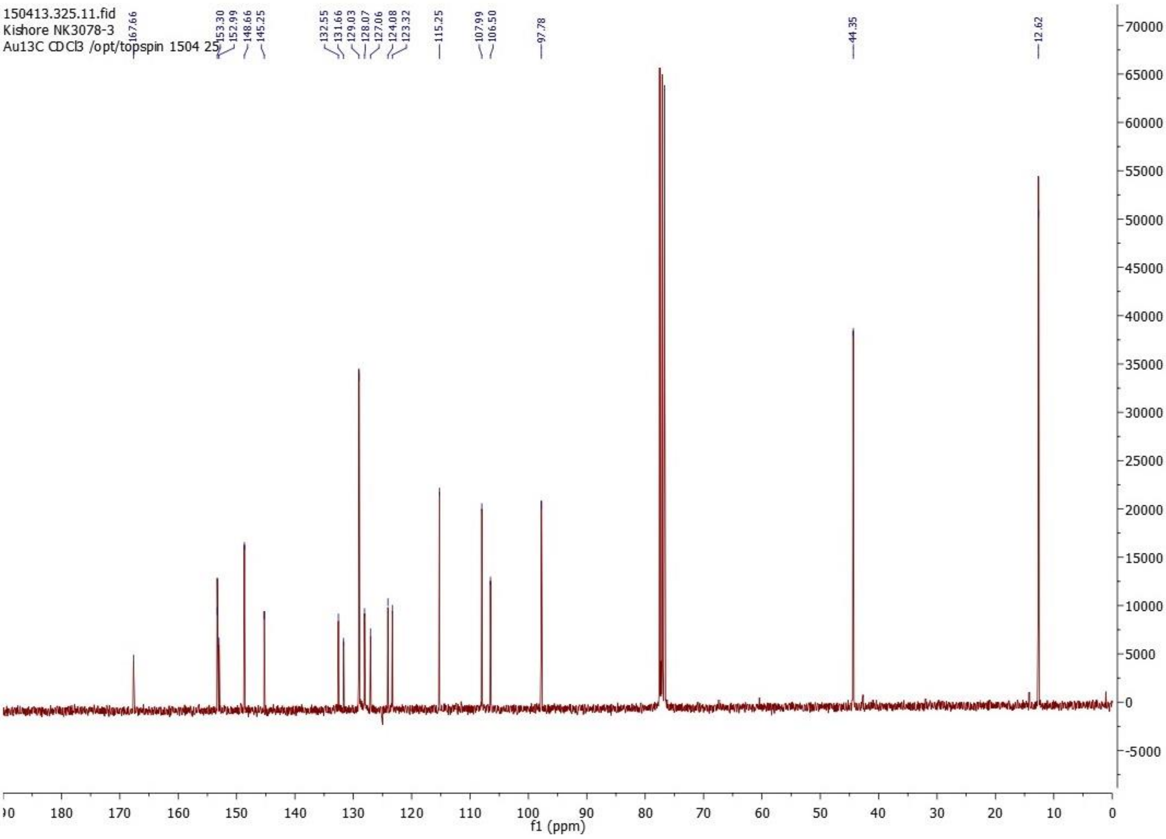

Supplementary Figure 90. ^{13}C NMR of 2-(4-Aminophenyl)-3',6'-bis(diethylamino)spiro[isoindoline-1,9'-xanthen]-3-one

Qualitative Compound Report

**Instrument Name**	ESI-TOF/MS	**Date Filename**	D:\MassHunter\Data\1505\15052023.d
**Acq Method**	HRMS Pos oS.m	**Sample Name**	NK3078-3
**DA Method**	HRMS.m	**Position**	Vial 65
**User Name**	Fischer	**Comment**	MeOH/0,1%HCOOH in H2O 90:10

Compound Table

Name	RT	Abund	Formula	Ion Mass	Ionization Mode
1	0.189	46026	C34 H36 N4 O2	532.2838	Positive

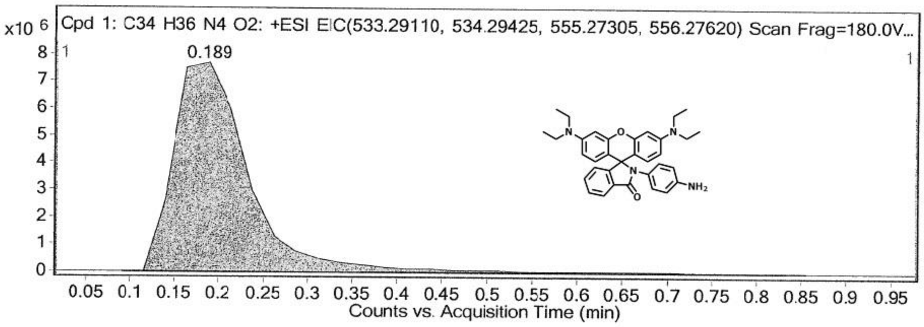

MS Zoomed Spectrum

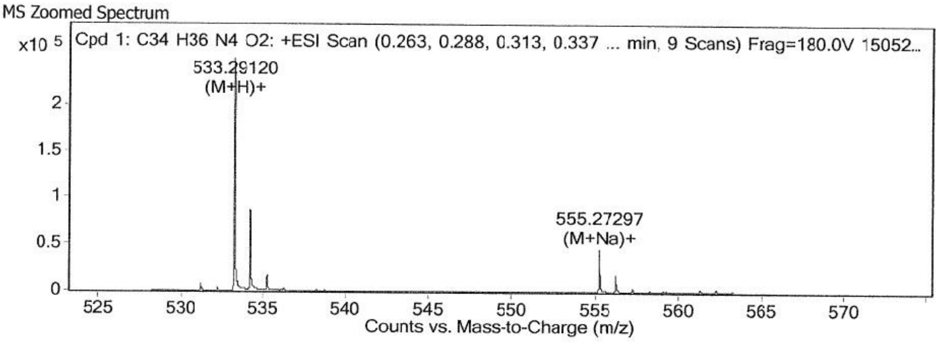

MS Spectrum Peak List

Ion	Abund	Formula	Calculated Mass	Measured Mass	Difference	Diff (ppm)
(M+H)+	249916.94	C34H36N4O2	533.2911	533.2912	-0.1	-0.18
(M+Na)+	46025.71	C34H36N4O2	555.27305	555.27297	0.08	0.14

--- End Of Report ---

Supplementary Figure 91. HRMS (High Resolution Mass Spectroscopy) of 2-(4-Aminophenyl)-3',6'-bis(diethylamino)spiro[isoindoline-1,9'-xanthen]-3-one

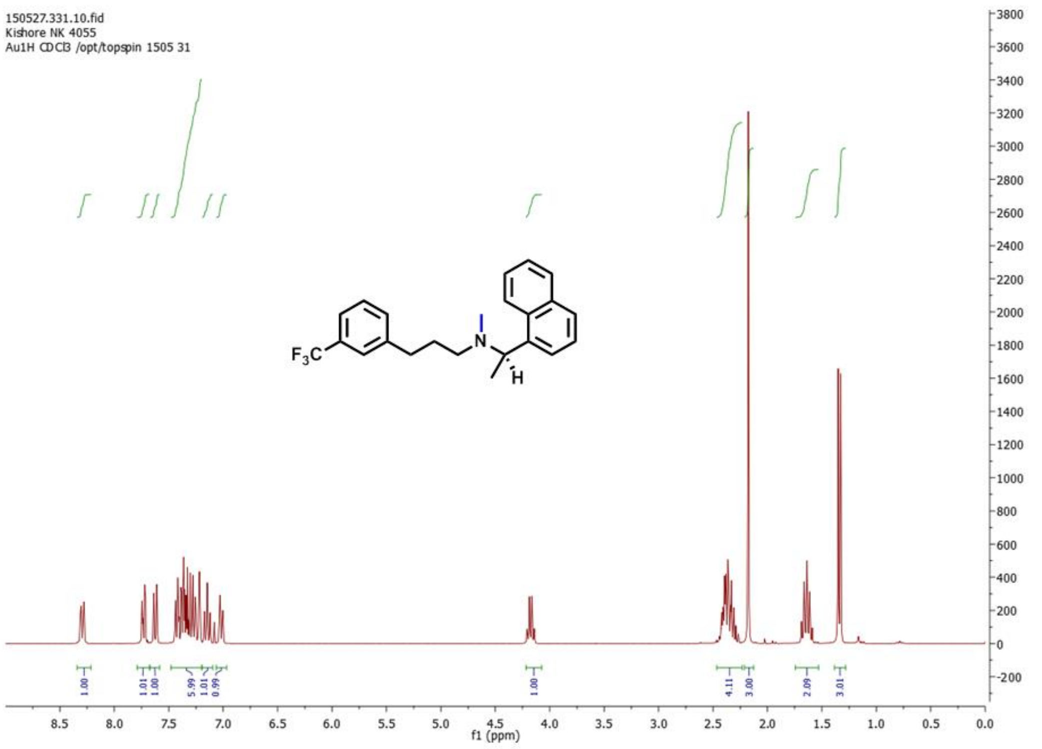

Supplementary Figure 92. ^{1}H NMR of Cinacalcet-NMe

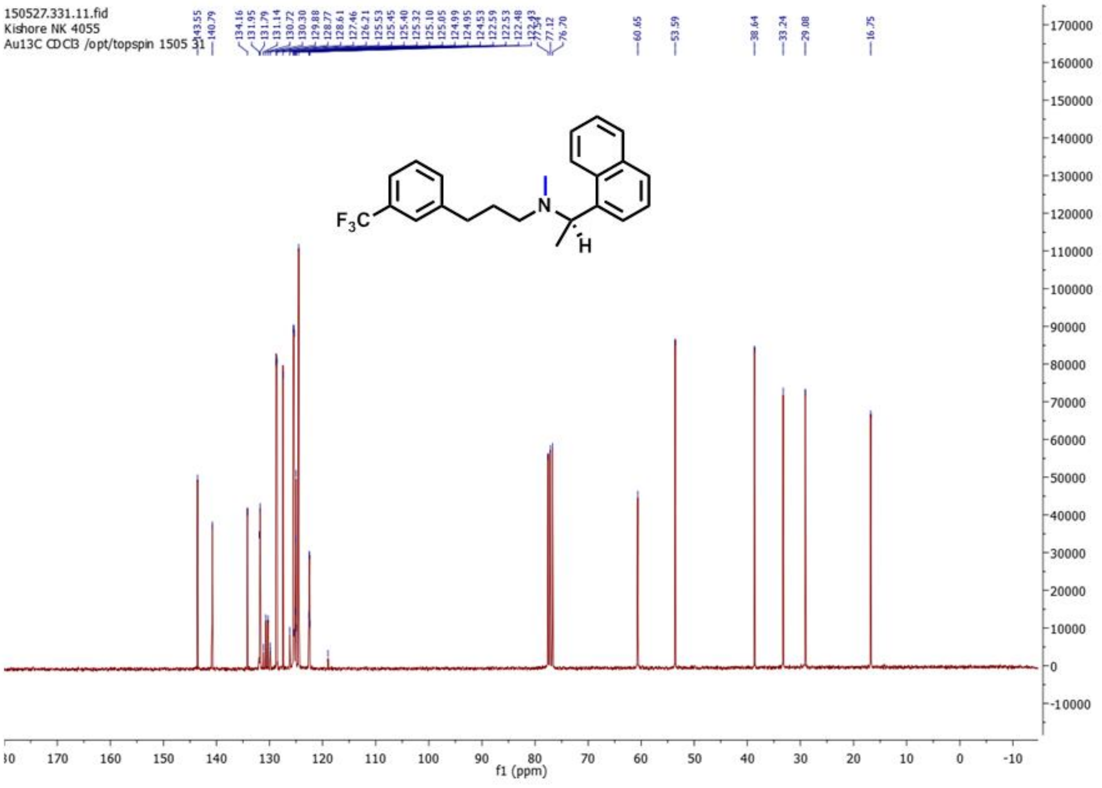

Supplementary Figure 93. ^{13}C NMR of Cinacalcet-NMe

File : D:\Xcalibur\data\1507\15072303hrei-av2.RAW
Full ms [351.500 - 385.500] - Range: 371.000 - 371.500
Scan No. 1 of 1

Mass	Absolute Intensity	Relative Intensity	Theoretical Mass	Delta [ppm]	Delta [mmu]	RDB	Composi
371.18477	3616292	24.2	371.18554	-2.1	-0.8	11.0	$C_{23} H_{24} N$

Supplementary Figure 94. HRMS (High Resolution Mass Spectroscopy) of Cinacalcet-NMe

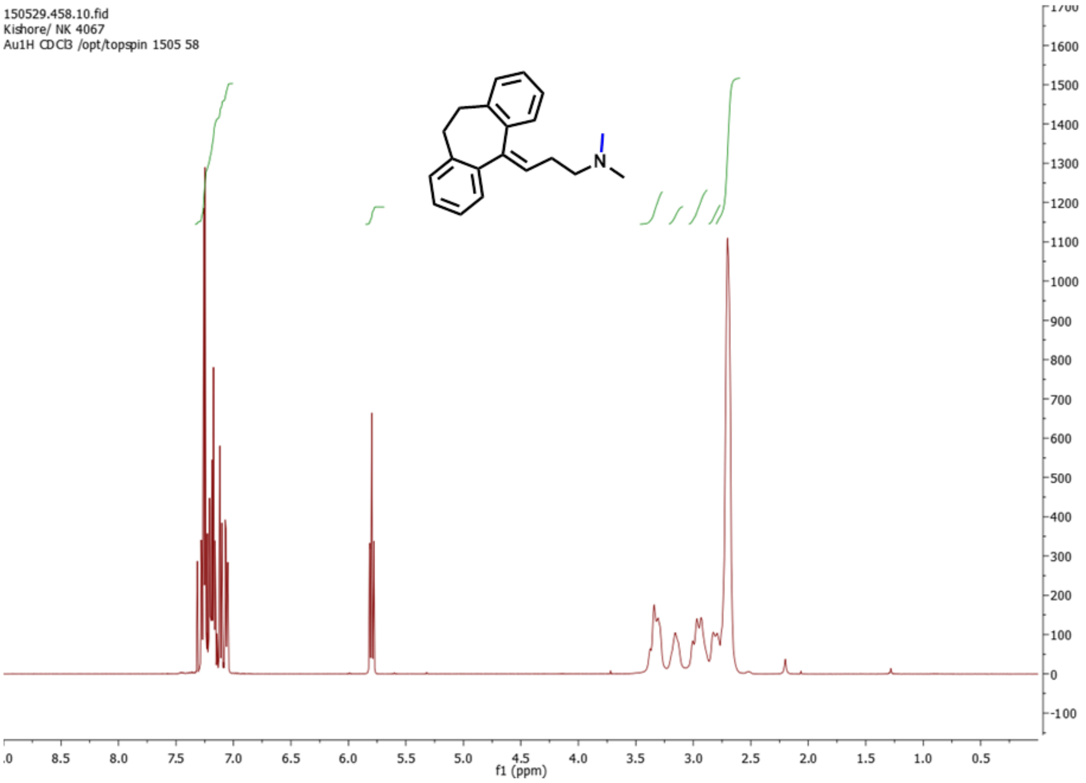

Supplementary Figure 95. ^{1}H NMR of Amitriptyline

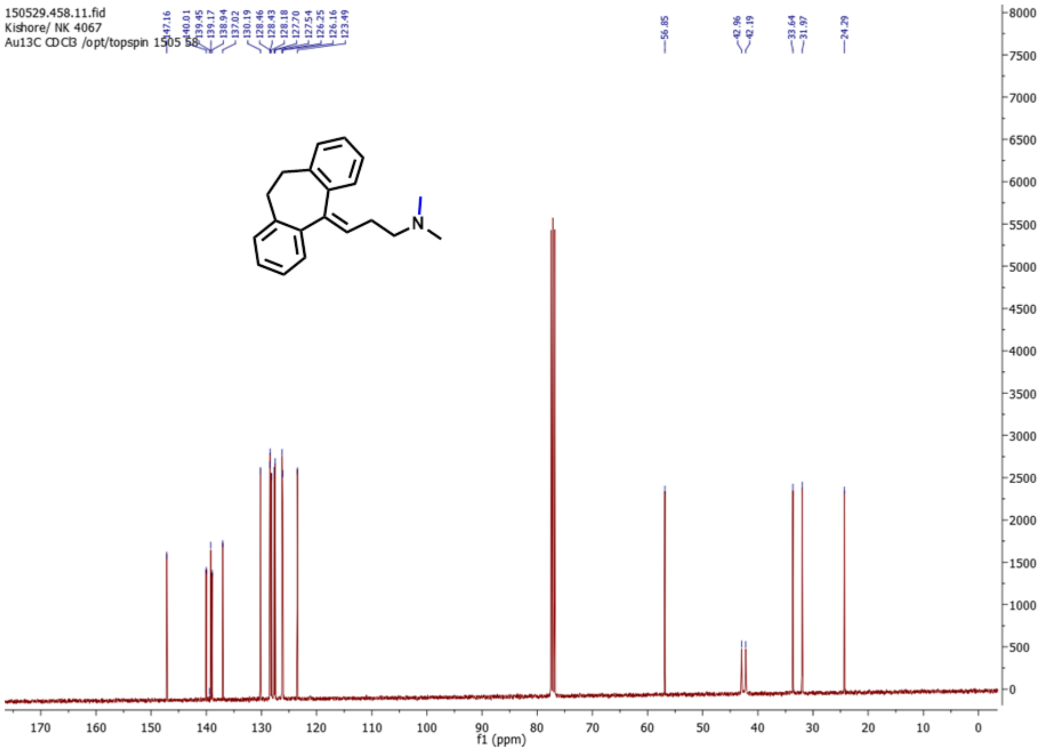

Supplementary Figure 96. ^{13}C NMR of Amitriptyline

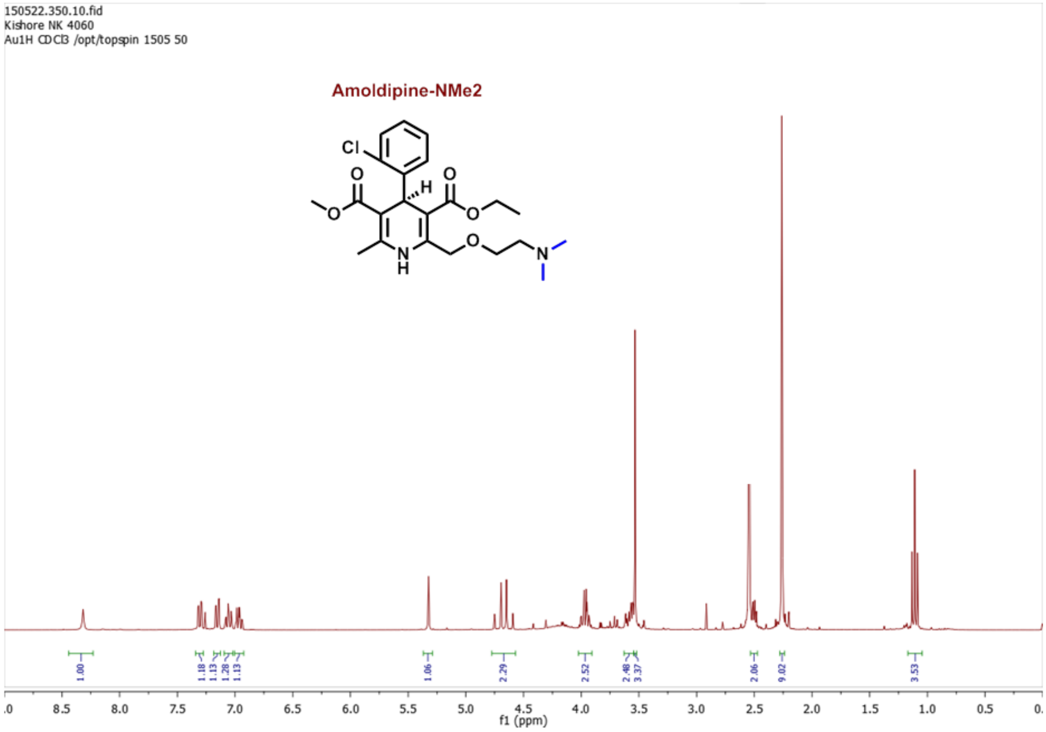

Supplementary Figure 97. ^{1}H NMR of Amoldipine-NMe2

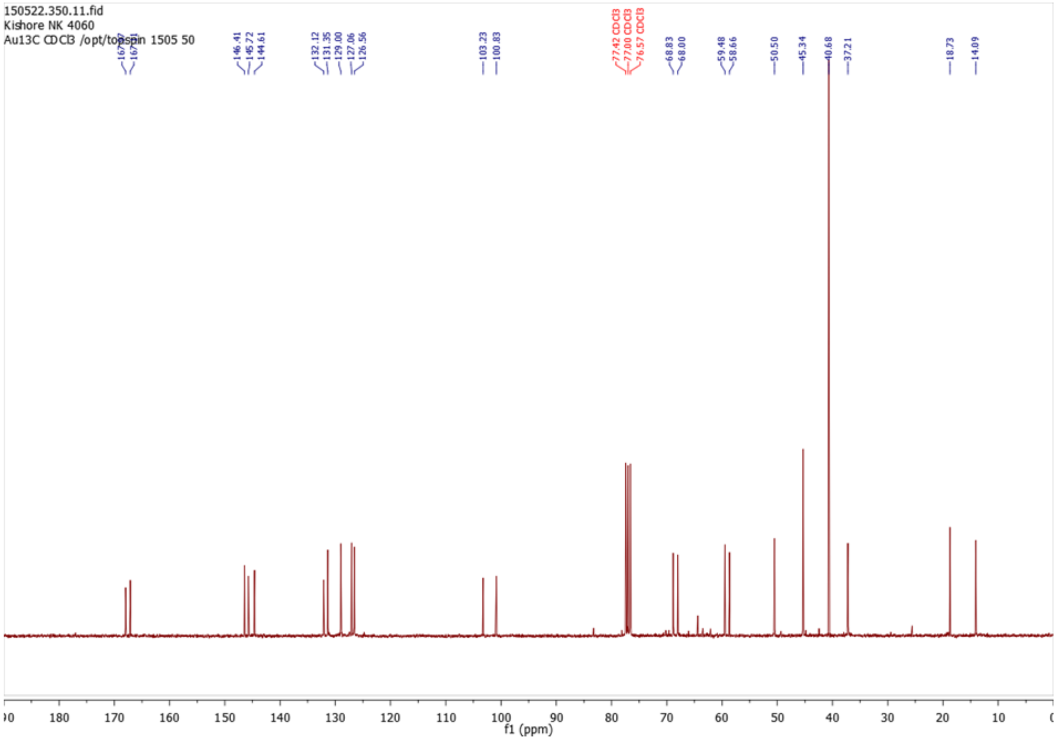

Supplementary Figure 98. ^{13}C NMR of Amoldipine-NMe2

File : D:\Xcalibur\data\1507\15072304hrei-av2.RAW
Full ms [413.500 - 447.500] - Range: 413.500 - 447.500
Scan No. 1 of 1

Mass	Absolute Intensity	Relative Intensity	Theoretical Mass	Delta [ppm]	Delta [mmu]	RDB	Composition
416.97456	422721	40.0					
430.97231	1056355	100.0					
436.17600	769985	72.9	436.17595	0.1	0.1	9.0	$C_{22} H_{29} O_5 N_2 Cl_1$
438.17427	274939	26.0	438.17300	2.9	1.3	9.0	$C_{22} H_{29} O_5 N_2 {}^{37}Cl_1$
442.97231	828611	78.4					

Supplementary Figure 99. HRMS (High Resolution Mass Spectroscopy) of Amoldipine-NMe2

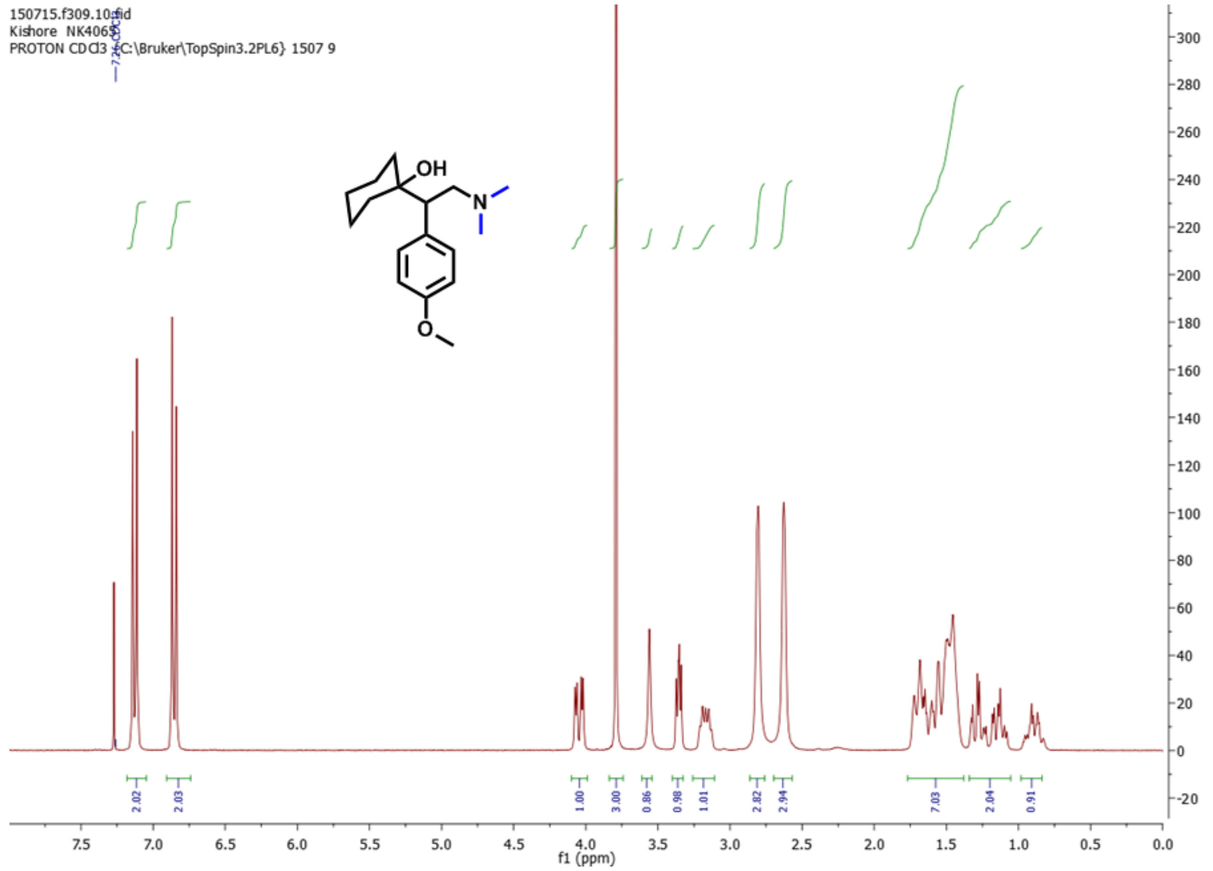

Supplementary Figure 100. ^{1}H NMR of Venlafaxine

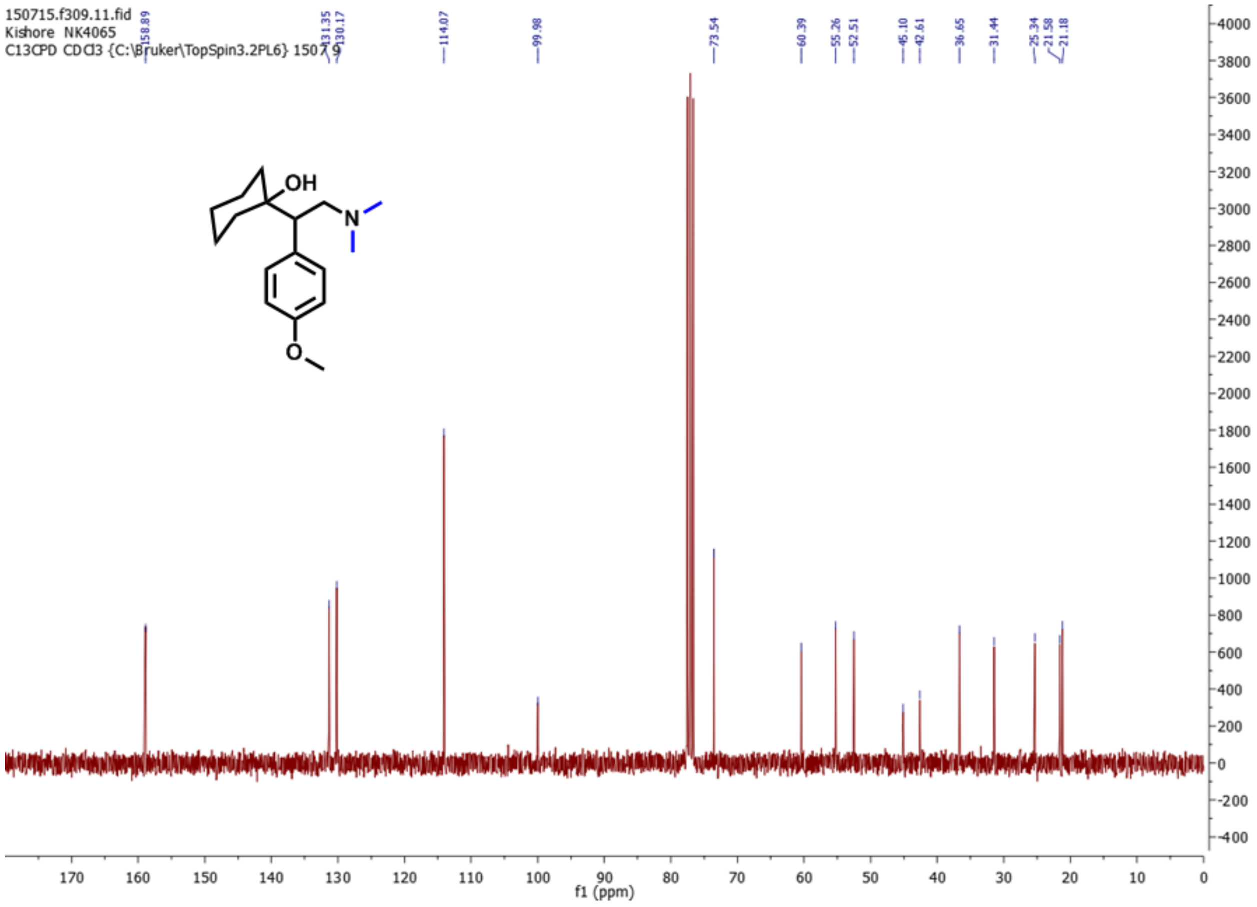

Supplementary Figure 101. ^{13}C NMR of Venlafaxine

Qualitative Analysis Report

**Instrument Name**	LCMSD	**Data Filename**	D:\Chem32\1\Data\1508\15082622.D
**Acq Method**	SCAN Pos_oS.M	**Sample Name**	NK 4065
**DA Method**	Standard.m	**Position**	Vial 41
**User Name**	SYSTEM	**Comment**	MeOH/0,1%HCOOH in H2O 90:10

User Spectra

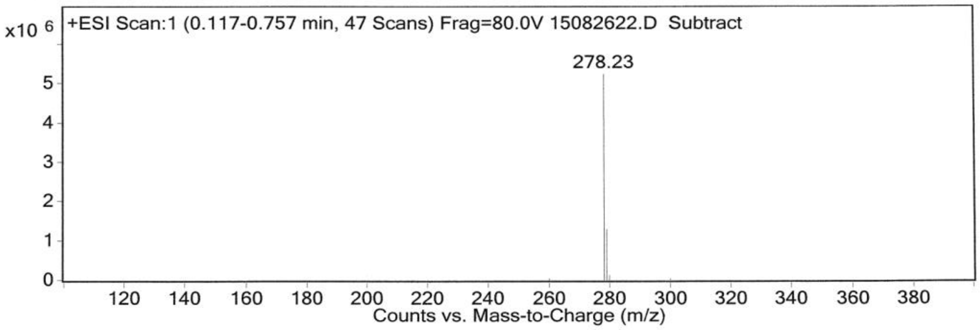

Supplementary Figure 102. HRMS (High Resolution Mass Spectroscopy) of Venlafaxine

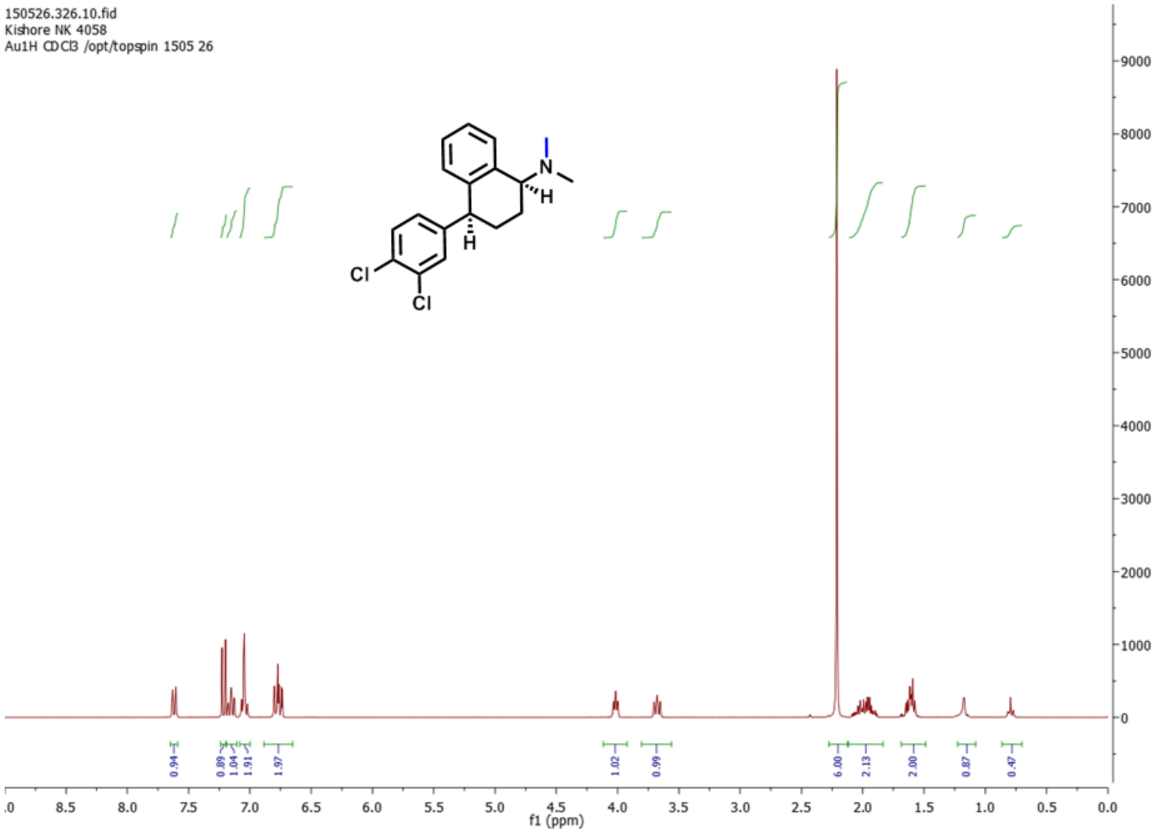

Supplementary Figure 103. ^{1}H NMR of Sertraline-NMe

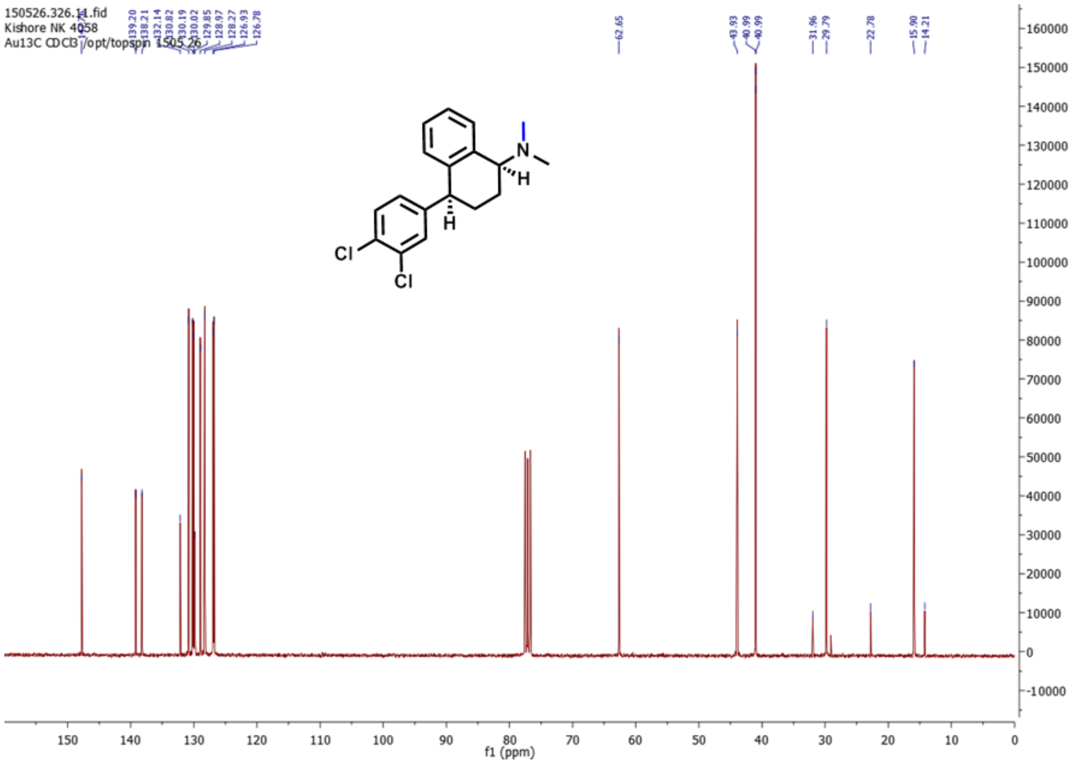

Supplementary Figure 104. ^{13}C NMR of Sertraline-NMe

File : D:\Xcalibur\data\1507\15072302hrei-av3.RAW
Full ms [301.500 - 335.500] - Range: 301.500 - 335.500
Scan No. 1 of 1

Mass	Absolute Intensity	Relative Intensity	Theoretical Mass	Delta [ppm]	Delta [mmu]	RDB	Composition
319.08822	3391108	100.0	319.08891	-2.1	-0.7	9.0	$C_{18} H_{19} N_1 Cl_2$
321.08622	2197534	64.8	321.08596	0.8	0.3	9.0	$C_{18} H_{19} N_1 Cl_1 {}^{37}Cl_1$

Supplementary Figure 105. HRMS (High Resolution Mass Spectroscopy) of Sertraline-NMe
